# Novel lamprey antibody recognizes terminal sulfated galactose epitopes on mammalian glycoproteins

**DOI:** 10.1038/s42003-021-02199-7

**Published:** 2021-06-03

**Authors:** Tanya R. McKitrick, Steffen M. Bernard, Alexander J. Noll, Bernard C. Collins, Christoffer K. Goth, Alyssa M. McQuillan, Jamie Heimburg-Molinaro, Brantley R. Herrin, Ian A. Wilson, Max D. Cooper, Richard D. Cummings

**Affiliations:** 1 Department of Surgery, Beth Israel Deaconess Medical Center, Harvard Medical School, Boston, MA USA; 2 Department of Integrative Structural and Computational Biology, The Scripps Research Institute, La Jolla, CA USA; 3 Department of Pathology and Laboratory Medicine, Emory Vaccine Center, Emory University School of Medicine, Atlanta, GA USA; 4 The Skaggs Institute for Chemical Biology, The Scripps Research Institute, La Jolla, CA USA; 5 Present Address: Enteric Disease Department, Naval Medical Research Center, Silver Spring, MD USA; 6 Present Address: Department of Biomedical Sciences, University of Copenhagen, Copenhagen, Denmark; 7 Present Address: Acceleron Pharma, Boston, MA USA

**Keywords:** Glycobiology, Glycoconjugates

## Abstract

The terminal galactose residues of N- and O-glycans in animal glycoproteins are often sialylated and/or fucosylated, but sulfation, such as 3-O-sulfated galactose (3-O-SGal), represents an additional, but poorly understood modification. To this end, we have developed a novel sea lamprey variable lymphocyte receptor (VLR) termed O6 to explore 3-O-SGal expression. O6 was engineered as a recombinant murine IgG chimera and its specificity and affinity to the 3-O-SGal epitope was defined using a variety of approaches, including glycan and glycoprotein microarray analyses, isothermal calorimetry, ligand-bound crystal structure, FACS, and immunohistochemistry of human tissue macroarrays. 3-O-SGal is expressed on N-glycans of many plasma and tissue glycoproteins, but recognition by O6 is often masked by sialic acid and thus exposed by treatment with neuraminidase. O6 recognizes many human tissues, consistent with expression of the cognate sulfotransferases (GAL3ST-2 and GAL3ST-3). The availability of O6 for exploring 3-O-SGal expression could lead to new biomarkers for disease and aid in understanding the functional roles of terminal modifications of glycans and relationships between terminal sulfation, sialylation and fucosylation.

## Abstract (web summary)

McKitrick et al engineer and characterize a novel lamprey antibody that recognizes terminal sulfated galactose epitopes on mammalian glycoproteins. This provides a new tool for the exploration of glycoprotein sulfation in mammalian cells, which could aid in the understanding of its functional role and potentially lead to identification of disease biomarkers.

## Introduction

Glycosylation is an essential process that occurs in all known organisms and mediates multiple biological processes such as cell–cell interactions, cellular integrity, cellular signaling, and immune functions^1^. Glycans on glycoproteins and glycolipids, as well as glycosaminoglycans, can be further modified by the addition of phosphates or sulfates to particular glycan residues, which can result in divergent functional outcomes^2–5^. Terminal modifications of glycans are also important in their functions, and those, such as sialylation and fucosylation typically, are found on terminal galactose (Gal) or penultimate N-acetylglucosamine (GlcNAc) residues^6^. The addition of a negatively charged moiety, including sulfate or phosphate, to a glycan can have a substantial impact on the physiological landscape of the cell membrane, and can contribute significantly to protein–glycan interactions^7–10^. Sulfated glycans in particular play diverse roles, and the loss of these modifications can result in disease or altered functions^2,5^.

A particularly enigmatic modification is 3-O-sulfation of galactose (3-O-SGal) generated by members of the Gal3ST family, which use 3-phosphoadenosine-5’-phosphosulfate as the donor. 3-O-SGal is found in the glycosphingolipid sulfatide, as generated by Gal3ST1^11^. Such sulfation is essential for central nervous system maintenance, as shown by loss of myelin function and oligodendrocyte differentiation observed in Gal3ST1 knockout animals^12–15^, as well as being required for germ cell functions^16^. There is much less understanding, however, about the role(s) of 3-O-SGal in glycoproteins, where it has been reported that this modification is expressed in several glycoproteins, such as thyroglobulin^17^, thyroid stimulating hormone^18^, bovine peripheral myelin glycoprotein P0^19^, and mouse brain neural cell adhesion molecule^20^. The presence of 3-O-SGal is regulated by Gal3ST-2 and Gal3ST-3, both which can sulfate type-2 LacNAc (Galβ1-4GlcNAc)^21^ on N-glycans, whereas Gal3ST-4 prefers Galβ1-3GalNAc on O-glycans^21,22^. Despite its importance as a terminal glycan modification, 3-O-SGal has been difficult to study and analyze because it is relatively labile and analysis commonly require special procedural modifications^23–26^. There are no reagents for directly exploring 3-O-SGal expression, thus its frequency and distribution in mammalian tissues and cells are poorly described. Interestingly, some approaches may miss the expression of 3-O-SGal, due to cross-reactivity of some plant lectins that are thought to bind only sialic acid, but can also bind to 3-O-SGal^27,28^.

To better define the expression of 3-O-SGal, we leveraged the unique adaptive immune system that has evolved in the sea lamprey, *Petromyzon marinus*. As antigen receptors, the lamprey utilize a family of highly diverse proteins called variable lymphocyte receptors (VLRs), which contain multiple, tandem leucine-rich repeat (LRR) motifs^29–31^. After antigenic stimulation, a population of lymphocytes termed VLRB cells, undergoes clonal expansion and differentiate into plasma-like cells that express cell surface antigen-specific VLRB proteins and also secrete them into the circulation^32–34^. The VLRB germline sequence is incomplete, and is flanked by numerous LRR modules that are incorporated into the mature VLRB transcript via a specialized gene conversion-like mechanism that is capable of generating >10^14^ distinct receptors^29,35,36^. The resulting VLRB protein is a 15–25 kDa, crescent-shaped protein with a continuous beta sheet where its concave surface forms the antigen binding site. Variation in antigen recognition is achieved by amino-acid sequence diversity located within the antigen binding site as well as the number of LRR segments that are incorporated into the mature protein.

Sea lampreys can induce robust VLRB responses to multiple glycan determinants upon immunization with a variety of glycan-bearing immunogens^37–39^. In particular, analysis of immunized plasma on glycan microarrays revealed that lampreys can generate VLRBs specific for a variety of sulfated glycans, regardless of the immunogen^39^. Herein, we report on the characterization of a lamprey VLRB that is specific for terminal 3-O-SGal, and was isolated from a lamprey immunized with human type O erythrocytes. This VLRB, termed O6, is the first reagent to our knowledge that specifically recognizes the 3-O-SGal determinant on glycoproteins with high sensitivity. The use of O6 to identify the presence of 3-O-SGal on blood glycoproteins and in different cells and tissues, and its masking by sialylation as shown here, demonstrate that O6 will be useful for glycomic analyses and for characterizing expression profiles and roles of 3-O-SGal in health and disease.

## Results

### Isolation, identification, sequencing, and cloning of O6

We identified O6 as an antibody that recognizes 3-O-SGal using a yeast surface display (YSD) library generated from immunization of lampreys with type O human erythrocytes^39^. Using methodology previously described, an amplified VLRB-specific cDNA library was transfected into the Saccharomyces EBY100 yeast strain with the pCT-ESO-BDNF expression vector^39–41^. Using this expression platform, individual VLRB proteins are induced to the cell surface by a galactose promoter, where they are tethered to the endogenous cell surface Aga1p and Aga2p proteins. To identify antigen-specific VLRB clones, we incubated the induced VLRB YSD library with biotinylated intact erythrocytes and enriched by positive selection using streptavidin coupled magnetic beads (MACS). We further enriched this library by FACS of the VLRB-induced yeast bound to erythrocytes. This enriched YSD library was incubated on an extensive glycan microarray from the Consortium for Functional Glycomics (CFG), where the induced VLRBs on yeast can bind directly to the sugars printed on the microarray surface^39^. After extensive washing, the bound clones were transferred to SD-CAA-agar plates and the VLRBs were sequenced. Yeast clones with unique VLRB sequences were individually screened by reexamination on the CFG array, and bound yeast clones were detected with an anti-Myc secondary antibody. The O6 VLRB was chosen because it exhibited unique specificity to 3-O-SGal, as described below, and was then permanently transduced into HEK293 cells via a lentiviral construct to produce the recombinant VLR- mouse IgG2a (O6-mFc) fusion protein.

### Screening of O6 on glycan microarrays

**Fig. 1:**
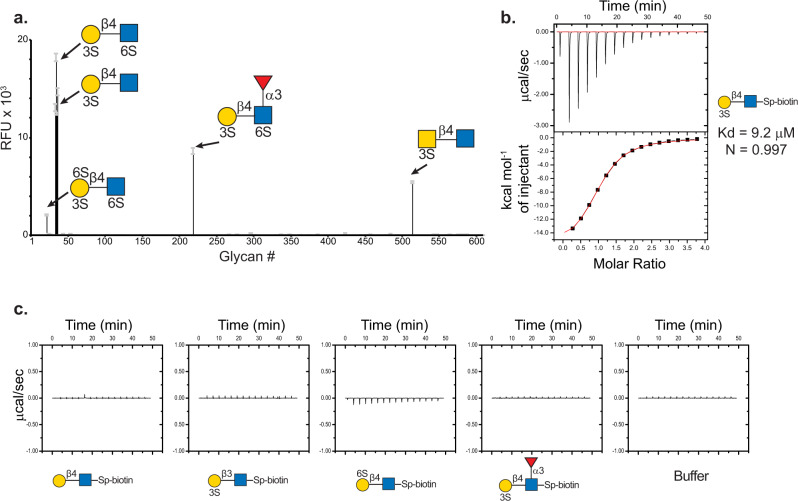
The specificity of VLR O6 as determined by glycan microarray and ITC. **a** O6-mFc chimeric protein screened on CFG mammalian glycan microarray at 10 µg/mL reveals the binding motif of O6 is (3 S)Galβ1-4GlcNAc. RFU relative fluorescence units, error bars = ±1 SD. **b** Monomeric VLR O6 binds to ligand with ~10 µM affinity. **c** No detectable binding curves were observed by ITC for the following structures: Galβ1-4GlcNAc, (3 S)Galβ1-3GlcNAc, (6 S)Galβ1-4GlcNAc and (3 S)Galβ1-4(Fucα1-3)GlcNAcβ-Sp. Yellow circle = Gal, yellow square = GalNAc, blue square = GlcNAc, red triangle = fucose, 3 S and 6 S = position of sulfate.

As previously mentioned, the O6 VLRB was discovered as a yeast clone bound to glycans containing 3-O-SGal on the CFG microarray (see Supplementary Data 1). To further explore its specificity, we expressed O6 as a recombinant mouse Fc chimeric protein and screened the soluble form on the CFG array at three different concentrations. Within the CFG array, 63 glycans are sulfated and 4 are phosphorylated, and O6 bound in a dose-dependent manner to several glycans carrying the terminal 3-O-SGal moiety. The highest signal intensities were observed for glycans containing the N-acetyllactosamine type II motif ((3 S)Galβ1-4GlcNAc) (Fig. 1a). O6 also bound to (3 S)GalNAcβ1-4GlcNAc with a 5-fold lower signal intensity, and binding to LacNAc (Galβ1-4GlcNAc) with additional sulfates on the 4th or 6th position of the Gal either prevented or greatly reduced the signal relative to (3 S)Galβ1-4GlcNAc (Supplementary Data 1). Glycans with 3-O-SGal and an additional 6-O-sulfate on the penultimate GlcNAc residue were bound by O6, whereas there was no recognition of sulfated terminal glucuronic acid (GlcA) and nonsulfated LacNAc glycans (Supplementary Table S1).

Notably, O6 did not bind to the glycosaminoglycan (GAG) disaccharides on the CFG array, which is a well-known class of glycans that carry sulfate moieties. To exclude the possibility that the VLRB was simply interacting with the presence of a negatively charged functional group, we also screened O6 on a microarray containing glycopeptides with tyrosine sulfate^42^. O6 did not bind to the glycosulfopeptides on this array (Supplementary Data 2), demonstrating that O6 does not bind sulfated tyrosine residues, and further suggesting that the specificity of O6 for the (3 S)Galβ1-4GlcNAc is based on specific interactions with sugar functional groups. The complete binding profile of O6 on the different glycan microarrays can be found in Supplementary Data 1–4.

### O6 binds 3-O-SGal with low micromolar affinity

The affinity and specificity of O6 for the (3 S)Galβ1-4GlcNAc was then analyzed by isothermal titration microcalorimetry (ITC) with several sulfated and nonsulfated glycans from the CFG glycan repository. These glycans included (3 S)Galβ1-4GlcNAc, (3 S)Galβ1-3GlcNAc, (6 S)Galβ1-4GlcNAc, (3 S)Galβ1-4(Fucα1-3)GlcNAc, and Galβ1-4GlcNAc. Two independent experiments were performed using a high and low concentration of the disaccharides. In both experiments, curve-fitting resulted in highly similar K_d_ values of 10 µM (Fig. 1b). Consistent with the glycan microarray data, no binding was detected for the nonsulfated Galβ1-4GlcNAc disaccharide, isomeric β1-3-linked (3 S)Galβ1-3GlcNAc disaccharide, isomeric 6-O-sulfated (6 S)Galβ1-4GlcNAc disaccharide, or (3 S)Galβ1-4(Fucα1-3)GlcNAc trisaccharide (Fig. 1c). The ITC data, which is consistent with the CFG glycan microarray data, confirm that O6 binding requires a 3’-O-sulfate group, free 4’-OH and 6’-OH groups on the Gal, and a free 3-OH group and the 2-N-acetyl group on the GlcNAc residue.

### Structure of O6-glycan complex provides insights into glycan recognition

**Fig. 2:**
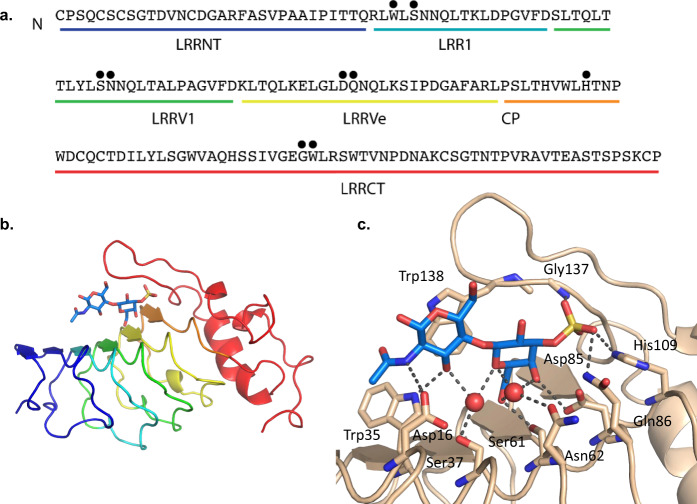
Co-crystal structure of VLR O6 with (3 S)Galβ1-4GlcNAc reveals the interactions and mode of glycan recognition. **a** Amino-acid sequence of VLR O6 with amino acids that come into contact with antigen indicated with a black circle. **b** Co-crystal structure of VLR O6 and ligand. **c** Detailed view of glycan interactions in **b** within the antibody binding pocket.

To better understand the molecular determinants of glycan recognition, we determined crystal structures of unliganded (apo) O6 and a co-crystal structure of O6 in complex with (3 S)Galβ1-4GlcNAc. This O6 VLR is relatively small compared to other characterized VLRBs, containing 178 amino acids (~19.2 kDa) and only a single LRR variable domain (LRRV1) along with the other usual LRR and CP components (LRRNT, LRR1, LRRVe, CP, and LRRCT) (Fig. 2a). Apo O6 has the same overall structure as previously described VLR ectodomains with three-variable LRRs (LRR1, LRRV1, LRRVe) and a long loop in LRRCT, which extends over the concave surface, similar to other sugar-binding LRRs^38,43,44^ and is structurally most similar to the hen egg lysozyme binding VLR^45^. Although the two do not bind the same epitope, they share 70% sequence identity^45^ and the structures align with an RMSD of 0.42 Å for 167 aligned residues (Supplementary Fig. S1A).

The structure of O6 in complex with (3 S)Galβ1-4GlcNAc at 1.9 Å resolution provides a detailed view of sulfated glycan recognition by this VLR (Fig. 2b). The sulfate group of the ligand forms hydrogen bonds with Gln86, His109, and the backbone amide of Gly137 in an identical manner to a phosphate ion in the apo structure (Fig. 2c, Supplementary Fig. S1B). Many hydrogen bonds provide specificity to the recognition of the Gal and GlcNAc moieties. Specifically, Asp85 and Ser61 hydrogen bond to the 4-OH and 6-OH of Gal, respectively. The side-chain NH of Trp35 hydrogen bonds with 3-OH of GlcNAc, while Asp16 hydrogen bonds with the nitrogen of the GlcNAc acetamide group. Two water molecules mediate interactions between Ser37 and Asn62 and GlcNAc and Gal hydroxyl and O groups (Fig. 2c, Supplementary Fig. S2). Trp138 of the C-terminal loop packs against the face of the GlcNAc residue, a feature that has been observed in all glycan-binding VLRs to date^38,44,46^. The binding interactions observed in the crystal structure are consistent with the CFG glycan array and ITC data where VLR O6 makes specific interactions with 3-OH of GlcNAc and the 3-SO_3_, 4-OH, and 6-OH of Gal. The 2-OH of Gal, 6-OH and 1-OH of GlcNAc remain open for substitution or glycosidic linkage. Comparison of the apo and glycan complexes shows no movement of the C-terminal loop and only small changes in residue rotamers (including Trp138), indicating that the VLR is preconfigured for glycan binding (RMSD 0.28 Å for 172 aligned residues) (Supplementary Fig. S1C). The confirmation of the carbohydrate ligand is well supported by electron density and the simulated annealing OMIT map can be found in Supplementary Fig. S3.

### O6 VLRB use in western blotting, TLC-overlay, ELISA, and flow cytometry

**Fig. 3:**
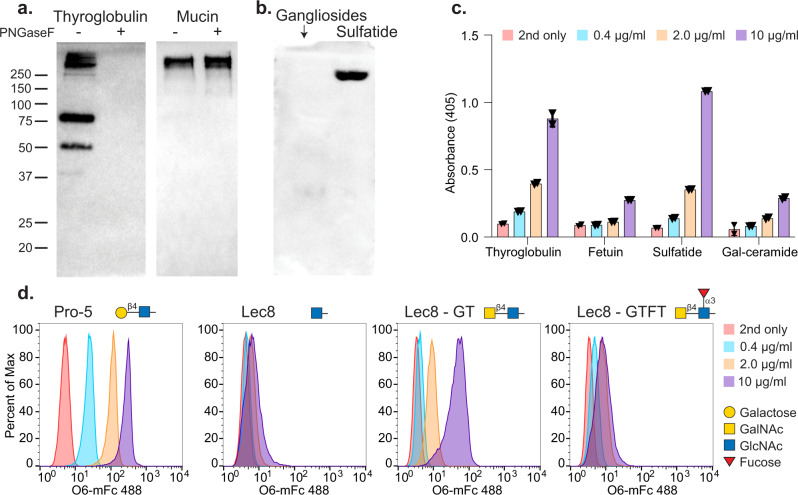
The utility of chimeric VLRs for galactose-3-sulfate detection as demonstrated by western blotting, TLC-overlay, ELISA, and flow cytometry. **a** O6-mFc detects galactose-3-sulfate on the N-glycans of bovine thyroglobulin and O-glycans on human mucins. **b** O6-mFc bound to sulfatide and not porcine gangliosides in TLC-overlay. **c** Validation of **b** and **c** by ELISA, using O6-mFc at 4 concentrations. *n* = 2, each black triangle represents one data point, error bars = ±1 standard deviation. **d** Dose-dependent binding is observed by flow cytometry of Pro-5 CHO cells and not Lec8 mutants. Lec8GT CHO cells are positive; however, addition of α1-3 linked fucose to the LDN motif abrogates binding in Lec8GTFT cells.

We tested whether the recombinant O6-mFc could detect molecules known to carry the (3 S)Galβ1-4GlcNAc determinant by western blotting, TLC-overlay and ELISA (Fig. 3). We chose bovine thyroglobulin and nondiseased human sputum mucin to screen initially, because both are known to carry (3 S)Galβ1-4GlcNAc determinants on N-glycans and O-glycans, respectively^17,47,48^. Both materials were treated with PNGaseF to remove N-glycans, and PNGaseF-treated and untreated samples were blotted onto nitrocellulose and tested for O6 recognition (Fig. 3a). O6-mFc bound to thyroglobulin and binding was lost upon treatment with PNGaseF, demonstrating the O6 epitope is expressed on N-glycans, whereas the binding to mucins and their O-glycans was not affected by treatment with PNGaseF. In blotting on TLC plates containing glycosphingolipids, including porcine gangliosides (glycosphingolipids carrying sialic acids) and sulfatide (3-O-sulfogalactosylceramide), O6-mFc bound to sulfatide, but did not bind gangliosides (Fig. 3b). The results of both the western and TLC blotting were recapitulated by ELISA (Fig. 3c).

For flow cytometry staining, we chose four CHO cell lines that have different glycomic profiles and, while it has been previously reported that GAL3ST1-4 are found within the *Cricetulus griseus* (Chinese hamster) genome^49^, it has not been reported which or if these sulfotransferases are expressed in these specific cell lines. Wild-type Pro-5 cells express complex N-glycans with extended poly-LacNAc (-3Galβ1-4GlcNAcβ1-)_n_ chains (www.functionalglycomics.org), and bind O6-mFc (Fig. 3d). Interestingly, O6-mFc did not bind to Lec8 cells, which is consistent with their lack of galactose on N- and O-glycans, as they are unable to extend the poly-LacNAc glycans due to 97% deficiency in the UDP-galactose transporter^50^, yet are able to express normal levels of glycosaminoglycans^51^. We also screened O6-mFc on the two engineered cell lines that express the *Caenorhabditis elegans* β1,4-N-acetylgalactosaminyltransferase (Lec8GT cells) to generate the LacdiNAc (LDN) antigen (GalNAcβ1-4GlcNAc-R), and Lec8GTFT cells that also express the human α1,3-fucosyltransferase IX to generate the fucosylated LDN structure LDNF (GalNAcβ1-4(Fucα1,3)GlcNAc-R)^52^. O6 binds to the Lec8GT cells expressing the LDN motif; however, the addition of the fucose (LDNF) prevents recognition by the antibody. These data are consistent with and can be explained by the co-crystal structure, which suggests that addition of an N-acetyl group to the 2-OH of Gal should not notably interfere with O6 binding. Since LDN-expressing CHO cells were bound by O6-mFc, the results indicate that the GAL3ST1-4 enzymes in CHO cells are able to generate 3-O-sulfated GalNAc residues using glycans generated by the *C. elegans* β1,4GalNAcT and, as shown above, can be detected with O6, albeit far more weakly than wild-type Pro-5 expressing 3-O-SGal.

### Glycoprotein microarray screening of O6 reveals a wide range of glycoproteins expressing 3-O-SGal

We further explored the ability of O6 to recognize 3-O-SGal epitopes on glycoproteins, as the detection of sulfated glycans on glycoproteins by mass spectrometry can be challenging^24,25^, and its presence may have been overlooked in previous studies. To this end, we generated a glycoprotein microarray to allow high-throughput screening of a range of glycoproteins displaying a variety of glycosylation patterns. For a complete description of how this microarray was generated and validated, see Supplementary Material and Supplementary Data 3–11. The glycoprotein microarray consisted of 35 different glycoproteins (Supplementary Data 3, 5), which were selected based on the following criteria: relative ease of access (defined as commercial availability or ease of accessibility for in-house purification), isolation from natural sources (hence, neoglycoproteins are not included), and the presence of some well-defined glycan structures or determinants on the glycoproteins. Internal positive control glycoproteins for O6 binding on this microarray included bovine and human thyroglobulin as well as human respiratory mucin from both nondiseased (ND) and Cystic Fibrosis (CF) patients. These glycoproteins have well-described glycosylation patterns, some are known to carry terminal (3 S)Galβ1-4GlcNAc determinants^17,47,48,53^, and were bound by O6 in western blot and ELISA formats.

**Fig. 4:**
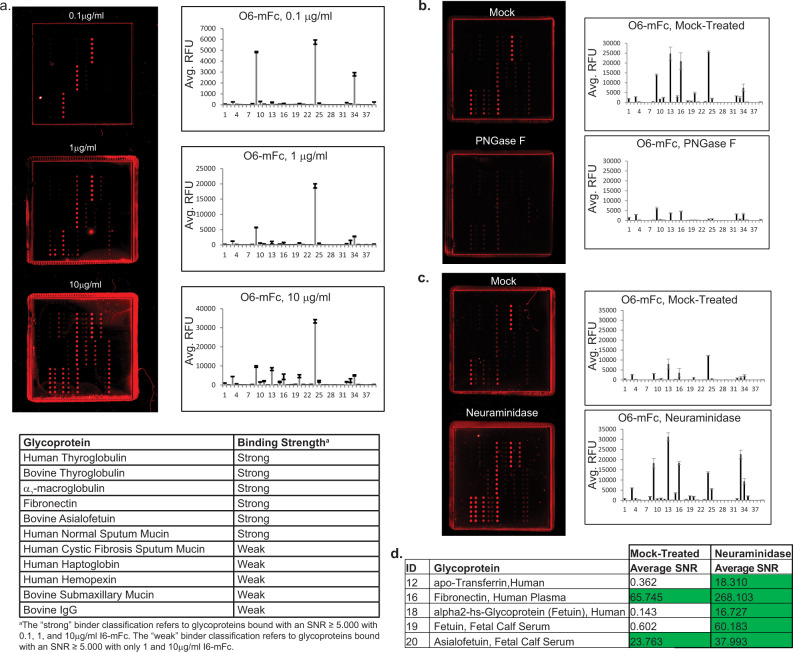
Development of a new glycoprotein microarray reveals many human serum glycoproteins carrying galactose-3-sulfate modification. **a** Binding of O6-mFc on the glycoprotein array at three different concentrations, with the actual slide image shown and the plot of binding, RFU relative fluorescence units, error bars = ±1 SD. **b** PNGaseF treatment of the glycoprotein array eliminates many positive binders, suggesting that galactose-3-sulfate modifications are carried on N-glycans. **c** Treatment of the glycoprotein array with neuraminidase reveals many more positive binders and are summarized in the table **d**) below the chart.

We probed the glycoprotein array with multiple concentrations of O6-mFc and observed dose-dependent binding to several glycoproteins (Fig. 4a, Supplementary Data 3). The positive control glycoproteins bovine and human thyroglobulins, normal (ND) sputum mucin, and CF mucin were bound by O6-mFc as predicted, confirming the sensitivity and validity of this platform. Our screening identified several other glycoproteins, however, that have not been reported to carry 3-O-SGal, including haptoglobin and bovine fetuin. We were especially intrigued by the observation that O6-mFc bound to asialofetuin, but not normally sialylated fetuin, suggesting that terminal sialic acid residues may mask O6 recognition of 3-O-SGal. Although the glycosylation of fetuin has been studied frequently^54^, it has not been reported to contain 3-O-SGal.

We then treated the array with PNGaseF to further confirm glycan-specific binding of O6 to these glycoproteins and determine if the sulfated glycans were on N- or O-glycans. As it was unclear if PNGaseF would be effective at removing the N-glycans from proteins printed on a nitrocellulose platform, we used binding of the plant lectin concanavalin A (ConA), which recognizes high mannose, hybrid and biantennary complex N-glycans, as a positive control. Treatment with PNGaseF reduced ConA binding, from ~3-fold to complete elimination of binding compared to a mock-treated subarray (Supplementary Data 4). This variation most likely reflects differences in the ability of PNGaseF to access glycans on the immobilized glycoproteins. PNGaseF treatment of the array also decreased binding of O6-mFc to human and bovine thyroglobulin, α_2_-macroglobulin, and fibronectin (Fig. 4b, Supplementary Data 4). The exception was binding to normal human sputum mucin (ID #4), a result that is consistent with the western blot analysis and previous reports that mucins have O-glycans^47,53^, some containing 3-O-SGal^48^, and thus not susceptible to PNGaseF.

The observation that O6 binds to asialofetuin but not fetuin suggested that there some glycans on this protein may be both sialylated and sulfated, an observation that has not been previously reported. To investigate if this pattern was observed more broadly on other proteins, we treated the array with neuraminidase A, which cleaves α2-3, α2-6, and α2-8 terminal sialic acid. The efficiency of the neuraminidase treatment was validated with *Sambucus nigra* lectin (SNA), which binds sialylated glycans with α2-6 linkage (Supplementary Data 4). After neuraminidase treatment, binding of O6 to bovine fetuin was equivalent to bovine asialofetuin, which acted as another control for the neuraminidase. Interestingly, treatment with neuraminidase increased binding of O6-mFc to several glycoproteins, including α_2_-macroglobulin, sputum mucins, fibronectin, and haptoglobin (Fig. 4c, d). Additionally, other glycoproteins that did not initially bind were bound by O6-mFc after neuraminidase treatment, including transferrin, vitronectin, human fetuin, and bovine fetuin. These results demonstrate that the 3-O-SGal epitope is present in multiple glycoproteins not previously shown to contain this epitope.

### Identification of 3-O-SGal on sialylated N-glycans of human serum glycoproteins and bovine fetuin

**Fig. 5:**
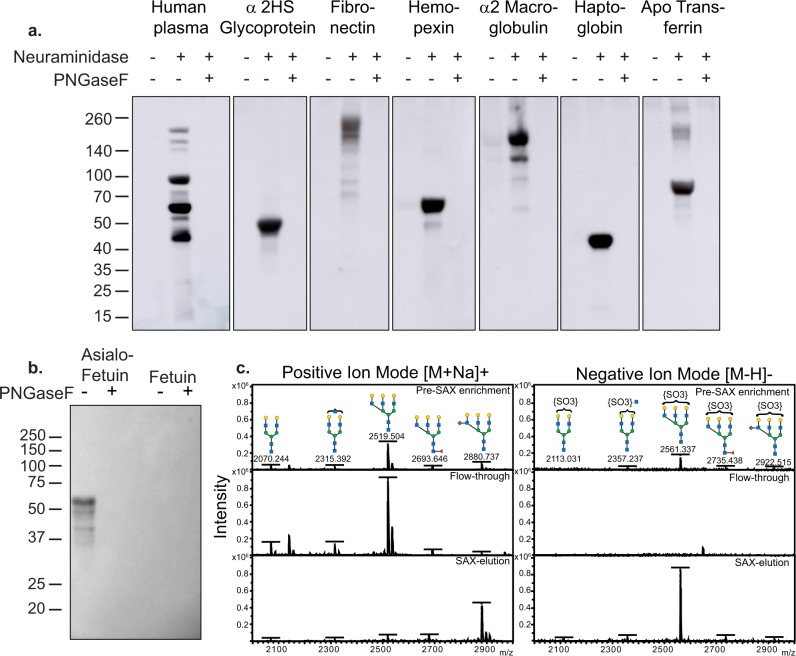
Analysis of human plasma glycoproteins and bovine fetuin. **a** Western blot of normal human plasma and purified human serum glycoproteins reveal galactose-3-sulfate modification present on sialylated N-glycans. **b** Western blot of fetuin and asialofetuin identify the galactose-3-sulfate modification is present on N-glycans. **c** MALDI-TOF analysis in positive and negative ion mode of PNGaseF released N-glycans. The predominant peak containing the galactose-3-sulfate modification on a triantennary N-glycan enriched in the SAX-eluted fraction (negative mode).

These results suggest that a number of human serum glycoproteins may express terminal glycan modification by both sialic acid and 3-O-SGal, and that sialic acid may mask binding by O6-mFc. To further investigate this phenomenon, we chose six of the glycoproteins printed on the array, as well as albumin/IgG depleted human plasma, to screen via western blot using O6-mFc (Fig. 5a). All of the samples were treated with neuraminidase or neuraminidase plus PNGaseF prior to the assay. Consistent with the glycoprotein array results, O6 bound to glycoproteins after neuraminidase treatment, and the addition of PNGaseF abrogated all recognition, thus indicating the 3-O-SGal epitope is on N-glycans. Multiple bands were seen in the human plasma sample following neuraminidase treatment, including those with sizes corresponding to fibronectin, alpha-2 macroglobulin, and apo-transferrin, while human serum fetuin, hemopexin, and haptoglobin show a large, singular band positive for 3-O-SGal. These results demonstrate that N-glycans on many human plasma glycoproteins carry sialic acid and 3-O-SGal modifications, and that removal of sialic acid unmasks the 3-O-SGal for binding by O6.

The discovery that some human serum glycoproteins express the 3-O-SGal modification is noteworthy, as the N-glycosylation profiles of these proteins have been well-documented and, in some instances, have been reported as correlates of disease^55–58^. It was particularly surprising that bovine asialofetuin was recognized by O6 (Fig. 5b), as it has been a standard for glycan analysis for decades^54^ and it has not been reported previously to have the 3-O-SGal modification. To this end, fetuin was treated with PNGaseF, and released N-glycans were subsequently passed through a strong anion exchange column (SAX)^59^. The glycans were permethylated according to the methodology developed by Khoo and Yu^24^ and analyzed at each stage of the enrichment (Pre-SAX, Flow-through, and SAX elution) by MALDI-TOF in both positive and negative ion mode (Fig. 5c).

As the N-glycans have been very well-characterized from fetuin, the glycans analyzed in positive ion mode (Fig. 5c, left panel) acted as a useful positive control for the efficacy of the SAX enrichment itself. As established previously, N-glycans on asialofetuin are predominately triantennary; however, some biantennary structures are also present as indicated. The flow-through, or unbound glycans to the SAX, were all neutral as expected, and the SAX-eluted fraction contained only a single structure, a triantennary glycan with a single sialic acid. This is consistent with the prior report of fetuin containing a sialic acid linked to a penultimate GlcNAc residue^60^, which is resistant to neuraminidase treatment. Thus, this sialic acid-containing glycan was retained on the SAX column due to its negative charge, but was detected in positive ion mode due to the permethylation of the sample.

SAX enrichment revealed a predominant peak (*m/z* = 2561.337) consistent with a sulfate moiety on a glycan with 6 hexoses and 5 hexNAcs (6H5N). This structure was only detected in negative ion mode, and increased in intensity by four fold in the SAX-eluted sample (Fig. 5c, right panel). The data are consistent with this structure representing a triantennary glycan; however, the specific branch that contains the sulfate group could not be determined with this type of analysis. Thus, a small proportion of N-glycans of bovine fetuin express the sulfate modification recognized by O6, but are sialylated and masked in a manner that prevents recognition by O6 without removal of the sialic acid.

### Immunohistochemical analysis of normal human tissues with O6

**Fig. 6:**
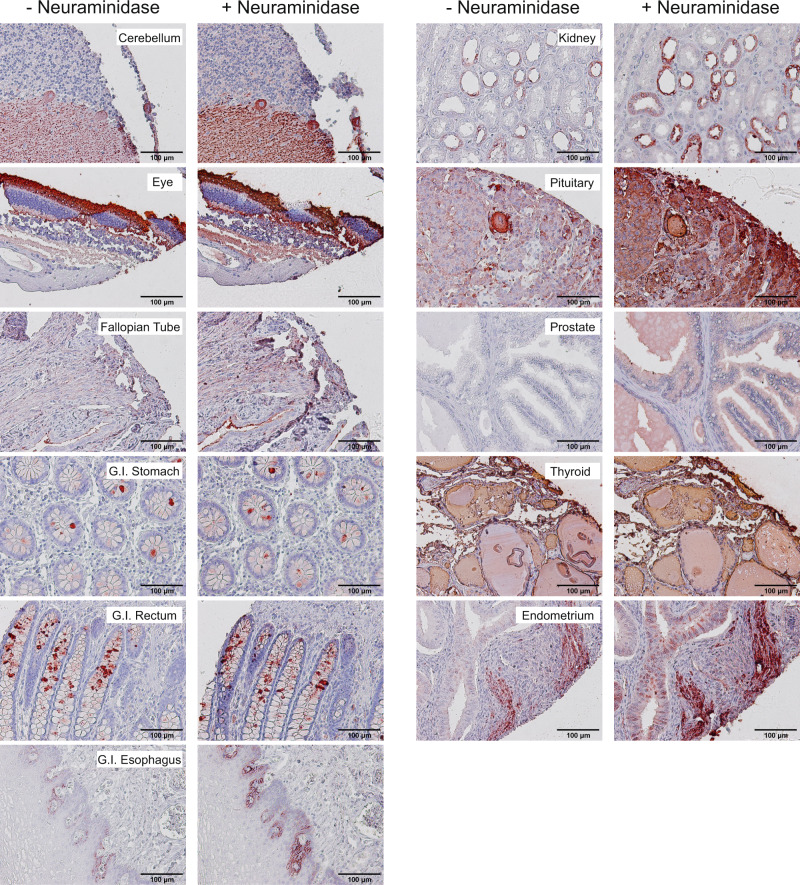
IHC of healthy human tissues with O6. Several tissues stained positive with O6, including cerebellum, eye, fallopian tube, G.I.-stomach, G.I.-rectum, G.I.-esophagus, kidney, pituitary, prostate, thyroid, and endometrium. The left panels depict the staining profile prior to neuraminidase treatment and the right panels are after (+) neuraminidase. Scale bars are noted on each image.

To further probe the overall expression of 3-O-SGal, we screened a broad selection of human tissues for O6 reactivity using paraffin-embedded tissue microarrays. Due to the processing and removal of the paraffin, which removes glycosphingolipids, the observed staining can be attributed only to glycoproteins. We observed O6 staining to sections from cerebellum, eye, Fallopian tube, stomach, rectum, esophagus, kidney, pituitary, prostate, thyroid, and endometrium (Fig. 6). Nonstained or very weakly stained tissues included the adrenal gland, bladder, bones, cerebral cortex, ovary, cervix, placenta, small intestine, colon, heart, liver, lung, testis, pancreas, skin, spinal cord, spleen, striated muscle, thymus, and tonsils (Supplementary Fig. S4). Importantly, the observed staining had distinct patterns in different tissues. For example, O6 staining of goblet cells was observed primarily in the lower to middle section of the crypts, comparable to the expression profile previously reported for GAL3ST-3 in the gastrointestinal system^61^. However, some individual goblet cells showed strong positive staining whereas others were negative. Another distinct pattern of O6 staining was observed in the kidney, where only cells lining the renal tubules showed staining, and is consistent with the previously reported expression profile of GAL3ST-3 (Supplementary Table S2). Given our observations on O6 binding to the glycoprotein array, it might be predicted that treatment of fixed tissue with neuraminidase would unmask the presence of 3-O-SGal. Indeed, we observed increased binding of O6 in several tissues treated with neuraminidase, including kidney, pituitary, and prostate (Supplementary Figs. S4, S5). These results reveal the widespread occurrence of 3-O-SGal modifications and masking by sialic acid in tissues that are known to express GAL3ST-2/3. Interestingly, there were several carcinoma and diseased specimens on the human tissue array, including breast, melanoma, thymus cancer as well as HBV^+^ cirrhotic liver tissue. After neuraminidase treatment, liver and melanoma tissues were O6 positive while the breast carcinoma tissue remained negative and thymoma was positive in both treated and untreated tissues (Supplementary Fig. S6). While not conclusive, these data suggest that O6 has the potential to be a cancer specific biomarker, and further exploration of these results should be pursued with more samples that include relevant clinical data.

## Discussion

We have explored the expression of the 3-O-SGal epitope using a novel monoclonal antibody O6 that specifically binds to glycans carrying the 3-O-SGal modifications. Our approach is based on identifying a variable lymphocyte receptor (VLR) using lymphocytes from lampreys immunized with human type O erythrocytes. The screening technologies used here employed a YSD library to capture the desired VLR-expressing yeast on glycan microarrays^39^. We expressed the VLR O6 as a recombinant O6-mFc protein and demonstrated its usefulness as the first reagent to document the expression of 3-O-SGal in different human tissues and glycoproteins. While our microarray results indicate that O6 can bind both 3-O-SGal and 3-O-SGalNAc, the latter has not yet been described in human glycoproteins, and thus we focused on the 3-O-SGal epitope recognized by O6.

We demonstrated that O6 recognizes 3-O-SGal using multiple approaches, including glycan microarray screening, ligand binding by ITC, and a crystal structure of a 3-O-SGal containing glycan with O6. To aid in this we also created a glycoprotein microarray and identified a number of glycoproteins not previously known to carry 3-O-SGal, including human serum glycoproteins and bovine glycoprotein fetuin. Thus, 3-O-SGal occurs more widely than appreciated previously and O6 represents a novel reagent to identify expression of this determinant and explore its function in human and other animal cells.

We observed that O6-mFc recognizes 3-O-SGal with relatively high affinity as quantified by ITC. The binding interactions observed in the crystal structure were consistent with the CFG glycan array data, ITC, and glycoprotein arrays. Most notably, the crystal structure data explains the necessity of treating the samples with neuraminidase, which would remove the sialic acid from the 6th position on the galactose, where masking might occur. O6 makes specific interactions with 3-OH of GlcNAc and the 3-SO_3_, 4-OH, and 6-OH of Gal whereas the 2-OH of Gal, and 6-OH and 1-OH of GlcNAc remain available for substitution or glycosidic linkage. There are two notable exceptions: the O6 recognition of 6 S(3 S)Galβ1-4(6 S)GlcNAc (CFG Glycan ID #22) and (3 S)Galβ1-4(Fucα1-3)(6 S)GlcNAc (CFG Glycan ID #219). Neither of these glycans would be expected to bind based on the co-crystal structure. We surmise that the observed binding is a result of avidity afforded by the combination of dense presentation of glycans on the array and the bivalent presentation of VLR-binding sites in the Fc-tagged construct used for screening.

Interestingly, O6 binds to sulfatide (3-O-sulfogalactosylceramide) (Fig. 3b), indicating that the 3-O-SGal alone is sufficient for O6 binding, but binding to the simple 3-O-SGal alone on the CFG glycan microarray was not observed (Fig. 1). This is not surprising, as the 3-O-SGal alone directly with a spacer/linker is small in size and adjacency to the slide matrix may affect its presentation to the antibody. Regardless, sulfatide and perhaps other glycolipids represents another potential ligand for O6. Given that red blood cells were used as the immunogen for generating O6, and the presence of low but detectable levels of sulfatide on human red blood cells (~0.5 mg/kg cell mass)^62^, this raises the possibility that sulfatide might have been the antigen leading to this 3-O-SGal-specific VLRB. However, it is also possible that red blood cell glycoproteins could be expressing 3-O-SGal, but this has yet to be explored.

Following the historical discovery of 3-O-SGal in sulfatide^11^, and as deduced by Yamakawa et al. in 1962^63^, it was subsequently shown that the 3-O-SGal modification is present in human thyroglobulin by Spiro and Bhoyroo in 1988, and in other glycoproteins by others^12,17–20,48,64–68^. The 3-O-SGal modification has been found within the motifs sulfo-3Galβ1- 4(FucR1-3)GlcNAc-R (3’-sulfo-Le^x^) and sulfo-3Galβ1-3(FucR1- 4)GlcNAc-R (3’-sulfo-Le^a^), which are important in various cellular functions and in disorders including cancer and inflammation. For example, the 3-O-SGal modification is important in regulating the function of integrin subunit αV^69^, and in promoting interactions of cells to E-selectin^66,70^. We anticipate the O6 will be invaluable in future studies to identify glycoprotein glycans carrying the 3-O-SGal epitope.

There are some physiologically important endogenous glycan-binding proteins that appear to recognize 3-O-SGal in glycoprotein glycans, but this needs further exploration. For example, the macrophage mannose receptor^71^ and galectin-4^72^ can bind glycans with 3-O-SGal modifications, and we noted previously that two other galectin family members, galectin-2 and -3, exhibit enhanced recognition of glycans with terminal 3-O-SGal residues, whereas either 4- or 6-O-sulfation blocked binding^73^. However, the overall expression and biological functions of 3-O-SGal have been enigmatic, as no reagents have been available to specifically identify this modification. Interestingly, complications in understanding expression of 3-O-SGal have also arisen, as it was shown that the plant lectin MAL-I, which is typically used to identify sialylated glycans in the sequence Neu5Acα2-3 Galβ4GlcNAc-R, can also bind 3-O-SGal in the sequence 3SGalβ1-4GlcNAc-R^27^. With the availability of O6, it will now be possible to specifically identify these sulfate modifications in glycoconjugates.

**Fig. 7:**
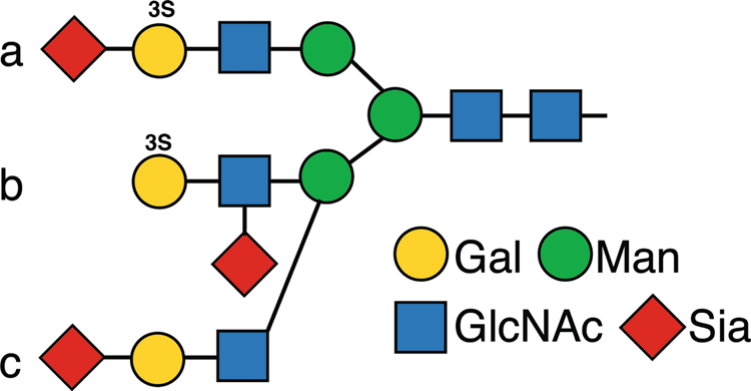
Theoretical combinations of terminal 3-O-sulfated galactose and sialic acid. Potential relationships of **a**, **b**, and **c** of 3-O-SGal and sialylation of N-glycans, as discussed in the text.

The masking of the 3-O-SGal epitope by sialic acid has not been noted previously. The unmasking by neuraminidase treatment suggests that sialic acid is linked near to the 3-O-SGal epitope; possibly on (a) the galactose itself, e.g., 6-O-sialic acid linkage, (b) the penultimate GlcNAc residue, or (c) a closely associated branch of the glycan (see Fig. 7). These possibilities may not be exclusive and could all be relevant to unmasking. Interestingly, such a modification of sulfated and sialylated galactose as in (a) of Fig. 7 has been reported in N-glycans of human plasma glycoproteins, although the linkages were not well defined^74^.

Such a masking role of sialic acid has been generally noted in other cases, however, as it may block recognition of underlying determinants^75–77^. Such unmasking might be physiological relevant, as the human genome contains several genes that encode neuraminidases, including *Neu1-4*. Thus, unmasking of the 3-O-SGal could arise naturally if endogenous neuraminidases in human cells can facilitate removal of sialic acid on surface or secreted glycoproteins. For example, one of the endogenous neuraminidase genes *Neu3* encodes a surface and secreted neuraminidase that desialylates plasma glycoproteins in some disease states^78^. Unmasking could also occur in sepsis, as invasive microbes, e.g., *Streptococcus pneumoniae*, express sialidases active on host glycoconjugates^79,80^. It is also possible that synthesis of 3-O-SGal can be competitive with other modifications of the galactose. For example, studies using CHO cells demonstrated that introduction of GP3ST blocked α3-sialylation of N-glycans^81^, and introduction into pig endothelial cells blocked α3-galactosylation of galactose^82^. Finally, the presence of 3-O-SGal might be beneficial in blocking microbial attachments, as observed where mucin sulfation protects intestinal cell walls in chicks against adhesion and invasion by *Campylobacter jejuni*^83^.

The unexpected widespread occurrence of the 3-O-SGal modification was further revealed upon screening on a newly generated glycoprotein microarray that contained over 35 different glycoproteins, many of which had relatively well-known glycosylation signatures and glycan sequences. Predictably, human thyroglobulin, known to carry high levels of terminal 3-O-SGal, showed the highest signal of interactions with O6, whereas bovine thyroglobulin was lower. This result is consistent with prior reports that, despite the 77% sequence identity shared between the two proteins, the degree of sulfation is far lower in bovine thyroglobulin^17^. Well-studied glycoproteins that were not known to express 3-O-SGal, such as bovine asialofetuin and human α_2_ macroglobulin were also bound by O6, while other glycoproteins such as human transferrin were only bound when treated with neuraminidase. Our observation of 3-O-SGal on multiple human serum proteins and masking by sialic acid is consistent with the evidence that the enzyme primarily responsible for 3-O-SGal modifications of N-glycans and O-glycans, galactose-3-O-sulfotransferase-2 (Gal3ST-2), is expressed to some extent in the human liver, where many non-immunoglobulin related serum glycoproteins are synthesized^61^. Therefore, 3-O-SGal may be a common, yet previously overlooked occurrence among human serum glycoproteins.

The glycoprotein microarray was generated for these studies from naturally occurring, highly purified and commercially available glycoproteins. Although only 35 glycoproteins were used on this microarray, in total these glycoproteins with their varied glycan structures may display hundreds of different N- and O-glycan determinants, and thereby may represent a largely untapped set of glycan determinants found in nature but lacking in standard glycan microarrays. Additionally, the glycoprotein microarray provides natural presentation of glycans, different from the defined glycan microarrays where chemical linkers are used for glycan immobilization to slides^84–86^. Further information and discussion of this glycoprotein microarray can be found in Supplemental Materials.

## Conclusion

The availability of the 3-O-SGal-specific VLRB, O6, will be a valuable biomedical research tool. Importantly, O6 can recognize glycoproteins that may contain a very low percentage of sulfated glycans, such as fetuin and transferrin. Moreover, screening of O6 on the glycoprotein microarray revealed that the presence of the 3-O-SGal epitope may be more common on glycoproteins than previously believed. Future studies will be aimed at utilizing O6 to understand the biological expression and function of 3-O-SGal epitopes in cells and tissues as well as developing other VLRBs that target other sulfated glycan determinants for which no antibodies are available.

## Methods

### Antigen preparation

Peripheral blood samples were collected in heparin coated collection tubes from healthy volunteers at the Vaccine Center at Emory University, Atlanta, GA under an approved IRB for an unrelated study and unused, coded samples were provided. Total leukocytes, erythrocytes, and plasma were isolated using lymphocyte separation medium (Corning). Erythrocytes were stored in Alsever’s solution (NaCl 4.2 g/L, sodium citrate•2H_2_O 8 g/L, citric acid•H_2_O 0.5 g/L, D-glucose 20.5 g/L) at 4 °C for a maximum of 1 month.

### Animals, immunization, plasma, and leukocyte isolation

*Petromyzon marinus* larvae (8–15 cm in length, ~2–4 years in age) were collected from tributaries to Lake Michigan (Lamprey Services, Ludington, MI) and housed in sand-lined aquarium tanks at 20 °C in the animal facility. Lamprey husbandry and immunizations protocols were approved by the institutional animal care and use committees (IACUC) at Emory University and were strictly adhered to during experiments. Animals (*n* = 3) were anesthetized with MS-222 (0.1 g/L) and given intracoelomic injections (*n* = 3) at 2-week intervals with human blood type O erythrocytes. Two weeks after the final injection, the animals were sacrificed with MS-222 (1 g/L) and exsanguinated. Total lamprey blood was collected into 0.67 × PBS with 30 mM EDTA and layered onto 55% percoll and centrifuged for 20 min at 400 × *g*. Total leukocytes were collected, washed three times in 0.67 × PBS and stored in RNAlater at −20 °C. Lamprey plasma was collected and stored at 4 °C and examined for positive VLRB titers. Serial dilutions of lamprey plasma were combined with 10^6^ washed human erythrocytes into a v-bottom microwell plate. Samples were incubated for 1 h at room temperature and assayed for agglutination by tilting the plate for 2 min. The individual lamprey with the highest reciprocal hemagglutination titer (1:10,000) was used for all further analysis and antibody isolation.

### Discovery and expression of VLRB clone O6

VLRB clone O6 was discovered from a yeast surface display (YSD) library generated from lamprey immunized with human type O erythrocytes. Briefly, after the immunization series was completed, VLRB-specific cDNA libraries were PCR amplified (2 μg total) from total leukocyte cDNA and electroporated into the EBY100 strain of *Saccharomyces cerevisiae* with 1 μg of the pCT-BDNF-ESO expression vector^40^. YSD libraries were immediately transferred to SD-CAA liquid media for growth and recovery, and the size of the YSD library was determined by plating a serial dilution of the YSD onto solid SD-CAA agar plates (~5 × 10^6^ transformants). The YSD was then grown for several days in SD-CAA selection media at a concentration of 1 × 10^6^ yeast/mL, and archived at −80 °C as the unenriched library. To enrich for erythrocyte-specific VLRB clones, the YSD libraries were grown in liquid SD-CAA media overnight at 30 °C, shaking at 260 RPM and then diluted 1:5 in fresh media and grown for an additional 3 h at 30 °C, shaking at 260 RPM^41^. The YSD library was then transferred into SG-CAA induction media and grown for 18–24 h at 20 °C, shaking at 260 RPM. To determine if the induction of VLRB expression was successful, the YSD library was labeled with an anti-Myc-488 monoclonal antibody and monitored by flow cytometry.

The YSD library was then enriched for human type O erythrocyte VLRB clones by labeling cell surface proteins on red blood cells with biotin (EZ-link Sulfo-NHS-LC-Biotin, ThermoFisher Pierce) and incubating with the induced library for 1 h at room temperature on rotation. The yeast bound to the labeled erythrocytes were then captured on streptavidin-labeled magnetic beads (MACS sorting) and eluted directly into SD-CAA media and grown overnight. This library was further enriched by FACS using the same approach. The success of the MACS and FACS enrichment was monitored by flow cytometry using the anti-Myc 488 and SA-PE secondary reagents. After one round of MACS and FACS enrichment, the YSD library was incubated overnight on the CFG microarray, gently shaking at 4 °C in PBS with 0.05% tween and 1% BSA. Slides were washed with PBS-T (0.05% tween) to remove unbound yeast from the array. Yeast clones that were bound to the array were transferred to solid media, sequenced and cloned into a modified pCDH mammalian expression vector containing the IgG2a mouse Fc constant region and a 6 × HIS tag. The VLRB O6-mFc chimeric protein was produced by transiently transfecting the expression vector into 293 F cells using polyethylenimine (PEI) (1:3 of DNA to PEI) and 2.2 mM VPA. The protein was then purified from the media according to the standard methods recommended from the manufacturer of HisPur Cobalt Resin column (ThermoFisher Scientific).

### Generation of a glycoprotein microarray

The Glycoprotein Microarray was generated using a panel of 35 glycoproteins described in Supplementary Data 5. The glycoprotein microarray also included bovine serum albumin (BSA) as a nonspecific protein interaction control, biotinylated BSA as a positive control for Streptavidin binding, and Streptavidin-Cy5 (Molecular Probes) as a landing light for microarray alignment. Glycoproteins, most of which were supplied as a dry powder, were suspended in phosphate-buffered saline (PBS; 6.7 mM potassium phosphate pH 7.5, 0.15 M sodium chloride) at a stock concentration of 0.5–20 mg/mL and then 0.2 µm filtered. Human IgM and human placental laminin, which were supplied as solutions, were aliquoted and filtered prior to use. Aliquots of the filtered stock solutions were stored at −80 °C, except IgM, which was stored at 4 °C.

Immediately prior to printing, a single aliquot of each of the glycoprotein stock solutions was thawed on ice. The glycoproteins were each diluted to a final concentration of 0.1 mg/mL in PBS, and 25 µl of each 0.1 mg/mL glycoprotein sample was transferred to a 384-well polypropylene plate (source plate). As controls, 0.1 mg/mL bovine serum albumin (control for nonspecific protein binding), 10 µg/mL biotinylated BSA (technical positive control for Streptavidin probe activity), and 1 µg/mL Streptavidin-Cy5 (landing light and alignment control for the Standard Red fluorescence channel) were also prepared, and 25 µL of these three controls were added to the source plate. The source plate was centrifuged at 1000 × *g* for 3 min to remove air bubbles.

Microarray printing was performed using a sciFLEXARRAYER S11 microarray printer (Scienion). SuperNOVA nitrocellulose-coated slides in a 16-subarray format (Grace-Bio) were preincubated at 60% humidity and room temperature prior to printing. The source plate was kept at 9 °C before and during the printing process to minimize protein aggregation and precipitation. Printing was performed with Nozzle Type 3 (PDC 90) pins at room temperature and 60% humidity. After printing, the slides were incubated for an additional 1 hr on the dock at room temperature and 60% humidity. Afterwards, the printed slides were incubated in a box overnight at 4 °C.

After overnight incubation, all slides were scanned with a GenePix 4300 A scanner (Molecular Devices) with 635 nm laser and Standard Red filter to detect salts and thus the printing spots. The slides were blocked with Super G Plus Preservative solution (Grace-Bio) according to the manufacturer’s recommended protocol. The blocked slides were stored at −20 °C until use.

### O6 screening on CFG glycan microarray and glycoprotein microarray

Lamprey plasma was diluted (1:10) into TSM binding buffer and incubated on a glycan microarray slide containing 610 unique glycan structures (version 5.0)^87^. Detection of anti-glycan specific VLRBs present within the lamprey plasma was accomplished with 4C4, an anti-VLRB mouse IgG mAb^29^. The chimeric VLRB-mFc O6 was bound to the CFG array at five-fold concentration intervals (2 μg/mL, 10 μg/mL and 50 μg/mL) to confirm dose-dependence. Alexa-Fluor 488 labeled goat anti-mouse IgG mAb (Molecular Probes) was used for detection of both lamprey plasma and the O6-mFc recombinant fusion protein.

The Glycoprotein Microarray v3 slides were removed from −20 °C and dried in a vacuum desiccator for at least 20 min. The slides were fitted with a ProPlate 16-well chamber (Grace-Bio). The subarrays of interest were first incubated with 200 µl of TSM wash buffer (TSMWB; 20 mM Tris pH 7.4, 150 mM NaCl, 2 mM MgCl_2_, 2 mM CaCl_2_, 0.05% v/v Tween-20) for at least 30 min. All incubation steps were performed at room temperature on an orbital shaker (~50 RPM shaking). O6-mFc was diluted to the desired concentration using TSMWB supplemented with 10% w/v bovine serum albumin (BSA; Boval) and 0.1× Super G Blocking Buffer (Grace-Bio). Seventy microliters of diluted O6-mFc solution was applied to the subarray(s). The chambers were sealed with an adhesive strip and the slide incubated for 1 h. The subarrays were washed four times for 5 min each with 200 µl of the following order of solutions: TSMWB, Super G Blocking buffer, TSMWB, and TSMWB. The subarrays were then briefly washed four times with TSM (20 mM Tris pH 7.4, 150 mM NaCl, 2 mM MgCl_2_, 2 mM CaCl_2_). The bound O6-mFc was detected by incubation of the subarrays for 1 h in the dark with Alexa-Fluor 633-conjugated goat anti-mouse IgG (Molecular Probes) that was diluted to 5 µg/mL final in TSMWB supplemented with 1% w/v BSA. The subarrays were then washed with four 5-min washes with TSMWB followed by four quick washes with TSM and finally four quick washes with Milli-Q-filtered water. The subarrays were air dried in the dark for at least 10 min prior to slide scanning with a GenePix 4300 A microarray scanner (Molecular Devices) with the 635 nm laser and Standard Red filter setting.

### Glycosidase treatment of the glycoprotein microarray

Glycosidase treatments were performed immediately following subarray rehydration. For neuraminidase treatment, the rehydrated subarrays were washed three times for 5 min with neuraminidase buffer (50 mM sodium acetate pH 5.5, 4 mM CaCl_2_) at room temperature on an orbital shaker. *Arthrobacter ureafaciens* neuraminidase (Roche) was diluted to 1.5 U/mL final in neuraminidase buffer, and 70 µL of the 1.5 U/mL neuraminidase was applied to the subarray. As a mock-treatment control, 70 µl of neuraminidase buffer alone was added to another subarray. The chambers were then sealed with an adhesive strip, and the slide was incubated for 3 h at room temperature on an orbital shaker. The subarrays were washed four times for 5 min each with neuraminidase buffer supplemented with 0.05% v/v Tween-20 at room temperature on an orbital shaker. The subarrays were then briefly washed four times with neuraminidase buffer and then four times with Milli-Q filtered water, and immediately screened with either SNA (as a control for sialic acid removal) or O6-mFc.

For PNGaseF treatment, the rehydrated subarrays were washed three times for 5 min with 1× G7 buffer (New England Biolabs) at room temperature on an orbital shaker. PNGaseF (500U/µL, New England Biolabs) was diluted 1:10 to 50U/µL final in G7 buffer. PNGaseF (70 µL of 50U/µL) was then added to the subarray(s), while 70 µL 1× G7 buffer was added to a separate subarray to serve as a mock-treatment control. The slides were sealed with an adhesive strip and incubated at 37 °C in a humidified incubator for 24 h. The subarrays were washed four times for 5 min with TSMWB on an orbital shaker at room temperature. The subarrays were briefly washed four times with TSM followed by four brief washes with Milli-Q-filtered water, and immediately screened with either ConA (as a control for N-glycan removal efficiency) or O6-mFc.

### VLR 06 ectodomain for ITC and crystallization experiments

The VLR 06 ectodomain was synthesized with an N-terminal gp67 secretion signal and hexahistidine tag and cloned into phCMV 3. Plasmids were purified with a Macherey-Nagel Maxi Prep kit. Proteins were expressed by transient transfection in HEK293 cells using standard methods. For purification, 5 mL Ni-NTA resin was added directly to clarified culture supernatant and incubated overnight. The resin was washed with 100 mL wash buffer (50 mM Tris pH 8.0, 300 mM NaCl, 15 mM imidazole) followed by 15 mL elution buffer (50 mM Tris pH 8.0, 300 mM NaCl, 300 mM imidazole). The sample was concentrated and further purified by size exclusion chromatography on a Superdex 75 16/60 column in 25 mM Tris pH 8.0, 150 mM NaCl.

### Isothermal titration microcalorimetry

An Auto-iTC200 instrument (GE Healthcare) was used to perform isothermal titration calorimetry (ITC). O6 recombinant protein was purified, and lyophilized glycans were resuspended, in a buffer containing 25 mM HEPES pH 7.5 and 250 mM sodium chloride. Glycans were obtained from the CFG or purchased from Glycotech or Sigma and contained an Sp-biotin linker (CH_2_CH_2_NH-biotin). Glycans were placed in the syringe at a concentration of 1–5 mM, and O6 was placed in the cell at a concentration of 54–67.7 μM. The O6 concentration was determined by UV absorbance at 280 nm using calculated extinction coefficients. Experiments were carried out at 25 °C and consisted of 16 injections of 2.45 μL, with injection duration of 1 s, injection interval of 180 s, and reference power of 5 μcal. Fitting of integrated titration peaks was performed with Origin 7.0 software using a single-site binding model to measure the affinity constant (K_a_) value. The K_d_ value was then calculated as the inverse of the K_a_.

### Crystallization and structure determination

Crystallization screening was carried out using our in-house Rigaku CrystalMation system. No crystals and very little precipitation were observed in crystallization trials with wild-type O6 ectodomain, even at concentrations as high as 50 mg/mL. The sequence of the 06 ectodomain was submitted to the surface entropy reduction prediction (SERp) server for analysis. Three sites were suggested for mutation: K18A, K80A/E81A, and Q88A/K90A. All three variants were made using standard site-directed mutagenesis protocols and expressed and purified in the same manner as the WT protein. However, the purification yield was ~10-fold lower than the WT protein. Crystals of apo O6 K18A at 10 mg/mL were grown with a well solution of 1.2 M NaH_2_PO_4_, 0.8 M K_2_HPO_4_, 0.1 M glycine pH 10.5, 0.2 M Li_2_SO_4_ using the sitting drop vapor diffusion method and directly flash cooled in liquid N_2_. Data were collected at APS 23 ID-D (GM/CA @ APS) and processed with HKL2000. Phaser was used for molecular replacement within Phenix^88^ using PDB ID: 1RVX as a search model. For the disaccharide complex, thrombin-cleaved O6 K18A at 10 mg/mL with 2 mM 3-HSO_3_-LacNAc was mixed at a 1:1 ratio with well solution containing 2.4 M AmSO_4_, 0.1 M Bicine, and 20% glycerol. Crystals were directly flash cooled in liquid N_2_. Data were collected at SSRL Beam line 9-2 and processed with XDS. Molecular replacement was carried out in Phenix using the O6 apo structure as a model. The ligand was built using the monosaccharides NAG and SGA. Automatic linking during refinement in Phenix formed the glycosidic bond^89^. For both structures, the model was refined through iterative rounds of model building in COOT^90^ and refinement in Phenix. TLS groups were automatically identified by Phenix. Waters were manually added and edited in COOT. The final model was assessed with quality metrics within the Phenix refine interface, which utilizes MolProbity^91^. The geometry of the carbohydrate ligand was validated with Privateer. Data collection and refinement statistics are listed in Supplementary Table S3.

### O6-mFc blotting, TLC, and ELISA with glycoproteins and glycosphingolipids

All of the glycoproteins used for the western blot, and their commercial sources are listed in Supplementary Data 5. Five micrograms of each individual protein were loaded onto a SDS-PAGE gel in 1× Laemmli buffer, transferred to nitrocellulose and probed with the O6-mFc recombinant protein using the following conditions: blocking in PBS-tween (0.05%) with 5% dry milk for 30 min, incubated with 5 µg/mL of O6-mFc in PBS-tween with 1% dry milk overnight at 4 °C, washed x4 in PBS-tween, incubated with anti-mouse IgG-HRP at 5 µg/mL for 1 h at room temperature and detected with SuperSignal West Pico PLUS Chemiluminescent Substrate. Glycoproteins were treated with PNGaseF and/or neuraminidase A according to the protocol recommended by the manufacturer (New England Biolabs).

Sulfatide and porcine gangliosides were purchased from Avanti Polar Lipids, resuspended in CHCl_3_:MeOH (2:1, by volume) and run on aluminum backed silica gel high-performance thin-layer chromatography plates (TLC) using the following developing solution CHCl_3_:MeOH:0.2% CaCl_2_ (60:40:9, by volume). Separation of the porcine gangliosides and sulfatide was verified by staining dried TLC plates with 0.1% orcinol in 5% sulfuric acid. The plates were immediately warmed on a hot plate until the sugars could be visualized (Supplementary Fig. S12). To detect O6 binding to the lipid compounds, after separation, the dried chromatograms were incubated for 90 seconds in a 0.5% (w/v) solution of poly-isobutyl methacrylate beads dissolved in hexane. The TLC plates were dried again, and then directly blocked with 5% dried milk in PBS-tween for 30 min at room temperature. The plates were directly probed with the O6-mFc recombinant protein, and detected as described for the western blotting procedure. Complete western and lipid blots can be found in Supplementary Fig. S12.

ELISA plates were coated overnight with protein at 5 µg/mL at 4 °C. Lipids were diluted to 5 µg/mL in 100% MeOH and placed at room temperature overnight until the MeOH evaporated. For blocking, 200 µL of PBS with 1% BSA was added to each well and incubated at 37 °C for 2 h. O6-mFc was diluted in 50 µL of PBS with 1% BSA to a concentration of 0, 0.4, 2, and 10 µg/mL for 1 h at 37 °C. Detection was completed with an anti-mouse IgG-HRP at 5 µg/mL and the SuperSignal ELISA Femto Substrate. Washes were performed using PBS with 0.5% tween (PBS-tween).

### Flow cytometry analysis of CHO cells

All of the CHO cell lines were grown in DMEM media with 10% fetal bovine serum and Pen/Strep according to standard methods. Adherent cells were collected using Trypsin with 0.05% EDTA and fixed in 4% PFA. Cells were then incubated with 0.4, 2, and 10 µg/mL of O6-mFc in PBS with 1% BSA for 1 h at 4 °C. Cells were washed with PBS-tween four times, and then incubated with an anti-mouse IgG-488 secondary reagent in PBS with 1% BSA for 1 h at 4 °C. A secondary only incubation was used as a negative control, and cells were analyzed by standard flow cytometry methods. The gating strategy and individual histograms can be found in Supplementary Fig. S13.

### Isolation and mass spectrometry analysis of N-glycans from glycoproteins

Twenty milligrams of asialofetuin was treated with PNGaseF following the given protocol by New England Biolabs, with an extended incubation for 2 days at 37 °C. The released glycans were purified in tandem by C18 Sep-Pak and Carbograph columns and lyophilized. The purified glycans were then run over a strong anion exchange column (SAX) that was preconditioned with 200 mM NaOAc and flow-through was collected. Glycans were eluted with 1 M NaCl and purified with a Carbograph column. Glycans were then permethylated following the protocol described in Khoo et al 2010^24^. Permethylated glycans were analyzed in both positive and negative ion mode by MALDI MS.

### Immunohistochemical analysis

Staining was performed on paraffin-embedded tissue microarrays from USBiomax (#MN0661 and #UNC241). Primary staining was done using 20 μg/mL (#MN0661) and 10 µg/mL (#UNC241) of O6, incubated at RT for 2 h. Anti-mouse HRP (Sigma) was used as secondary for 1 h at RT, slides were developed for 4 min with AEC single solution and then stained with Hematoxylin for 5 min. Pictures were acquired with an Olympus DP71. Neuraminidase (from Roche) treatment was performed for 2 h at 37 °C.

### Optimization of glycoprotein microarray printing concentration and substrate

The initial glycoprotein microarray was actually a mucin microarray consisting of mucins and mucin-like glycoproteins. These glycoproteins included bovine submaxillary mucin (BSM, Calbiochem, Cat #499643, Lot #B37307), porcine stomach mucin Type II (Sigma–Aldrich, Cat #M2378, Lot #33F-C756), porcine stomach mucin Type III (Sigma–Aldrich, Cat #M1778, Lot #SLBG4336V), normal and cystic fibrosis sputum mucin (as previously described^48^), glycophorin A (Sigma–Aldrich, Cat #G9511, Lot #50H40651), and bovine serum albumin (BSA, Sigma–Aldrich, Cat #A0281, Lot #89H7604). These glycoproteins or the BSA (a control for nonspecific protein binding) were dissolved in Milli-Q filtered water and 0.2 µm filtered. The glycoproteins were then 10-fold serially diluted to 100, 10, and 1 µg/ml in 0.1 M sodium phosphate buffer pH 8.0 as the final buffer. 10 µl of each glycoprotein at the three concentrations as well as 10 µl of 0.1 M sodium phosphate buffer alone (buffer control) were transferred to a 384-well polypropylene conical bottom plate (source plate). 100 µM of a glycan control (NA2,6-AEAB, Neu5Acα2-6Galβ1-4GlcNAcβ1-2Manα1-3(Neu5Acα2-6Galβ1-4GlcNAcβ1-2Manα1-6)Manβ1-4GlcNAcβ1-4GlcNAc-AEAB, where AEAB = 2-ethyl-N-(aminoethyl)benzamide) was also printed as a control for efficient attachment to the NHS slide. 0.1 µg/ml biotin-hydrazide was also included as a control for Streptavidin probe activity on the NHS slides.

Printing was performed on N-hydroxysuccinimide-coated slides (NEXTERION^®^ Slide H, Schott) or nitrocellulose-coated slides (FAST slides, Whatman) in 16-subarray formats. All samples were printed in six replicates each on 16 subarrays on one NHS- and one nitrocellulose-coated slide with a sciFLEXARRAYER Printer (Scienion). Following printing, the NHS slide was incubated in a humidified 55 °C chamber for 2 h while the nitrocellulose-coated (NC) slide was incubated overnight at room temperature. The NHS slide was then blocked with Super G blocking buffer (Grace-Bio). The NC slide was stored at −20 °C, and the subarray(s) of interest were blocked with TSM binding buffer (TSMBB; 20 mM Tris pH 7.4, 150 mM NaCl, 2 mM MgCl_2_, 2 mM CaCl_2_, 0.05% v/v Tween-20, 1% w/v BSA) immediately prior to subarray screening.

The screening of the NHS and nitrocellulose slides was performed as follows. The NHS and NC slide were both removed from −20 °C and dried in a vacuum desiccator for at least 20 min. A ProPlate 16-well chamber (Grace-Bio) was applied to both slides. The subarrays of interest on the NHS slide were rehydrated for at least 5 min with TSM Wash Buffer (TSMWB; 20 mM Tris pH 7.4, 150 mM NaCl, 2 mM MgCl_2_, 2 mM CaCl_2_, 0.05% v/v Tween-20), while the subarrays of interest on the NC slide were blocked for 1 h with TSMBB as described above. All incubations were performed at room temperature on a low-speed orbital shaker (~50 rpm). Biotinylated peanut agglutinin (PNA, Vector Labs) was diluted in TSMBB to 10, 1, and 0.1 µg/ml final for screening on both microarray formats. After aspirating the rehydration or blocking solution, 70 µl of each concentration of biotinylated PNA was applied to one subarray each on both microarray formats. The chambers were sealed with an adhesive strip, and the slides were incubated for 1 h. The subarrays on both slides were then washed four times for a few seconds with 0.2 ml of TSMWB followed by four brief washes with 0.2 ml of TSM (20 mM Tris pH 7.4, 150 mM NaCl, 2 mM MgCl_2_, 2 mM CaCl_2_). Next, 70 µl of 0.5 µg/ml Streptavidin-Cy5 (Molecular Probes) was added to each subarray. The chamber was sealed with an adhesive strip, and the slides were incubated in the dark for 1 h. The subarrays were then washed four times for 5 minutes each with 0.2 ml of TSMWB followed by four brief washes with TSM. The slides were rinsed with deionized water and spun down to dry. The two slides were then scanned with a GenePix 4300 A scanner with the 635 nm laser, Standard Red Channel, using the same PMT gain and laser power for both slide scans.

Additional biotinylated lectin and anti-glycan antibody screenings were performed on this mucin microarray on the NC slide only, which revealed that all of the glycoproteins except glycophorin gave the expected binding pattern. Specifically, glycophorin appeared heavily desialylated as it was highly reactive with PNA but poorly reactive with MAL-II (data not shown). Therefore, the glycophorin lot used on this microarray was discarded and a new preparation and lot (Sigma–Aldrich, Cat #G5017, Lot #082M4110) was ordered and used on subsequent glycoprotein microarray print runs. This new glycophorin preparation was found to give the expected lectin binding pattern, especially very strong MAL-II binding (data not shown). Besides glycophorin, porcine stomach mucin Type II was also not used in later glycoprotein microarrays since this preparation gave a similar but weaker binding pattern vs. porcine stomach mucin Type III.

### Statistics and reproducibility

Glycans displayed on the CFG glycan microarrays were printed in replicates of six. The mean relative fluorescent units (RFU) was calculated by averaging the fluorescent intensity of four median value glycan spots, which removed the highest and lowest values. From these values, the standard deviation, which is represented in the error bars in each graph, and %CV were calculated. Glycoproteins were printed on nitrocellulose in replicates of four, and all of the values were included in the calculation of the mean RFU and standard deviation, which is represented in the error bars in each graph. The O6 VLR was run at multiple concentrations, to increase confidence in the observed binding patterns and look for concentration-dependent binding interactions.

### Reporting summary

Further information on research design is available in the Nature Research Reporting Summary linked to this article.

## Supplementary information

Supplementary Material

Description of Additional Supplementary Files

Supplementary Data 1

Supplementary Data 2

Supplementary Data 3

Supplementary Data 4

Supplementary Data 5

Supplementary Data 6

Supplementary Data 7

Supplementary Data 8

Supplementary Data 9

Supplementary Data 10

Supplementary Data 11

Reporting Summary

Validation Report

Validation Report

## Data Availability

All glycan and glycoprotein microarray data will be available for review at the National Center for Functional Glycomics (NCFG) website (https://ncfg.hms.harvard.edu). This data are represented in Fig. 1a and Fig. 4. DNA and amino-acid sequences for O6 will be deposited into GenBank (Accession BankIt2428777 O-6.seq MW699437) and crystal structures will be deposited into the RCSB protein data bank (PDB 7LA7, 7LA8). These data can be found in Fig. 2, S1, and S2. There are no restrictions on any of the data found within this manuscript.
