# Supplementary material for: Novel lamprey antibody recognizes terminal sulfated galactose epitopes on mammalian glycoproteins
Source: Commun Biol. 2021 Jun 3;4:674. doi: 10.1038/s42003-021-02199-7 (PMC8175384; doi:10.1038/s42003-021-02199-7)
Supplement: Supplementary file 1 — Supplementary Material [file 42003_2021_2199_MOESM1_ESM.pdf]

Supplementary Material for:

Novel Lamprey Antibody Recognizes Terminal Sulfated Galactose Epitopes on Mammalian Glycoproteins

Tanya R. McKitrick[1,#], Steffen M. Bernard[2,#], Alexander J. Noll[1,3,#], Bernard C. Collins[2], Christoffer K. Goth[1,4], Alyssa M. McQuillan[1], Jamie Heimburg-Molinaro[1], Brantley R. Herrin[5,6], Ian A. Wilson[2,7], Max D. Cooper[3] and Richard D. Cummings[1,8]

[1]Dept. of Surgery, Beth Israel Deaconess Medical Center, Harvard Medical School, Boston, MA, 02215, U.S.A.

[2]Dept. of Integrative Structural and Computational Biology, The Scripps Research Institute, La Jolla, CA, 92037, U.S.A.

[3]Current address: Enteric Disease Dept., Naval Medical Research Center, Silver Spring, MD, U.S.A.

[4]Current address: Dept. of Biomedical Sciences, University of Copenhagen, Copenhagen, Denmark

[5]Dept. of Pathology and Laboratory Medicine, Emory Vaccine Center, Emory University School of Medicine, Atlanta, GA, 30322, U.S.A.

[6]Current address: Acceleron Pharma, Boston, MA, U.S.A.

[7]The Skaggs Institute for Chemical Biology, The Scripps Research Institute, La Jolla, CA, 92037, U.S.A.

[#]These authors contributed equally to this work

[8]To whom correspondence should be addressed:
Richard D. Cummings, Ph.D., Email: rcummin1@bidmc.harvard.edu

Supplementary Results

Results of Glycoprotein Microarray Optimization Experiments

A number of variables were optimized for the glycoprotein microarray. These included the method of slide attachment, concentration for printing, attachment efficiency, slide blocking, washing method, and carrier identity and concentration.

For the printing solution, 0.1M sodium phosphate pH 8.0 buffer was initially used since this buffer has been used for in-house printing onto NHS slides. Upon finding that nitrocellulose was superior to NHS as the printing substrate (as described below), the 0.1M sodium phosphate pH 8.0 buffer was no longer a requirement. Instead, PBS was chosen as the buffer since this buffer was found to improve stability and solubility of some other glycoproteins vs. the 0.1M sodium phosphate pH 8.0 buffer, likely because of the presence of saline. Indeed, all glycoprotein samples utilized to date have been efficiently solubilized in PBS buffer, and the PBS buffer is well-tolerated by the microarray printer. However, the printing solution was not further optimized (for example, with detergents, trehalose or other viscosity normalizers, or carrier molecules such as BSA) beyond the use of PBS due to the efficiency of PBS and the fact that some glycoproteins (ex. transferrin) are highly susceptible to precipitation by some of these additional components.

For slide attachment, nitrocellulose and N-hydroxysuccinimide (NHS) slides were tested with a panel of glycoproteins, especially the mucins since these glycoproteins are especially difficult to handle. This mucin microarray was screened with PNA, a lectin that recognizes the Core 1 mucin-type O-glycan structure Galβ1-3GalNAc, which was expected to be present on all of these mucin samples. It was found that printing on

nitrocellulose gave >10-fold greater sensitivity of detection vs. NHS slides with 10μg/ml PNA (**Supplementary Figure S7a, Supplementary Data 6**) and much lower intra-spot standard deviations of pixel fluorescence (data not shown). This can be more clearly seen by comparing the actual microarray images (**Supplementary Figure S7b**), which showed a regular circular pattern for PNA binding on the NC slide but irregular, non-circular, and diffuse binding on the NHS slides. Moreover, while binding to mucins on the nitrocellulose slide could be detected with 1μg/ml PNA, only 10μg/ml PNA led to weak binding to the mucins on the NHS slide. This irregular spot shape on the NHS slides was likely because of the low lysine content on mucins and hence more quantitative attachment of mucins to nitrocellulose vs. NHS slides. Due to the large number of mucins and mucin-like glycoproteins used on subsequent glycoprotein microarray versions, nitrocellulose was selected as the primary substrate for printing.

For concentration optimization, 1, 10, and 100μg/ml of the mucins were printed on nitrocellulose slides. Concentrations higher than 100μg/ml were not utilized since higher concentrations caused the mucin solutions to become too viscous for efficient and reproducible filtering and printing. It was found that only the 10 and 100μg/ml but not 1μg/ml mucin spots were detectable by the lectins ConA and RCA-I, with a dose-dependent binding effect (**Supplementary Figure S8a,b, Supplementary Data 7**). This dose-dependent binding to the 10 and 100μg/ml spot suggested that the glycoprotein spot was not saturated for at least the 10μg/ml print concentration. PNA binding to the mucin microarray (**Supplementary Figure S7a**) gave a similar result binding to only the 10 and 100μg/ml spots as did other plant lectins including AAL and SNA (data not shown). However, binding to the 10μg/ml spots was typically low sensitivity (low RFU)

and highly variable between replicates, resulting in %CV values >20% in many cases. These results thus pointed to 100µg/ml as the preferable print concentration. Further confirmation of 100µg/ml as the preferred printing concentration came from screenings on the glycoprotein microarray platform that contained multiple glycoproteins in addition to mucins. On this glycoprotein microarray, only the 100µg/ml print concentration was typically bound and, in cases where the 10µg/ml glycoprotein spots were bound, the binding was weak and highly variable (data not shown). Higher lectin concentrations allowed detection of the 10µg/ml samples, but these higher lectin concentrations may cause cross-reactivity to lower affinity determinants, cause non-specific binding, and/or not be practical in some cases with precious samples. Due to the fact that only a very small amount of glycoprotein is printed on a single slide (specifically, nanograms for a 16-subarray format as used for the current glycoprotein microarray platform), it was concluded that 100µg/ml glycoprotein was the optimal concentration for printing. Although higher concentration than 100µg/ml may promote even higher sensitivity, this is not practical since some glycoproteins such as the mucins were too viscous to quantitatively and reproducibly handle at concentrations higher than 100µg/ml.

Despite the sensitivity of the nitrocellulose platform, the background fluorescence was still quite high, as exemplified in **Supplementary Figure S7b** for the 10µg/ml PNA screening. This high background was consistently detectable and became exacerbated when the 488nm laser and Standard Blue channel was used (ex. using Alexa Fluor 488-labeled secondary probes). Nitrocellulose is known to exhibit high autofluorescence, especially at lower wavelengths, which contributes to the high background along with non-specific nitrocellulose binding by the screened protein [2, 3]. However, different

nitrocellulose slide manufacturers claim to have reduced this autofluorescence. Thus, it was of interest to compare the results of screening the same plant lectin on mucin microarrays printed on two different nitrocellulose slides. The slides chosen were FAST slides from Whatman and NOVA slides from Grace-Bio. Mucin microarray printing and mucin immobilization efficiency were similar between these two slides types (data not shown), but it was found that the binding on the NOVA slides gave higher signal:noise ratios (SNRs) and lower background fluorescence intensities than on FAST slides (**Supplementary Figure S9a-c, Supplementary Data 8**). Additionally, SuperNOVA slides from Grace-Bio were also used for glycoprotein microarray printing and were found to give very high SNRs (with RFU values as low as 100 RFU sometimes being detected with SNR ≥ 5.000, the set SNR cutoff for a bound vs. unbound glycoprotein), although a side-by-side comparison with NOVA slides was not tested. Due to the claim by Grace-Bio of reduced background and higher sensitivity of the SuperNOVA vs. NOVA slides and the high sensitivity binding detected in-house, SuperNOVA slides from Grace-Bio were chosen as the slide type to be used for the glycoprotein microarray.

Another variable that was optimized was the nitrocellulose blocking agent. As described above, non-specific nitrocellulose binding is one of two major factors that promote background fluorescence. The addition of a blocking agent substantially reduces this non-specific nitrocellulose binding, but the blocking agent itself may also be non-specifically bound by the screened sample. For these reasons, the blocking agent was optimized. It was found in preliminary studies that 1% w/v BSA gave greater sensitivity and a similar background binding vs. 1% w/v nonfat milk as the blocking agent (data not shown). Additionally, protein and non-protein blockers can be used for

blocking the SuperNOVA nitrocellulose slide, and the Super G Blocking Agent has been promoted as giving superior sensitivity vs. protein-based blocking agents for protein microarrays. Thus, the 1% w/v BSA blocking agent was compared to Super G Blocking Buffer. Specifically, an anti-Blood Group H Type 1- (anti-H1) specific antibody was utilized for this testing because this antibody was consistently seen to give very high background binding to the glycoprotein microarrays during quality control screenings to assess proper glycoprotein glycosylation. This higher than usual background was likely because of the fact that this antibody is a crude ascites fluid sample rather than a purified monoclonal antibody, and in some cases the background binding was very extreme and made the SNR too low for accurate classification of binders. The readout of interest was the SNR, which was expected to be consistently higher for all bound glycoproteins if one of the two blocking agents was superior at reducing background. The results clearly demonstrated that Super G Blocking Buffer was superior to 1% BSA as the blocking agent, resulting in consistently higher SNRs (**Supplementary Figure S10, Supplementary Data 9, 10**). Super G Plus Preservative solution from Grace-Bio combines the blocking of Super G Blocking Buffer with a preservative for long-term -20°C storage of protein microarrays. Testing of Super G Plus as a blocking agent showed that this blocker also produced significantly high sensitivity (data not shown), although a side-by-side comparison with Super G Blocking Buffer for SNR and long-term sensitivity was not tested. Nonetheless, we did notice an increase in non-specific protein binding when the slides were stored at -20°C unblocked and then blocked with Super G Blocking Buffer prior to experimentation, an issue that has not yet been seen to

date with slides blocked with Super G Plus. For these reasons, Super G Plus was chosen as the blocking agent for glycoprotein microarrays.

The method of washing chosen was similar to that of Western blotting, namely four washes for five minutes with a Tween-20-supplemented buffer. TSMWB (20mM Tris pH 7.4, 150mM NaCl, 2mM $MgCl_2$, 2mM $CaCl_2$, 0.05% v/v Tween-20) was more than sufficient for this washing method. Increased the salt or Tween concentration did not significantly improve SNRs, nor did increasing the number of washes and/or wash time, with screened samples producing relatively higher backgrounds. This does not rule out that some washing conditions may be preferred, so other washing methods may be used if other experiments suggest a preferable washing method for a given sample. However, it was found that one wash with Super G Blocking Buffer was sufficient.

A critical factor requiring optimization is the presence and concentration of carriers in the binding buffer. BSA was typically used as the carrier protein, which also serves an additional benefit of blocking non-specific protein binding. Indeed, it was found that different BSA concentrations in the binding buffer influenced the binding pattern, but the effect appears unique to different samples. For example, 0.1% BSA allowed efficient binding of the plant lectin to the glycoprotein microarray, whereas 10% BSA almost completely prevented SNA binding (**Supplementary Figure S11a, Supplementary Data 11**). The most likely explanation for this BSA inhibition of SNA binding is that BSA preparations are typically contaminated with bound lipids, including glycosphingolipids that may cross-react with SNA, as previously suggested. On the other hand, the binding of the antibody CHO-131, which is specific for sialyl Lewis x on

a Core 2 O-glycan backbone (Galβ1-3(Neu5Acα2-3Galβ1-4(Fucα1-3)GlcNAcβ1-6)GalNAcα-), was non-specific with 0.1% BSA. CHO-131 was only expected to bind to the sputum mucins, which have been previously shown to express this glycan structure. However, CHO-131 showed binding to a wide variety of glycoproteins in the presence of 0.1% BSA besides the sputum mucins, one of which was the BSA control itself. This result suggested CHO-131 was non-specifically interacting with proteins. However, raising the BSA concentration to 10% in the blocking buffer eliminated this non-specific BSA and glycoprotein binding, resulting in specific binding to only the sputum mucins. Therefore, the BSA concentration in the binding buffer is empirical and must be optimized for each sample. In optimization screenings, multiple BSA concentrations in the binding buffer are thus tested. Additionally, the BSA control on the glycoprotein microarray is useful for knowing the concentration of BSA to use in the binding buffer since this sample should not be bound. If binding to the printed BSA sample occurs, it indicates that a higher BSA concentration in the binding buffer is needed. It was also found that the addition of Super G Blocking buffer to a concentration of 0.1x final also improves the SNR vs. omission of this carrier (**Supplementary Figure S11b**), as has been previously shown by others. Therefore, the binding buffer should be supplemented with 0.1x Super G and an experimentally verified concentration of BSA for optimal results. It should be noted though that the Super G blocking Buffer and Super G Plus have been found to quench fluorophores, including Cy5 and Alexa Fluor 488, and thus cannot be included in any steps beginning at or following the addition of the fluorescently-labeled probe (ex. fluorescently labeled probe’s binding buffer or the subsequent wash steps).

Supplementary Table S1. O6 recognizes a subset of sulfated CFG glycans which contain a terminal 3-O-SGal. Glycans ordered from high to low binding over 3 concentrations of O6; RFU = relative fluorescence units.

CFG ID	Glycan Sequence	RFU - 2ug	StDev	% CV	RFU - 10ug	StDev	% CV	RFU - 50ug	StDev	% CV
35	(3S)Galb1-4(6S)GlcNAcb-Sp8	7279	152	2	12602	387	3	18213	802	4
37	(3S)Galb1-4GlcNAcb-Sp8	1950	143	7	7226	458	6	14689	805	5
34	(3S)Galb1-4(6S)GlcNAcb-Sp0	6688	471	7	12643	355	3	13067	738	6
36	(3S)Galb1-4GlcNAcb-Sp0	1616	99	6	6298	463	7	12444	259	2
219	(3S)Galb1-4(Fuca1-3)(6S)GlcNAcb-Sp8	929	111	12	3433	298	9	8620	645	7
515	(3S)GalNAcb1-4GlcNAc-Sp8	323	33	10	1958	186	10	5424	256	5
22	6S(3S)Galb1-4(6S)GlcNAcb-Sp0	66	17	26	793	98	12	2064	131	6
297	(6S)Galb1-4(6S)GlcNAcb-Sp0	9	4	45	75	16	21	176	31	18
44	(6S)Galb1-4GlcNAcb-Sp8	1	1	170	54	19	34	134	44	33
25	(3S)Galb1-4Glcb-Sp8	2	5	224	17	4	21	121	20	16
26	(3S)Galb1-4(6S)Glcb-Sp0	3	5	170	1	3	339	63	5	8
27	(3S)Galb1-4(6S)Glcb-Sp8	8	6	78	23	12	53	54	19	36
38	(3S)Galb-Sp8	6	6	110	11	2	18	26	17	65
29	(3S)Galb1-3GalNAca-Sp8	6	5	91	17	4	26	16	10	64
513	(3S)GalNAcb1-4(3S)GlcNAc-Sp8	4	3	81	3	6	191	13	10	71
492	(3S)Galb1-3(Fuca1-4)GlcNAcb-Sp0	10	5	46	4	2	55	8	2	29
28	(3S)Galb1-3(Fuca1-4)GlcNAcb-Sp8	6	4	68	7	10	136	7	3	48
31	(3S)Galb1-3GlcNAcb-Sp8	11	10	92	20	10	47	7	7	111
84	(3S)Galb1-4(Fuca1-3)Glcb-Sp0	7	10	141	-1	4	-582	6	4	63
32	(3S)Galb1-4(Fuca1-3)GlcNAc-Sp0	2	3	152	10	6	65	5	6	109
24	(3S)Galb1-4(Fuca1-3)(6S)Glc-Sp0	12	7	62	2	2	97	4	4	100
569	(3S)GlcAb1-3Galb1-4GlcNAcb1-3Galb1-4Glc-Sp0	0	2	349	5	14	263	4	6	147
23	6S(3S)Galb1-4GlcNAcb-Sp0	6	6	98	12	5	38	3	3	93
33	(3S)Galb1-4(Fuca1-3)GlcNAc-Sp8	15	8	58	11	4	36	3	5	157
570	(3S)GlcAb1-3Galb1-4GlcNAcb1-2Mana-Sp0	16	4	24	6	2	42	3	5	159
504	(3S)GalNAcb1-4(Fuca1-3)GlcNAcb-Sp8	4	1	28	5	6	122	3	3	126
30	(3S)Galb1-3GlcNAcb-Sp0	12	7	59	3	1	44	2	4	228
296	4S(3S)Galb1-4GlcNAcb-Sp0	4	5	119	5	3	51	2	3	156
40	(4S)Galb1-4GlcNAcb-Sp8	2	7	314	6	6	87	4	5	109
512	(6S)GalNAcb1-4GlcNAc-Sp8	5	3	61	5	7	145	1	4	380
443	(6S)Galb1-3GlcNAcb-Sp0	-1	3	-407	12	21	177	-2	2	-132
39	(6S)(4S)Galb1-4GlcNAcb-Sp0	6	8	141	20	7	36	22	26	120
355	(6S)GlcNAcb1-3Galb1-4GlcNAcb-Sp0	16	13	84	11	3	28	11	4	38
444	(6S)Galb1-3(6S)GlcNAc-Sp0	5	3	49	17	20	122	11	2	22
518	(6P)Galb1-4GlcNAcb-SP0	-1	2	-356	-1	2	-174	3	6	165
517	Galb1-4(6P)GlcNAcb-Sp0	17	14	82	9	7	70	13	8	64
155	Galb1-4(6S)Glcb-Sp0	6	3	55	6	3	53	11	10	90
41	(6P)Mana-Sp8	4	7	174	6	1	8	10	9	90
251	Neu5Aca2-3Galb1-4(6S)GlcNAcb-Sp8	9	3	31	3	4	122	9	12	131
298	(6P)Glcb-Sp10	6	6	113	4	4	90	8	3	34
516	(4S)GalNAcb-Sp10	9	5	49	8	9	110	8	3	41
222	Fuca1-2(6S)Galb1-4(6S)Glcb-Sp0	10	8	79	2	3	136	8	4	47
221	Fuca1-2Galb1-4(6S)GlcNAcb-Sp8	6	6	97	6	3	47	8	6	80
290	Galb1-4(Fuca1-3)(6S)GlcNAcb-Sp0	9	4	41	4	6	156	8	1	11
242	Neu5Aca2-3Galb1-3(6S)GalNAca-Sp8	7	8	116	15	7	44	7	7	100
45	(6S)Galb1-4(6S)Glcb-Sp8	4	5	128	4	6	146	7	4	54
267	Neu5Aca2-6Galb1-4(6S)GlcNAcb-Sp8	6	3	58	3	3	118	7	2	30
220	Fuca1-2(6S)Galb1-4GlcNAcb-Sp0	6	4	66	-3	6	-195	6	2	33
230	Neu5Aca2-3(6S)Galb1-4(Fuca1-3)GlcNAcb-Sp8	-1	2	-122	-2	5	-298	6	7	111
262	Fuca1-2Galb1-4(6S)Glcb-Sp0	6	6	96	-2	3	-168	6	7	122
501	Fuca1-2(6S)Galb1-3(6S)GlcNAcb-Sp0	1	3	287	1	10	730	6	4	72
46	Neu5Aca2-3(6S)Galb1-4GlcNAcb-Sp8	2	3	115	2	9	382	5	3	66
510	Galb1-3(6S)GlcNAcb-Sp8	4	1	27	-1	1	-191	5	8	176
497	Fuca1-2(6S)Galb1-3GlcNAcb-Sp0	4	7	179	10	16	162	5	8	172
252	Neu5Aca2-3Galb1-4(Fuca1-3)(6S)GlcNAcb-Sp8	6	2	31	5	7	126	4	2	43
47	(6S)GlcNAcb-Sp8	6	4	69	6	6	105	4	4	102
514	GalNAcb1-4(6S)GlcNAc-Sp8	6	5	77	7	9	129	4	7	207
502	Neu5Aca2-6GalNAcb1-4(6S)GlcNAcb-Sp8	3	2	77	8	2	19	3	3	92
511	(6S)(4S)GalNAcb1-4GlcNAc-Sp8	6	3	52	6	5	96	3	2	90
238	Neu5Aca2-3Galb1-3(6S)GlcNAc-Sp8	7	12	175	12	7	54	3	8	304
156	Galb1-4(6S)Glcb-Sp8	11	4	36	7	9	135	2	5	237
291	Galb1-4(Fuca1-3)(6S)Glcb-Sp0	7	6	82	6	6	107	2	8	463
42	(6S)Galb1-4Glcb-Sp0	6	3	43	-6	1	-15	0	1	232
43	(6S)Galb1-4Glcb-Sp8	5	5	87	5	3	64	0	2	610
248	Fuca1-2(6S)Galb1-4Glcb-Sp0	6	6	110	-3	1	-42	-1	1	-101
503	GalNAcb1-4(Fuca1-3)(6S)GlcNAcb-Sp8	7	5	68	2	2	145	-1	4	-275
500	Fuca1-2Galb1-3(6S)GlcNAcb-Sp0	-5	1	-21	5	10	213	-3	4	-136

Supplementary Table S2. Compilation of O6 positive staining and expression of the sulfotransferases (GAL3ST-2 and GAL3ST-3) from the human proteome atlas. Immunohistological screening was done on multiple organs and tissues as described in materials and methods. Positive staining is denoted as +, and increase in staining after neuraminidase treatment as +*. Columns GAL3ST2 and GALST3 reflect expression data form the Human Protein Atlas.

Tissue	O6	GAL3ST2	GALST3
Adrenal Gland	-	-	-
Bladder	-	-	-
Bones	-	N/A	N/A
Brain - Cerebellum	+	N/A	N/A
Brain - Cerebral Cortex	-	-	+
Brain - Pituitary	+*	N/A	N/A
Eye	+	N/A	N/A
Female tissues - Fallopian Tube	+	+	+
Female tissues - Ovary	-	-	-
Female tissues - Cervix	-	-	-
Female tissues - Endometrium	+*	-	+
Female tissues - Placenta	-	-	-
G.I. - Esophagus	+*	-	-
G.I. - Stomach	-	-	-
G.I. - Small Intestsine	-	-	-
G.I. - Colon	+	+	-
G.I. - Rectum	+	+	-
Heart	-	-	+
Kidney	+*	-	+
Liver	-	-	-
Lung	-	-	-
Male tissues - Prostate	+*	-	+
Male tissues - Testis	-	-	+
Pancreas	-	-	-
Skin	-	-	-
Spinal Cord	-	N/A	N/A
Spleen	-	-	-
Striated muscle	-	-	-
Thymus	-	N/A	N/A
Thyroid	+	-	+
Tonsil	-	-	-

Supplementary Table S3. Data collection and refinement statistics.

	O6 (Apo)	O6 3-HSO3-LacNAc
**Data collection**		
Space group	C2	I4
Cell dimensions		
a, *b*, *c* (Å)	52.3, 38.1, 78.6	107.3 107.3 63.2
α, β, γ (°)	90, 103.9, 90	90, 90, 90
Resolution (Å)	28.0-1.55 (1.61-1.55) *	38.2 – 1.90 (2.01-1.90)
No. unique reflections	21990	28496
$^{a}R_{sym}$	9.9 (81.5)	11.4 (167.3)
$^{a}R_{pim}$	4.4 (50.1)	4.8 (85.5)
$^{b}CC_{1/2}$	89.4 (53.3)	99.8 (55.9)
$I / \sigma I$	12.7 (1.2)	13.3 (1.2)
Completeness (%)	99.6 (96.2)	99.9 (99.8)
Redundancy	5.7 (3.2)	6.8 (6.7)
**Refinement**		
Resolution (Å)	1.55	1.9
No. reflections	21,895	28,485
$^{c}R_{work}$ / R_{free}	15.9 / 20.1	18.4 / 22.9
No. atoms		
Protein	1370	2590
Ions	10	
Ligand		60
Water	139	199
B-values (Å^2)		
Protein	20	33
Ion	22	
Ligand		22
Water	31	46
Wilson B-value	11	36
R.m.s. deviations		
Bond lengths (Å)	0.016	0.015
Bond angles (°)	1.36	1.40
Ramachandran (%)		
Allowed	100	100
Outliers	0	0

*Values in parentheses are for highest-resolution shell.

[a] $R_{sym} = \Sigma_{hkl} \Sigma_i | I_{hkl,i} - \langle I_{hkl} \rangle | / \Sigma_{hkl} \Sigma_i I_{hkl,i}$ and $R_{pim} = \Sigma_{hkl} (1/(n-1))^{1/2} \Sigma_i | I_{hkl,i} - \langle I_{hkl} \rangle | / \Sigma_{hkl} \Sigma_i I_{hkl,i}$, where $I_{hkl,i}$ is the scaled intensity of the ith measurement of reflection h, k, l, $\langle I_{hkl} \rangle$ is the average intensity for that reflection, and n is the redundancy.

[b] $CC_{1/2}$ = Pearson correlation coefficient between two random half datasets.

[c] $R_{work} = \Sigma_{hkl} | F_o - F_c | / \Sigma_{hkl} | F_o |$ x 100, where F_o and F_c are the observed and calculated structure factors, R_{free} was calculated as for R_{work}, but on a test set comprising 5% of the data excluded from refinement.

[d] Calculated with MolProbity [70].

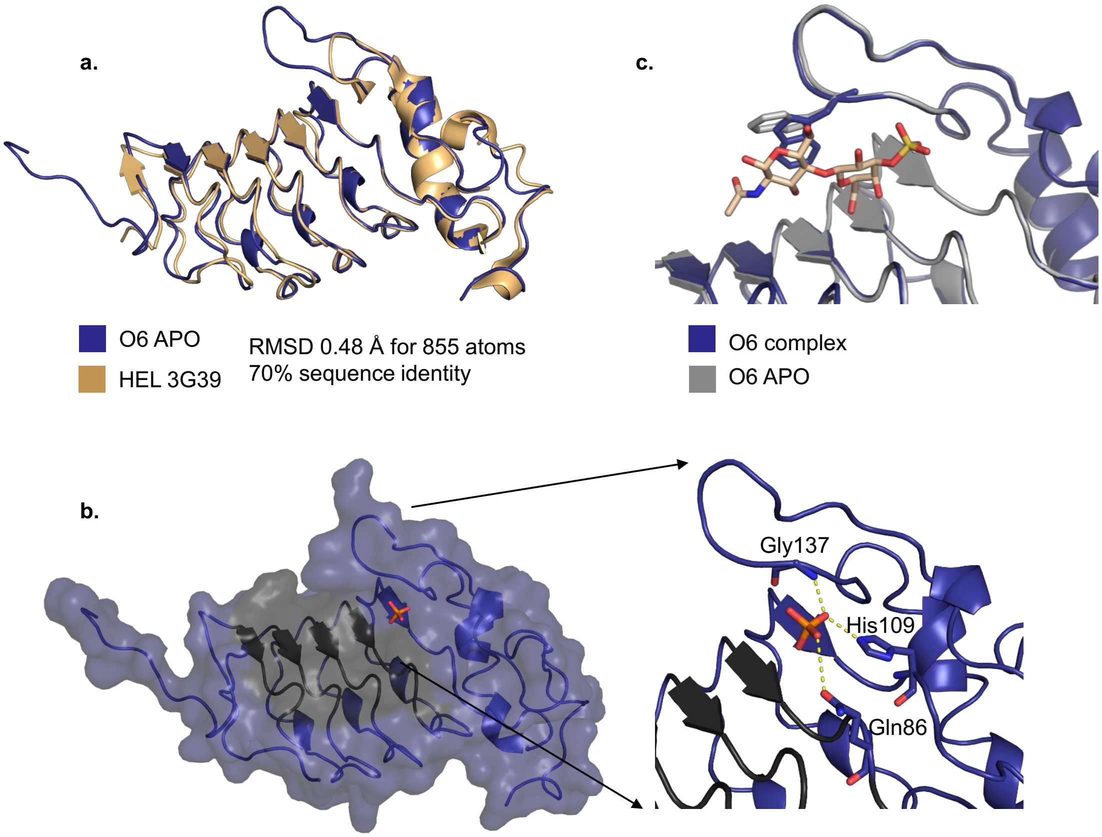

Supplementary Figure S1. **A**. Overlay of apo crystal structure of VLR O6 with VLR 3G39 that is specific for hen egg lysozyme (HEL). Crystal structures for both are quite similar despite their 70% sequence identity. **B.** Apo crystal structure of O6 shows a phosphate ion interacting with Gly136, His109 and Gln86, similar to the sulfate recognition site. **C.** Detailed overlay of the apo and ligand-bound crystal structures suggests that the binding site is preconfigured.

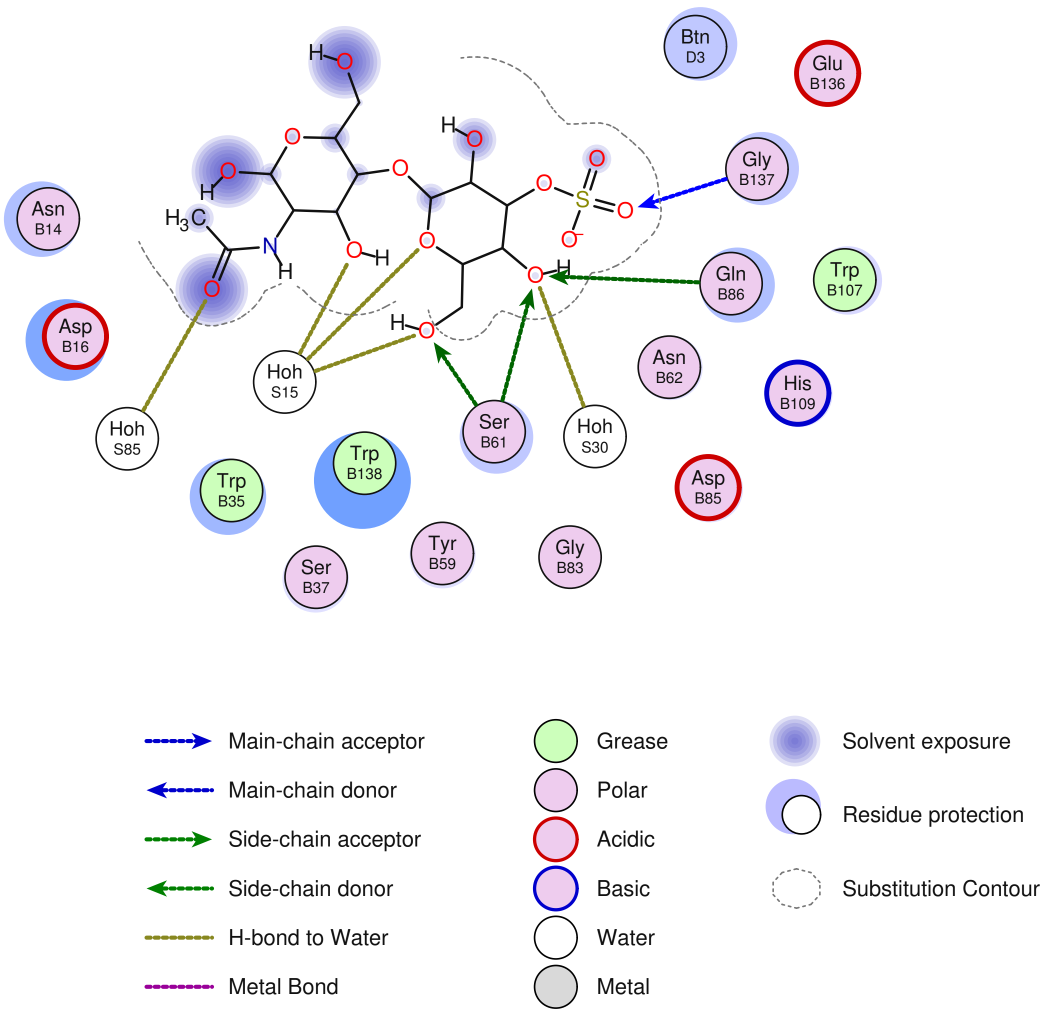

Supplementary Figure S2. Detailed view of the key interactions occurring between O6 and (3S)Galβ1-4GlcNAc ligand.

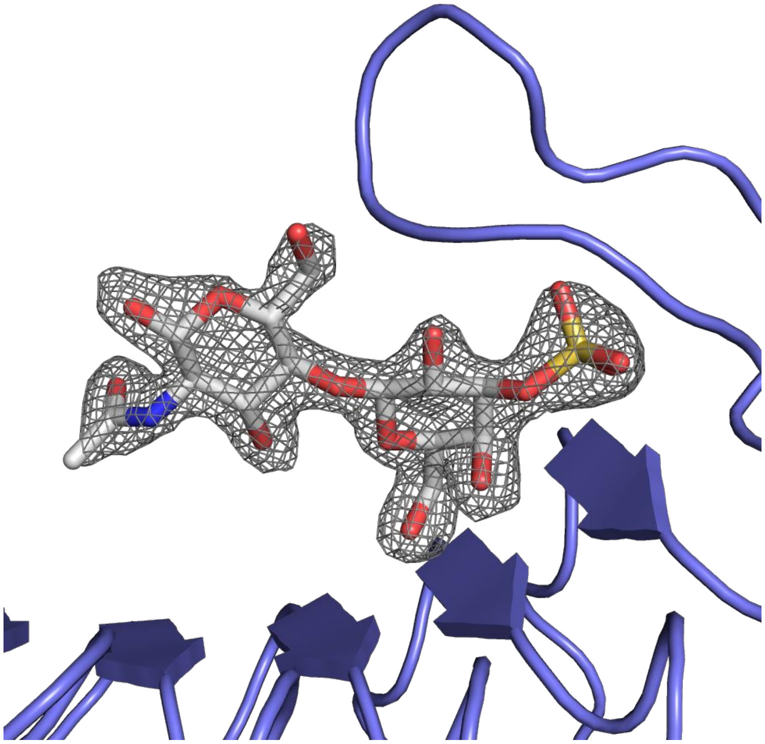

Supplementary Figure S3. Election density OMIT map of the (3S)Galβ1-4GlcNAc ligand. The 2FO-FC simulated annealing omit map at 1.5 sigma shows clear electron density for all features of the ligand.

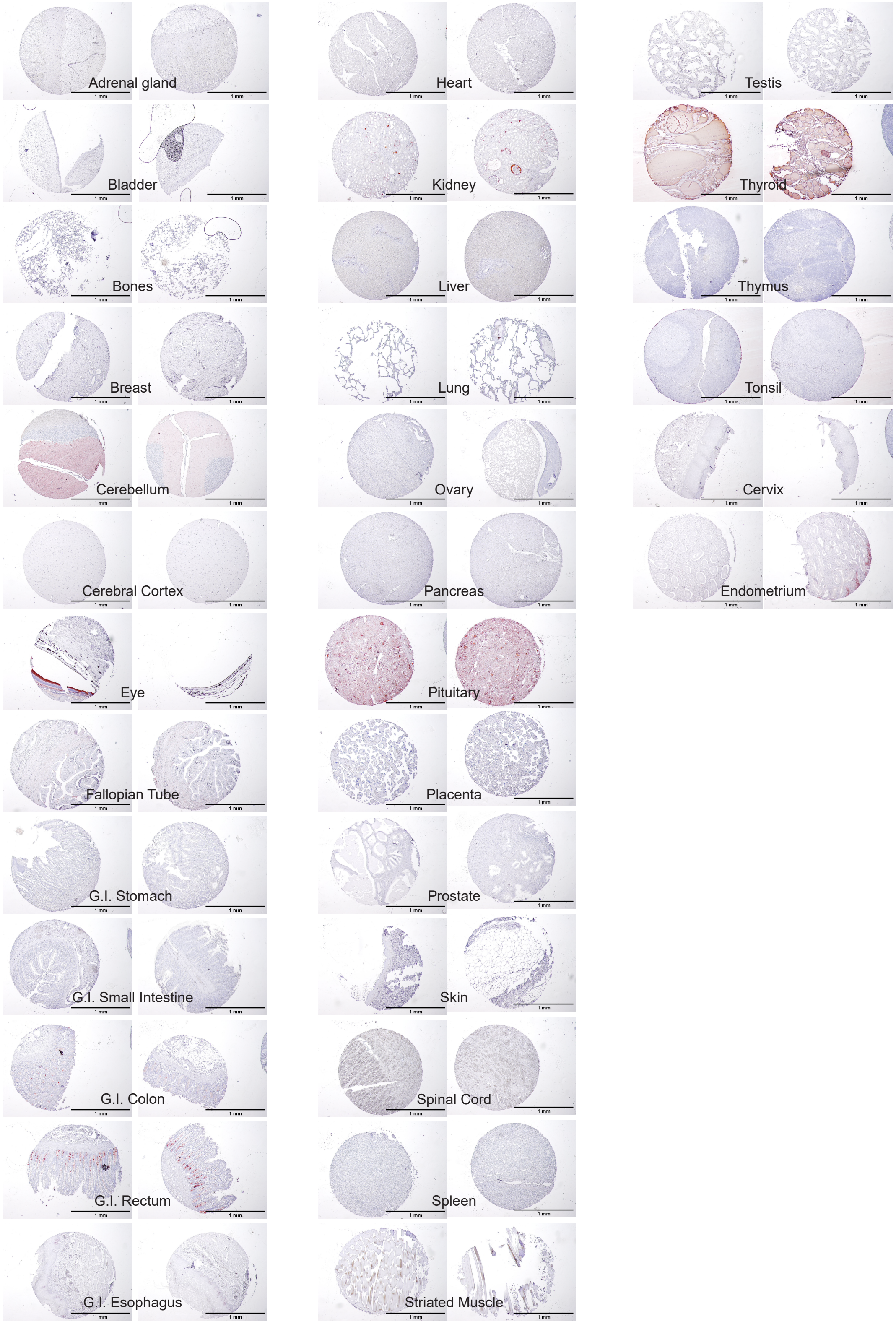

Supplementary Figure S4. IHC staining profiles of the all tissues on the human tissue macroarray.

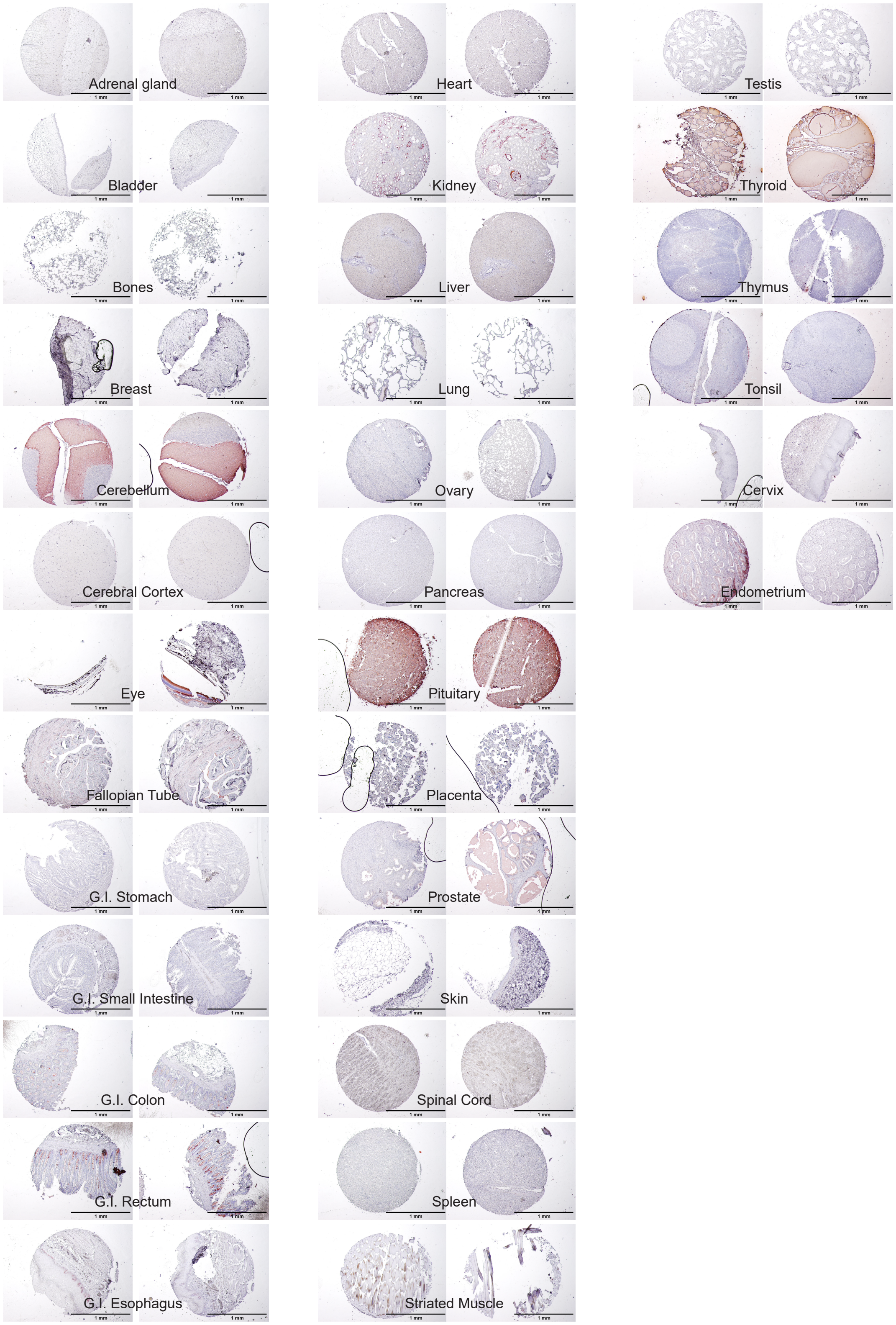

Supplementary Figure S5. IHC staining profiles of the all tissues on the human tissue macroarray after neuraminidase treatment.

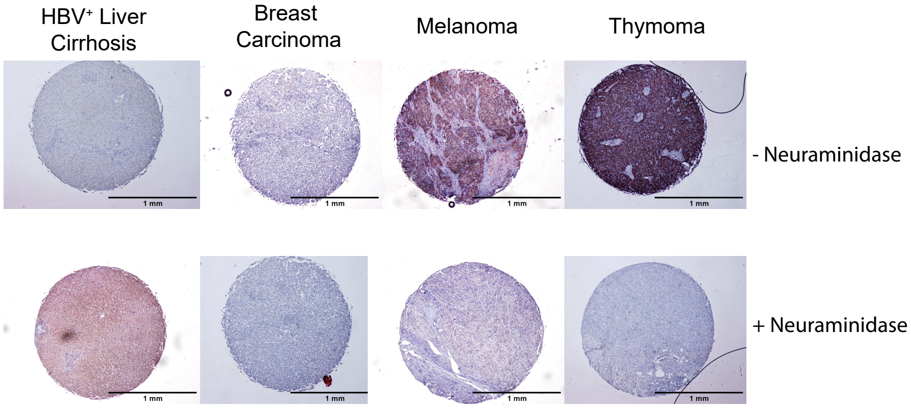

Supplementary Figure S6. IHC staining profiles of the diseased and cancerous tissues on the human tissue macroarray before and after neuraminidase treatment.

a)

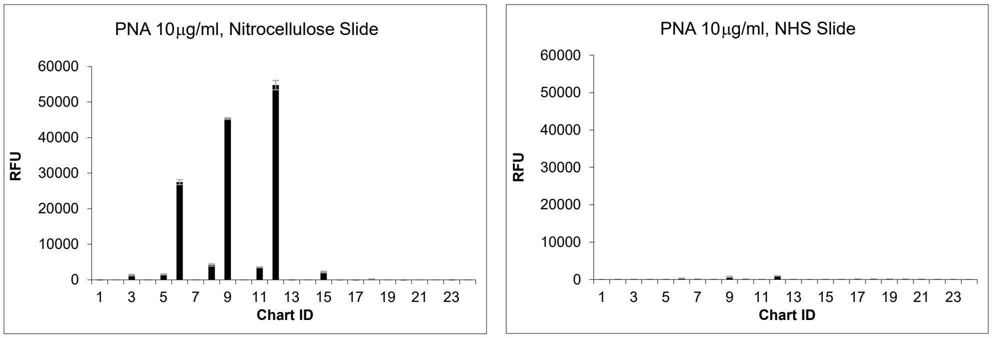

b) Nitrocellulose

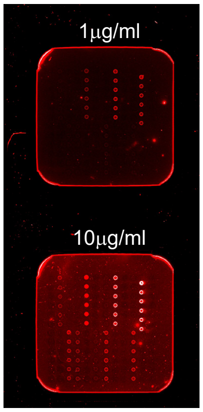

c) NHS

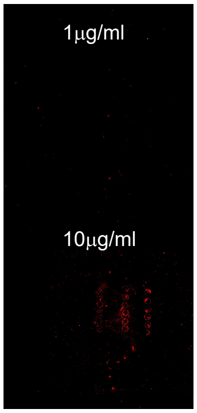

d)

Chart ID	Glycoprotein	Print Concentration (µg/ml)	Average RFU	STDEV	%CV	Average SNR
1	Bovine Submaxillary Mucin	1	-88	34	-39	-0.351
2	Bovine Submaxillary Mucin	10	-73	26	-35	-0.277
3	Bovine Submaxillary Mucin	100	1323	205	15	3.105
4	Porcine Stomach Mucin, Type II	1	-27	30	-113	-0.123
5	Porcine Stomach Mucin, Type II	10	1526	152	10	2.952
6	Porcine Stomach Mucin, Type II	100	27475	710	3	60.748
7	Porcine Stomach Mucin, Type III	1	-22	11	-51	-0.099
8	Porcine Stomach Mucin, Type III	10	4207	321	8	10.503
9	Porcine Stomach Mucin, Type III	100	45335	244	1	92.752
10	Glycophorin A	1	-40	34	-85	-0.217
11	Glycophorin A	10	3541	126	4	9.538
12	Glycophorin A	100	54753	1338	2	48.657
13	Normal Sputum Mucin (N1)	1	-82	39	-48	-0.373
14	Normal Sputum Mucin (N1)	10	-14	18	-128	-0.111
15	Normal Sputum Mucin (N1)	100	2185	221	10	6.641
16	Cystic Fibrosis Sputum Mucin (CF1)	1	-125	24	-20	-0.326
17	Cystic Fibrosis Sputum Mucin (CF1)	10	-146	21	-15	-0.479
18	Cystic Fibrosis Sputum Mucin (CF1)	100	208	13	6	0.228
19	Bovine Serum Albumin (BSA)	1	-63	23	-37	-0.191
20	Bovine Serum Albumin (BSA)	10	-137	21	-15	-0.356
21	Bovine Serum Albumin (BSA)	100	-64	16	-25	-0.309

Supplementary Figure S7. Comparison of NHS vs. Nitrocellulose Slide for Mucin microarray Printing. **A.** Graphs showing the average RFU of 10µg/ml of PNA to the FAST nitrocellulose slide (left graph) vs. Schott NHS slide (right graph). Error bars represent the standard deviation.

Refer to panel d) for the identities of the chart ID numbers. **B.** Scanned microarray image of the 1μg/ml and 10μg/ml PNA binding to two separate subarrays on the nitrocellulose slide. Fluorescence is pseudo-colored red for contrast. White pixels indicate detector-saturated pixels. **C.** Scanned microarray image of the 1μg/ml and 10μg/ml PNA binding to two separate subarrays on the NHS slide. Fluorescence is pseudo-colored red for contrast. This image was scanned under similar PMT and laser power settings as the nitrocellulose slide, and the images are set to the same brightness and contrast settings. **D.** Table of the average RFU, standard deviation, %CV, and average SNR values for nitrocellulose mucin microarray screening with the 10μg/ml PNA. Mucins that were classified as "bound" (defined as an SNR ≥ 5.000) have the SNR value highlighted in green, while mucins that were classified as unbound (defined as an SNR < 5.000) have the SNR value highlighted in red.

a)

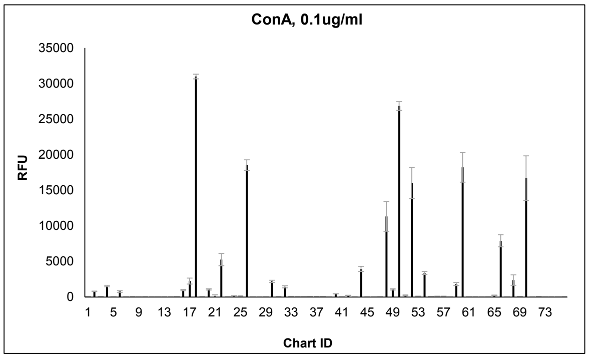

Chart ID	Glycoprotein	Print Concentration (μg/ml)	Average RFU	STDEV	%CV	Average SNR
17	IgM	10	2241	414	18	40.692
18	IgM	100	30985	329	1	515.510
19	IgG, Human	10	39	18	47	0.219
20	IgG, Human	100	1065	83	8	34.285
21	IgG, Bovine	10	119	187	156	0.402
22	IgG, Bovine	100	5264	863	16	6.766
25	α2-Macroglobulin	10	109	58	53	0.909
26	α2-Macroglobulin	100	18516	770	4	266.488
47	Laminin	10	36	17	47	1.133
48	Laminin	100	11333	2097	18	241.732
49	Thyroglobulin, Human	10	1068	88	8	15.615
50	Thyroglobulin, Human	100	26836	605	2	163.054
51	Thyroglobulin, Bovine	10	234	61	26	3.644
52	Thyroglobulin, Bovine	100	16003	2196	14	112.668
53	Ovalbumin	10	95	22	23	0.686
54	Ovalbumin	100	3396	213	6	65.669
55	Ovalbumin, Periodate-Treated	10	74	21	28	1.800
56	Ovalbumin, Periodate-Treated	100	81	34	41	1.925
59	Invertase	10	1815	212	12	22.850
60	Invertase	100	18215	2078	11	182.581
65	*S. mansoni* Egg Antigen (SEA)	10	226	37	17	12.404
66	*S. mansoni* Egg Antigen (SEA)	100	7890	854	11	379.906
67	Haptoglobin	10	18	7	37	0.871
68	Haptoglobin	100	2374	735	31	104.096
69	sIgA	10	61	17	28	2.862
70	sIgA	100	16705	3139	19	740.419

b)

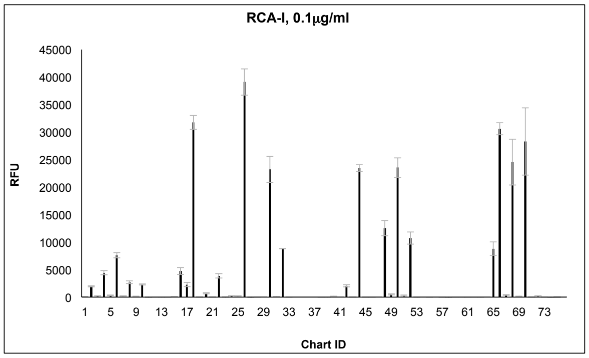

Chart ID	Glycoprotein	Print Concentration (μg/ml)	Average RFU	STDEV	%CV	Average SNR
3	Porcine Stomach Mucin, Type III	10	213	45	21	14.016
4	Porcine Stomach Mucin, Type III	100	4429	364	8	271.508
5	Normal Human Sputum Mucin	10	303	79	26	20.330
6	Normal Human Sputum Mucin	100	7600	463	6	417.756
7	Human Cystic Fibrosis Sputum Mucin	10	155	89	58	9.476
8	Human Cystic Fibrosis Sputum Mucin	100	2680	239	9	143.007
15	IgA	10	92	16	17	5.936
16	IgA	100	4753	632	13	266.332
17	IgM	10	2271	386	17	108.878
18	IgM	100	31756	1261	4	548.420
21	IgG, Bovine	10	7	7	96	0.302
22	IgG, Bovine	100	3858	386	10	250.727
23	apo-Transferrin	10	1	3	294	0.021
24	apo-Transferrin	100	250	21	8	16.593
25	α2-Macroglobulin	10	145	80	55	6.600
26	α2-Macroglobulin	100	39096	2378	6	1640.771
31	Fibronectin	10	104	35	34	5.242
32	Fibronectin	100	8824	48	1	542.038
41	Asialofetuin	10	12	7	61	0.747
42	Asialofetuin	100	2050	187	9	126.689
47	Laminin	10	25	6	23	1.365
48	Laminin	100	12518	1379	11	669.085
49	Thyroglobulin, Human	10	492	94	19	23.159
50	Thyroglobulin, Human	100	23564	1776	8	1033.701
51	Thyroglobulin, Bovine	10	274	88	32	13.958
52	Thyroglobulin, Bovine	100	10750	1112	10	372.248
65	*S. mansoni* Egg Antigen (SEA)	10	8816	1217	14	529.292
66	*S. mansoni* Egg Antigen (SEA)	100	30616	1113	4	1023.914
67	Haptoglobin	10	409	74	18	18.213
68	Haptoglobin	100	24513	4167	17	717.111
69	sIgA	10	154	26	17	7.585
70	sIgA	100	28279	6117	22	1133.749

Supplementary Figure S8. Graphs and a tabular list of glycoproteins specifically recognized by **A**) ConA and **B**) RCA-I are shown. Graphs show the average RFU of binding, and error bars represent the standard deviation. The tables for ConA and RCA-I are shown below the corresponding graphs. Note that each set of two chart IDs represents the same glycoprotein at 10μg/ml and 100μg/ml. Glycoproteins that were classified as “bound” (defined as an SNR ≥ 5.000) have the SNR value highlighted in green in the tables, while mucins that were classified as unbound (defined as an SNR < 5.000) have the SNR value highlighted in red in the tables.

a)

				FAST Slide	NOVA Slide	FAST Slide	NOVA Slide	FAST Slide	NOVA Slide	FAST Slide	NOVA Slide
ChartID	PrintDetail	PrintConc	PrintID	Average RFU	Average RFU	STDEV	STDEV	%CV	%CV	Average SNR	Average SNR
1	Bovine Submaxillary Mucin (BSM)	1ug/ml	S-001	-101	-5	8	9	-8	-171	-0.432	-0.071
2	Bovine Submaxillary Mucin (BSM)	10ug/ml	S-002	-101	-2	19	16	-18	-723	-0.463	-0.003
3	Bovine Submaxillary Mucin (BSM)	100ug/ml	S-003	11	229	23	31	206	13	0.063	1.652
4	Porcine Gastric Mucin Type III (PGM)	1ug/ml	S-004	-100	-3	13	9	-13	-316	-0.529	-0.043
5	Porcine Gastric Mucin Type III (PGM)	10ug/ml	S-005	-75	-4	32	12	-43	-332	-0.347	-0.083
6	Porcine Gastric Mucin Type III (PGM)	100ug/ml	S-006	-130	22	17	28	-13	125	-0.560	-0.015
7	Human Normal Sputum Mucin	1ug/ml	S-007	332	570	186	207	56	36	1.381	2.056
8	Human Normal Sputum Mucin	10ug/ml	S-008	14626	10408	1112	717	8	7	64.694	47.984
9	Human Normal Sputum Mucin	100ug/ml	S-009	43172	36702	1447	2364	3	6	38.860	205.783
10	Human Cystic Fibrosis Sputum Mucin	1ug/ml	S-010	-85	16	27	16	-32	98	-0.349	-0.013
11	Human Cystic Fibrosis Sputum Mucin	10ug/ml	S-011	2596	2039	295	355	11	17	10.990	4.167
12	Human Cystic Fibrosis Sputum Mucin	100ug/ml	S-012	11476	8974	709	551	6	6	14.803	24.045
13	Bovine Serum Albumin (BSA)	1ug/ml	S-013	-128	21	13	67	-10	323	-0.496	-0.040
14	Bovine Serum Albumin (BSA)	10ug/ml	S-014	-117	6	25	18	-21	318	-0.529	-0.027
15	Bovine Serum Albumin (BSA)	100ug/ml	S-015	47	153	92	60	198	39	0.093	0.890
16	Phosphate Buffer	n/a	S-016	-157	-1	30	76	-19	-15186	-0.620	-0.157

b) Mock-Treated Neuraminidase

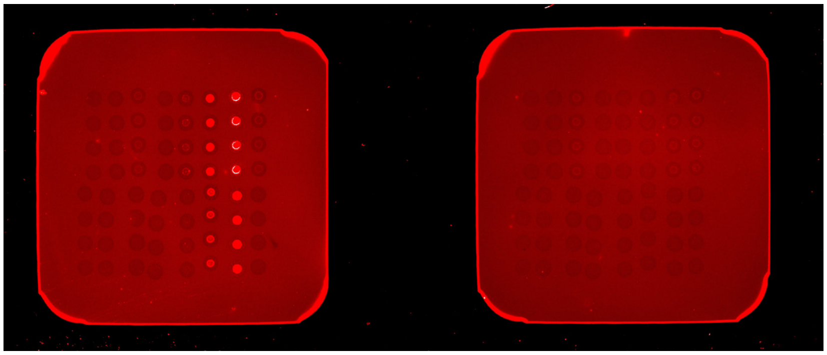

c) Mock-Treated Neuraminidase

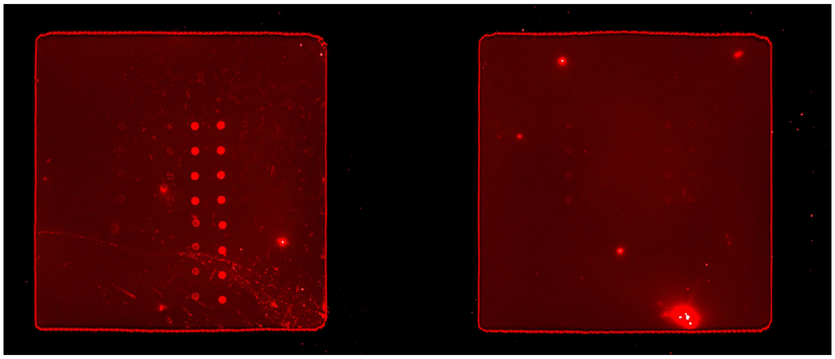

Supplementary Figure S9. Comparison of FAST vs. NOVA Nitrocellulose Slides for Mucin Microarray Printing. **A**. Side-by-side comparison of the average RFU, standard deviation (STDEV), %CV, and average SNR values for 0.5μg/ml CHO-131 screened on the FAST and NOVA nitrocellulose slide. The values shown represent the values measured from the mock-

treated subarrays shown in panels **B**) and **C**) of this figure. The higher values for the average RFU and average SNR and the lower value for the STDEV and %CV between the two slides are highlighted in green. Note that no biased trend is seen in the green coloring between the two slide types for the STDEV, %CV, or average SNR for glycoproteins bound (SNR ≥ 5.000) by CHO-131. **B**. Scanned microarray image of the 0.5μg/ml CHO-131 binding to the FAST nitrocellulose slide after mock treatment or treatment with neuraminidase. Fluorescence is pseudo-colored red for contrast purposes. **C**. Scanned microarray image of the 0.5μg/ml CHO-131 binding to the NOVA nitrocellulose slide after mock treatment or treatment with neuraminidase. Fluorescence is pseudo-colored red for contrast purposes. This image was scanned under similar PMT and laser power settings as the FAST slide, and the images are set to the same brightness and contrast settings.

a)

ChartID	Glycoprotein	1:1000 anti-H1 SNR (Super G)	1:1000 anti-H1 SNR (BSA)	1:100 anti-H1 SNR (Super G)	1:100 anti-H1 SNR (BSA)
2	Bovine Submaxillary Mucin_100ug/ml	6.561	2.702	10.635	11.013
4	Porcine Stomach Mucin, Type III_100ug/ml	62.226	26.217	232.517	72.039
6	Normal Human Sputum Mucin _100ug/ml	114.952	41.352	525.579	141.662
8	Human Cystic Fibrosis Sputum Mucin_100ug/ml	13.972	3.850	33.793	7.271
10	Glycophorin, Human_100ug/ml	3.602	1.263	36.264	10.984
12	alpha1-Acid Glycoprotein (Orosomucoid), from Human Serum_100ug/ml	-0.269	-0.322	-0.243	0.028
14	alpha1-Acid Glycoprotein (Orosomucoid), from Bovine Serum_100ug/ml	-0.382	0.065	0.093	-0.067
16	IgA, from Human Serum_100ug/ml	50.320	5.474	23.698	7.391
18	IgM, from Human Serum_100ug/ml	224.296	81.563	135.246	25.996
20	IgG, from Human Serum_100ug/ml	133.949	18.326	95.963	26.360
22	IgG, from Bovine Serum_100ug/ml	144.073	8.743	86.336	15.810
24	apo-Transferrin, Human_100ug/ml	18.971	5.837	7.256	4.518
26	a2-Macroglobulin, from Human Plasma_100ug/ml	21.920	7.209	26.002	9.893
28	alpha1-Antitrypsin, from Human Plasma_100ug/ml	4.197	3.157	2.120	4.365
30	Hemopexin, from Human Plasma_100ug/ml	5.404	7.332	3.197	4.986
32	Fibronectin, from Human Plasma_100ug/ml	84.691	3.183	585.623	113.608
34	Bovine Serum Albumin (BSA)_100ug/ml	124.345	2.665	77.028	4.131
36	Bovine Serum Albumin (BSA), Periodate-Treated_100ug/ml	157.839	5.204	111.077	46.934
38	alpha2-hs-Glycoprotein (Fetuin), Human_100ug/ml	53.840	-0.489	15.759	0.170
40	Fetuin, from Fetal Calf Serum_100ug/ml	39.061	0.348	5.683	0.378
42	Asialofetuin, from Fetal Calf Serum_100ug/ml	29.591	-0.076	4.623	1.327
44	Lactoferrin, from Human Milk_100ug/ml	40.631	69.999	40.762	32.746
46	Caseinoglycopeptide, from Bovine Casein_100ug/ml	0.091	-0.007	-0.167	0.771
48	Laminin, from Human Placenta_100ug/ml	70.550	13.312	41.193	11.172
50	Thyroglobulin, Human_100ug/ml	10.151	5.070	105.827	32.164
52	Thyroglobulin, from Bovine Thyroid_100ug/ml	29.877	4.141	138.871	16.580
54	Ovalbumin, from Chicken Egg White, Grade V_100ug/ml	46.261	2.611	95.463	23.028
56	Ovalbumin, from Chicken Egg White, Grade V, Periodate-Treated_100ug/ml	33.830	1.905	69.448	15.096
58	Trypsin Inhibitor (Ovomucoid), from Chicken Egg White_100ug/ml	1.628	0.428	0.579	0.173
60	Invertase, from Saccharomyces cerevisiae, Grade VII_100ug/ml	0.320	-0.181	0.421	0.393
62	RNase B, from Bovine Pancreas_100ug/ml	0.044	0.195	0.410	0.191
64	Horseradish Peroxidase (HRP)_100ug/ml	-0.068	-0.178	-0.025	0.000
66	Schistosoma mansoni Egg Antigen (SEA)_100ug/ml	48.104	43.916	78.964	10.798
68	Haptoglobin from Pooled Human Plasma_100ug/ml	14.264	2.851	20.717	2.983
70	sIgA from Human Colostrum_100ug/ml	189.399	67.366	183.658	24.619
72	Vitronectin, from Human Plasma_100ug/ml	16.369	1.914	26.557	0.523

Key:

	= expected binder based on previous demonstration of glycan determinant expression
	= dose-dependent binder, but no previous evidence of glycan determinant expression
	= dose-indepndent binder or known off-target binder
	= higher SNR for an expected binder
	= lower SNR for an expected binder
	= higher SNR for an off-target/dose-independent binder
	= lower SNR for an off-target/dose-independent binder
	= non-binder based on SNR < 5.000

b)

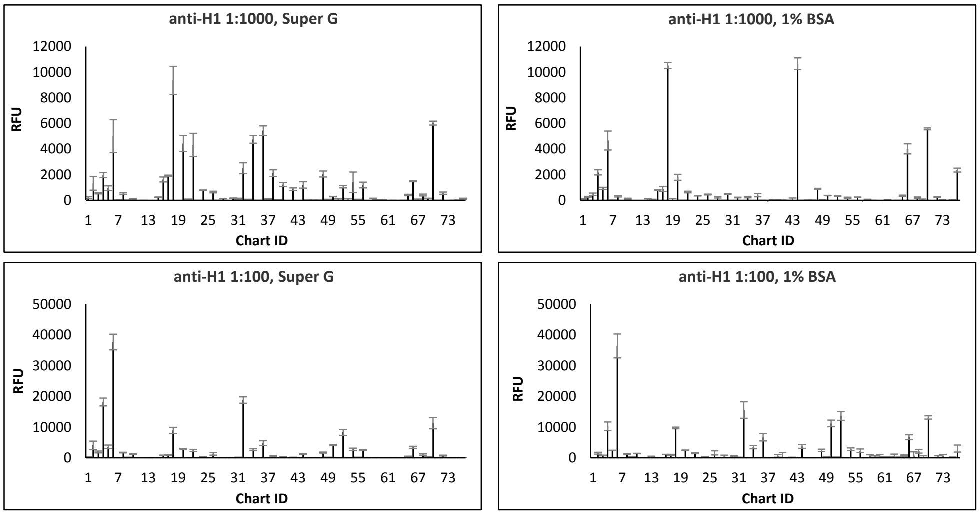

Supplementary Figure S10. Comparison of Super G vs. 1% BSA as Nitrocellulose Blocking Reagent. **A**. Comparison of SNRs for anti-H1 binding to the glycoprotein microarray v2 using Super G or 1% BSA as the blocking reagent. Only the 100µg/ml glycoproteins are shown in this figure. SNRs ≥ 5.000 are highlighted; the different highlight colors reflect whether the SNR is the higher or lower of two SNRs for Super G or 1% BSA as the blocking reagent as well as whether or not the glycoprotein itself was an expected binder/dose-dependent binder or an off-target binder/dose-independent binder. The SNRs for both the 1:1000 and 1:100 anti-H1 concentration screening are shown. **B**. Graphs showing the average RFU of 1:1000 (top graphs) and 1:100 (bottom graphs) anti-H1 binding to the glycoprotein microarray with Super G Blocking Buffer (left graphs) or 1% BSA (right graphs) used as the blocking reagent. Error bars represent the standard deviation.

a)

1μg/ml SNA		0.1% BSA	10% BSA
ChartID	PrintDetail	Average SNR	Average SNR
2	Bovine Submaxillary Mucin_100ug/ml	19.299	17.093
4	Porcine Stomach Mucin, Type III_100ug/ml	48.428	65.435
6	Normal Human Sputum Mucin _100ug/ml	87.831	165.233
8	Human Cystic Fibrosis Sputum Mucin_100ug/ml	32.441	15.793
10	Glycophorin, Human_100ug/ml	4.099	1.406
12	alpha1-Acid Glycoprotein (Orosomucoid), from Human Serum_100ug/ml	1.194	-0.031
14	alpha1-Acid Glycoprotein (Orosomucoid), from Bovine Serum_100ug/ml	2.199	0.160
16	IgA, from Human Serum_100ug/ml	28.047	2.702
18	IgM, from Human Serum_100ug/ml	72.612	36.108
20	IgG, from Human Serum_100ug/ml	23.792	24.897
22	IgG, from Bovine Serum_100ug/ml	76.933	27.643
24	apo-Transferrin, Human_100ug/ml	138.164	1.264
26	a2-Macroglobulin, from Human Plasma_100ug/ml	911.407	14.475
28	alpha1-Antitrypsin, from Human Plasma_100ug/ml	34.355	0.161
30	Hemopexin, from Human Plasma_100ug/ml	414.529	0.451
32	Fibronectin, from Human Plasma_100ug/ml	69.452	195.025
34	Bovine Serum Albumin (BSA)_100ug/ml	0.409	34.143
36	Bovine Serum Albumin (BSA), Periodate-Treated_100ug/ml	1.352	44.524
38	alpha2-hs-Glycoprotein (Fetuin), Human_100ug/ml	3.670	3.232
40	Fetuin, from Fetal Calf Serum_100ug/ml	11.350	1.118
42	Asialofetuin, from Fetal Calf Serum_100ug/ml	1.248	0.585
44	Lactoferrin, from Human Milk_100ug/ml	115.233	16.080
46	Caseinoglycopeptide, from Bovine Casein_100ug/ml	-0.335	0.107
48	Laminin, from Human Placenta_100ug/ml	30.657	14.806
50	Thyroglobulin, Human_100ug/ml	135.356	36.257
52	Thyroglobulin, from Bovine Thyroid_100ug/ml	298.208	62.166
54	Ovalbumin, from Chicken Egg White, Grade V_100ug/ml	-0.042	26.289
56	Ovalbumin, from Chicken Egg White, Grade V, Periodate-Treated_100ug/ml	0.501	23.761
58	Trypsin Inhibitor (Ovomucoid), from Chicken Egg White_100ug/ml	0.393	-0.087
60	Invertase, from Saccharomyces cerevisiae, Grade VII_100ug/ml	0.341	0.588
62	RNase B, from Bovine Pancreas_100ug/ml	0.118	0.150
64	Horseradish Peroxidase (HRP)_100ug/ml	-0.552	-0.098
66	Schistosoma mansoni Egg Antigen (SEA)_100ug/ml	73.085	27.938
68	Haptoglobin from Pooled Human Plasma_100ug/ml	140.840	12.036
70	sIgA from Human Colostrum_100ug/ml	267.813	37.066
72	Vitronectin, from Human Plasma_100ug/ml	6.490	0.314

Key:

	= expected binder based on previous demonstration of glycan determinant expression
	= dose-independent binder or known off-target binder
	= dose-dependent binder but no prior evidence of glycan determinant expression
	= binder (SNR ≥ 5.000)
	= non-binder (SNR < 5.000)

b)

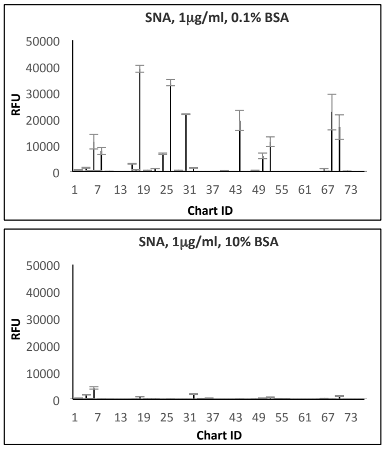

c)

CHO-131 (1μg/ml)		0.1% BSA	10% BSA
ChartID	PrintDetail	Average SNR	Average SNR
2	Bovine Submaxillary Mucin_100ug/ml	18.039	-0.156
4	Porcine Stomach Mucin, Type III_100ug/ml	0.088	-0.360
6	Normal Human Sputum Mucin _100ug/ml	82.866	56.993
8	Human Cystic Fibrosis Sputum Mucin_100ug/ml	24.536	37.005
10	Glycophorin, Human_100ug/ml	17.621	0.652
12	alpha1-Acid Glycoprotein (Orosomucoid), from Human Serum_100ug/ml	0.322	-0.183
14	alpha1-Acid Glycoprotein (Orosomucoid), from Bovine Serum_100ug/ml	0.107	-0.174
16	IgA, from Human Serum_100ug/ml	12.146	2.174
18	IgM, from Human Serum_100ug/ml	10.239	48.977
20	IgG, from Human Serum_100ug/ml	0.311	-0.026
22	IgG, from Bovine Serum_100ug/ml	5.119	0.412
24	apo-Transferrin, Human_100ug/ml	38.357	0.241
26	a2-Macroglobulin, from Human Plasma_100ug/ml	70.070	0.679
28	alpha1-Antitrypsin, from Human Plasma_100ug/ml	5.543	0.026
30	Hemopexin, from Human Plasma_100ug/ml	60.319	-0.233
32	Fibronectin, from Human Plasma_100ug/ml	144.247	0.112
34	Bovine Serum Albumin (BSA)_100ug/ml	65.806	0.840
36	Bovine Serum Albumin (BSA), Periodate-Treated_100ug/ml	38.355	0.673
38	alpha2-hs-Glycoprotein (Fetuin), Human_100ug/ml	2.131	-0.075
40	Fetuin, from Fetal Calf Serum_100ug/ml	9.008	-0.076
42	Asialofetuin, from Fetal Calf Serum_100ug/ml	2.289	-0.174
44	Lactoferrin, from Human Milk_100ug/ml	40.046	2.715
46	Caseinoglycopeptide, from Bovine Casein_100ug/ml	-0.156	-0.236
48	Laminin, from Human Placenta_100ug/ml	26.735	0.053
50	Thyroglobulin, Human_100ug/ml	110.864	0.314
52	Thyroglobulin, from Bovine Thyroid_100ug/ml	52.533	0.672
54	Ovalbumin, from Chicken Egg White, Grade V_100ug/ml	28.335	0.109
56	Ovalbumin, from Chicken Egg White, Grade V, Periodate-Treated_100ug/ml	21.938	0.034
58	Trypsin Inhibitor (Ovomucoid), from Chicken Egg White_100ug/ml	-0.480	-0.312
60	Invertase, from Saccharomyces cerevisiae, Grade VII_100ug/ml	0.418	-0.148
62	RNase B, from Bovine Pancreas_100ug/ml	-0.074	0.466
64	Horseradish Peroxidase (HRP)_100ug/ml	0.141	-0.064
66	Schistosoma mansoni Egg Antigen (SEA)_100ug/ml	42.779	3.817
68	Haptoglobin from Pooled Human Plasma_100ug/ml	34.227	0.698
70	sIgA from Human Colostrum_100ug/ml	23.941	3.153
72	Vitronectin, from Human Plasma_100ug/ml	32.111	-0.006

Key:

	= expected binder based on previous demonstration of glycan determinant expression
	= dose-independent binder or known off-target binder
	= bound by fluorescent probe alone
	= binder (SNR ≥ 5.000)
	= non-binder (SNR < 5.000)

d)

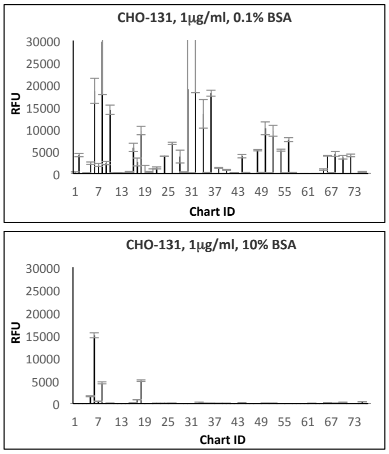

Supplementary Figure S11. BSA concentration empirically affects Glycan-Binding Protein binding Sensitivity and Specificity **A**. Comparison of the SNRs for SNA (1μg/ml) binding to

glycoprotein microarray v2 with 0.1% or 10% BSA in the CHO-131 binding buffer. SNRs ≥ 5.000, representing binding, are highlighted in green while SNRs < 5.000, representing non-binding, are highlighted in red. Yellow-green highlighting represents expected SNA binders (glycoproteins expressing known SNA binding determinants), yellow highlighting indicates glycoproteins that were bound by SNA in a dose-dependent manner but are not known to express SNA determinants, and pink highlighting represents off-target (non-glycan-specific) targets of SNA. The 100μg/ml but not 10μg/ml glycoproteins are included in this table. **B**. Graphs of 1μg/ml SNA binding to glycoprotein microarray v2 in the presence of either 0.1% BSA or 10% BSA in the SNA binding buffer. The graphs indicate the average RFU for each glycoprotein, and error bars represent the standard deviation. **C**. Comparison of the SNRs for CHO-131 (1μg/ml) binding to glycoprotein microarray v2 with 0.1% or 10% BSA in the CHO-131 binding buffer. SNRs ≥ 5.000, representing binding, are highlighted in green while SNRs < 5.000, representing non-binding, are highlighted in red. Yellow-green highlighting represents expected CHO-131 binders, lavender highlighting indicates glycoproteins that were bound by the goat anti-mouse IgM-Alexa Fluor 488 fluorescent probe (probe only) but not CHO-131, and pink highlighting represents off-target (non-glycan-specific) targets of CHO-131. The 100μg/ml but not 10μg/ml glycoproteins are included in this table. **D.** Graphs of 1μg/ml CHO-131 binding to glycoprotein microarray v2 in the presence of either 0.1% BSA or 10% BSA in the CHO-131 binding buffer. The graphs indicate the average RFU for each glycoprotein, and error bars represent the standard deviation.

Figure 3a. Thyroglobulin

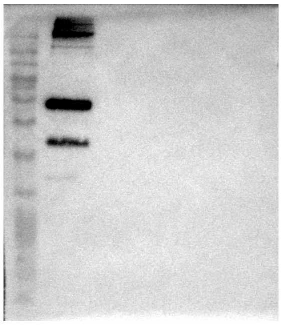

Figure 3a. Mucin and figure 5b asialofetuin

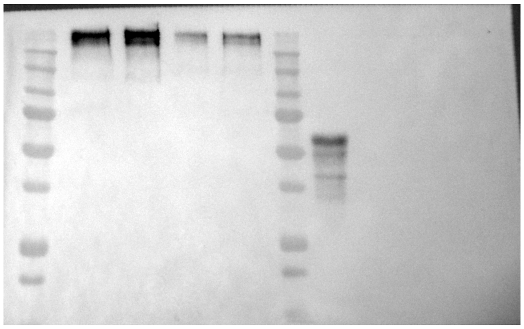

Figure 3b. Sulfatide

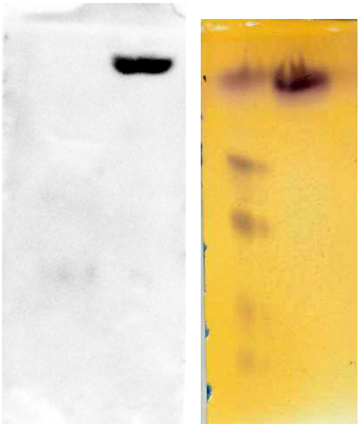

Figure 5a. Human plasma and purified human plasma glycoproteins

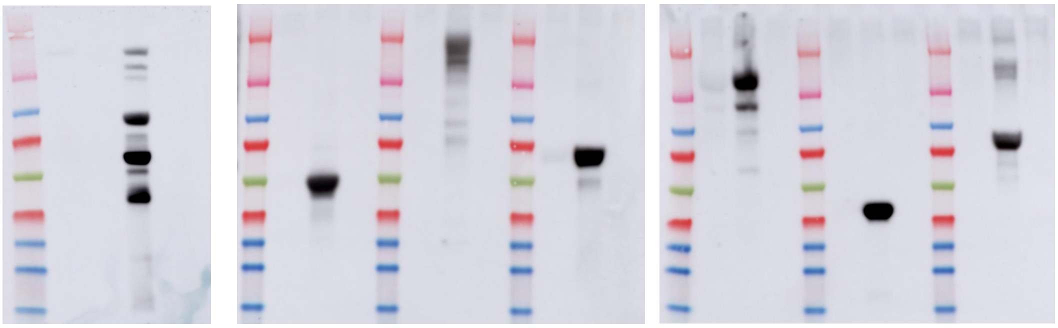

Supplementary Figure S12. Complete western blots and lipid blots for Figures 3 and 5. The orcinol stained lipid TLC plate is included to demonstrate the presence of the gangliosides and sulfatide lipids.

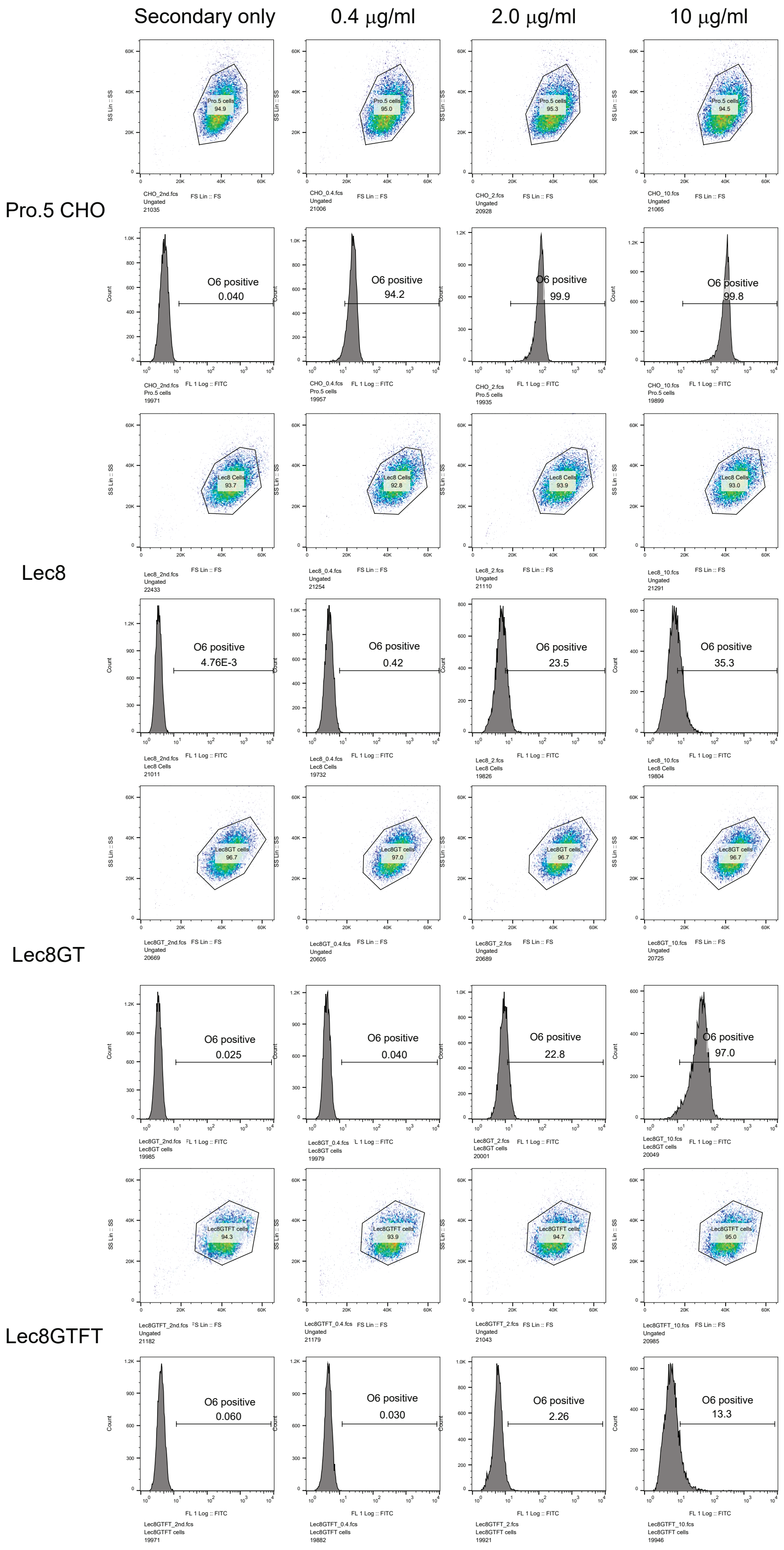

Supplementary Figure S13. Gating strategy of CHO cell lines stained with O6.

Supplementary References

1. Xia, B., et al., Altered O-glycosylation and sulfation of airway mucins associated with cystic fibrosis. *Glycobiology*, 2005. **15**(8): p. 747-75.
2. Shultz, M.A., et al., Optimized Blocking Of Porous Nitrocellulose Films For Sensitive Protein Microarrays. *Biotechniques*, 2013. **54**(4): p. 223-225.
3. Stillman, B.A. and J.L. Tonkinson, FAST slides: a novel surface for microarrays. *Biotechniques*, 2000. **29**(3): p. 630-5.
