# Supplementary material for: Novel lamprey antibody recognizes terminal sulfated galactose epitopes on mammalian glycoproteins
Source: Commun Biol. 2021 Jun 3;4:674. doi: 10.1038/s42003-021-02199-7 (PMC8175384; doi:10.1038/s42003-021-02199-7)
Supplement: Supplementary file 2 — Description of Additional Supplementary Files [file 42003_2021_2199_MOESM2_ESM.pdf]

Description of Additional Supplementary Files

File Name: Supplementary Data 1
Description: O6-yeast clone and O6-mFc binding to the CFG glycan microarray. O6-mFc tested at 2, 10, and 50 ug/ml.

File Name: Supplementary Data 2
Description: O6-mFc binding to the Glycosulfopeptide microarray.

File Name: Supplementary Data 3
Description: O6-mFc binding to the Glycoprotein microarray. O6-mFc tested at 0.1, 1, and 10 ug/ml.

File Name: Supplementary Data 4
Description: O6-mFc and lectin binding to the Glycosulfopeptide microarray, with and without PNGase F and Neuraminidase treatment of the array.

File Name: Supplementary Data 5
Description: Inventory and details of the Glycoproteins printed on the glycoprotein array.

File Name: Supplementary Data 6
Description: Optimization of the printed materials at multiple concentrations on 2 different array surfaces.

File Name: Supplementary Data 7
Description: Optimization of the printed materials at multiple concentrations tested with 2 concentrations of 2 lectins.

File Name: Supplementary Data 8
Description: Comparison of printing on FAST vs. NOVA slides.

File Name: Supplementary Data 9
Description: Analysis of binding of anti-H1 antibody with SuperG blocking buffer

File Name: Supplementary Data 10
Description: Analysis of binding of anti-H1 antibody with TSM blocking buffer.

File Name: Supplementary Data 11
Description: Analysis of lectin and antibody binding with varying amounts of BSA in the buffer.
