# Supplementary material for: Novel lamprey antibody recognizes terminal sulfated galactose epitopes on mammalian glycoproteins
Source: Commun Biol. 2021 Jun 3;4:674. doi: 10.1038/s42003-021-02199-7 (PMC8175384; doi:10.1038/s42003-021-02199-7)
Supplement: Supplementary file 14 — Reporting Summary [file 42003_2021_2199_MOESM14_ESM.pdf]

nature research

Corresponding author(s): *Double-blind peer review submissions: write DBPR and your manuscript number here instead of author names.*

Last updated by author(s): Mar 23, 2021

Reporting Summary

Nature Research wishes to improve the reproducibility of the work that we publish. This form provides structure for consistency and transparency in reporting. For further information on Nature Research policies, see our Editorial Policies and the Editorial Policy Checklist.

Statistics

For all statistical analyses, confirm that the following items are present in the figure legend, table legend, main text, or Methods section.

n/a	Confirmed	
x	☐	The exact sample size (*n*) for each experimental group/condition, given as a discrete number and unit of measurement
x	☐	A statement on whether measurements were taken from distinct samples or whether the same sample was measured repeatedly
x	☐	The statistical test(s) used AND whether they are one- or two-sided<br>*Only common tests should be described solely by name; describe more complex techniques in the Methods section.*
x	☐	A description of all covariates tested
x	☐	A description of any assumptions or corrections, such as tests of normality and adjustment for multiple comparisons
☐	x	A full description of the statistical parameters including central tendency (e.g. means) or other basic estimates (e.g. regression coefficient) AND variation (e.g. standard deviation) or associated estimates of uncertainty (e.g. confidence intervals)
x	☐	For null hypothesis testing, the test statistic (e.g. *F*, *t*, *r*) with confidence intervals, effect sizes, degrees of freedom and *P* value noted<br>*Give P values as exact values whenever suitable.*
x	☐	For Bayesian analysis, information on the choice of priors and Markov chain Monte Carlo settings
x	☐	For hierarchical and complex designs, identification of the appropriate level for tests and full reporting of outcomes
x	☐	Estimates of effect sizes (e.g. Cohen's *d*, Pearson's *r*), indicating how they were calculated

Our web collection on statistics for biologists contains articles on many of the points above.

Software and code

Policy information about availability of computer code

Data collection	GenePix 4300A, Stanford Synchrotron Radiation Lightsource (SSRL), Advanced Photon Source (APS)
Data analysis	GenePix 4300A, Origin 7.0, Phenix, HKL2000, XDS, Coot, FLowJo, DNASTAR Lasergene, Privateer

For manuscripts utilizing custom algorithms or software that are central to the research but not yet described in published literature, software must be made available to editors and reviewers. We strongly encourage code deposition in a community repository (e.g. GitHub). See the Nature Research guidelines for submitting code & software for further information.

Data

Policy information about availability of data

All manuscripts must include a data availability statement. This statement should provide the following information, where applicable:

- Accession codes, unique identifiers, or web links for publicly available datasets
- A list of figures that have associated raw data
- A description of any restrictions on data availability

All glycan and glycoprotein microarray data will be available for review at the National Center for Functional Glycomics (NCFG) website (https://ncfg.hms.harvard.edu). This data is represented in Figure 1A and Figure 4, and Supplementary data sets. DNA and amino acid sequences for O6 will be deposited into GenBank (BankIt2428777 O-6.seq MW699437) and crystal structures will be deposited into the RCSB protein data bank (PDB #7LA7, 7LA8). These data can be found in Figures 2, S1 and S2. There are no restrictions on any of the data found within this manuscript.

Field-specific reporting

Please select the one below that is the best fit for your research. If you are not sure, read the appropriate sections before making your selection.

- [x] Life sciences
- [ ] Behavioural & social sciences
- [ ] Ecological, evolutionary & environmental sciences

For a reference copy of the document with all sections, see nature.com/documents/nr-reporting-summary-flat.pdf

Life sciences study design

All studies must disclose on these points even when the disclosure is negative.

Sample size	N/A
Data exclusions	N/A
Replication	All glycans and glycoproteins printed on the microarrays are printed in replicates of four, to ensure reproducibility of binding
Randomization	N/A
Blinding	N/A

Reporting for specific materials, systems and methods

We require information from authors about some types of materials, experimental systems and methods used in many studies. Here, indicate whether each material, system or method listed is relevant to your study. If you are not sure if a list item applies to your research, read the appropriate section before selecting a response.

Materials & experimental systems

n/a	Involved in the study	
[ ]	[x]	Antibodies
[ ]	[x]	Eukaryotic cell lines
[x]	[ ]	Palaeontology and archaeology
[ ]	[x]	Animals and other organisms
[x]	[ ]	Human research participants
[x]	[ ]	Clinical data
[x]	[ ]	Dual use research of concern

Methods

n/a	Involved in the study	
[x]	[ ]	ChIP-seq
[ ]	[x]	Flow cytometry
[x]	[ ]	MRI-based neuroimaging

Antibodies

Antibodies used

EMD Millipore, Anti-Myc Antibody, Alexa Fluor 488, Clone 4A6, catalog # 16-224
ThermoFisher, Streptavidin, PE-Cy5.5, catalog # SA1018
Dr. Max Cooper's laboratory, Mouse anti-VLRB, Clone 4C4
ThermoFisher Molecular Probes, Goat anti-Mouse IgG (H+L), Alexa Fluor 488, Catalog #A-11001
ThermoFisher Molecular Probes, Goat anti-Mouse IgG (H+L), Alexa Fluor 633, Catalog #A-21050
Southern Biotech, Goat Anti-Mouse IgG Fc-HRP, Catalog # 1033-05
Sigma-Aldrich, Mouse Anti-HRP antibody, clone 3A5C6, Catalog # SAB5300168

Validation

Validation of the secondary antibodies was performed by the companies, and the data was validated on their website. Secondary only controls were performed to eliminate non-specific binding of the reagents for all experiments. Mouse monoclonal antibody 4C4 has been used and validated in previous publications (reference #29, Alder et al 2005)

Eukaryotic cell lines

Policy information about cell lines

Cell line source(s)

Saccharomyces cerevisiae Meyen ex E.C. Hansen ATCC: MYA-4941
Human cells: HEK-293F (Freestyle 293F) ThemoFisher Cat# R79007
Chinese Hamster Ovary cells: CHO (Pro-5) ATCC (CRL-1781)
Chinese Hamster Ovary cells: CHO (Lec8) ATCC (CRL-1737)
Chinese Hamster Ovary cells: Lec8GT and Lec8GTFT cells were made in the Cummings laboratory and are described in reference #52, Prasanphanich et al 2014

Authentication	The cell lines were not authenticated further
Mycoplasma contamination	The cell lines were not tested for mycoplasma
Commonly misidentified lines (See ICLAC register)	N/A

Animals and other organisms

Policy information about studies involving animals; ARRIVE guidelines recommended for reporting animal research

Laboratory animals	Petromyzon marinus
Wild animals	N/A
Field-collected samples	N/A
Ethics oversight	Lamprey husbandry and immunizations protocols were approved by the institutional animal care and use committees (IACUC) at Emory University.

Note that full information on the approval of the study protocol must also be provided in the manuscript.

Flow Cytometry

Plots

Confirm that:

- [x] The axis labels state the marker and fluorochrome used (e.g. CD4-FITC).
- [x] The axis scales are clearly visible. Include numbers along axes only for bottom left plot of group (a 'group' is an analysis of identical markers).
- [x] All plots are contour plots with outliers or pseudocolor plots.
- [x] A numerical value for number of cells or percentage (with statistics) is provided.

Methodology

Sample preparation	As described in the manuscript, adherent cells were collected using Trypsin with 0.05% EDTA and fixed in 4% PFA. Cells were then incubated with 0.4, 2 and 10 μg/mL of O6-mFc in PBS with 1% BSA for 1 hr at 4°C. Cells were washed with PBS-tween four times, and then incubated with an anti-mouse IgG-488 secondary reagent in PBS with 1% BSA for 1 hr at 4°C. A secondary only incubation was used as a negative control.
Instrument	BD Accuri
Software	FlowJo 9.0
Cell population abundance	As these are cell lines, ~90% of the total events (~20,000) were used to determine antibody staining
Gating strategy	Cells were gated by forward and side scatter. As the cells were fixed in 4% PFA, there was no need for a live dead marker. O6 positive cells were identified by gating parameters on secondary only controls as shown in supplementary figure 13

- [x] Tick this box to confirm that a figure exemplifying the gating strategy is provided in the Supplementary Information.
