# Supplementary material for: Novel lamprey antibody recognizes terminal sulfated galactose epitopes on mammalian glycoproteins
Source: Commun Biol. 2021 Jun 3;4:674. doi: 10.1038/s42003-021-02199-7 (PMC8175384; doi:10.1038/s42003-021-02199-7)
Supplement: Supplementary file 15 — Validation Report [file 42003_2021_2199_MOESM15_ESM.pdf]

Preliminary Full wwPDB X-ray Structure Validation Report (i)

Mar 5, 2021 – 01:15 AM EST

Deposition ID : D_1000253841

This is a Preliminary Full wwPDB X-ray Structure Validation Report.

This report is produced by the wwPDB Deposition System during initial deposition but before annotation of the structure.

We welcome your comments at *validation@mail.wwpdb.org*
A user guide is available at
https://www.wwpdb.org/validation/2017/XrayValidationReportHelp
with specific help available everywhere you see the (i) symbol.

PRELIMINARY VALIDATION REPORT

The following versions of software and data (see references (i)) were used in the production of this report:

MolProbity	:	4.02b-467
Mogul	:	1.8.5 (274361), CSD as541be (2020)
Xtriage (Phenix)	:	1.13
EDS	:	2.17.1
buster-report	:	1.1.7 (2018)
Percentile statistics	:	20191225.v01 (using entries in the PDB archive December 25th 2019)
Refmac	:	5.8.0158
CCP4	:	7.0.044 (Gargrove)
Ideal geometry (proteins)	:	Engh & Huber (2001)
Ideal geometry (DNA, RNA)	:	Parkinson et al. (1996)
Validation Pipeline (wwPDB-VP)	:	2.17.1

1 Overall quality at a glance (i)

The following experimental techniques were used to determine the structure:
X-RAY DIFFRACTION

The reported resolution of this entry is 1.90 Å.

Percentile scores (ranging between 0-100) for global validation metrics of the entry are shown in the following graphic. The table shows the number of entries on which the scores are based.

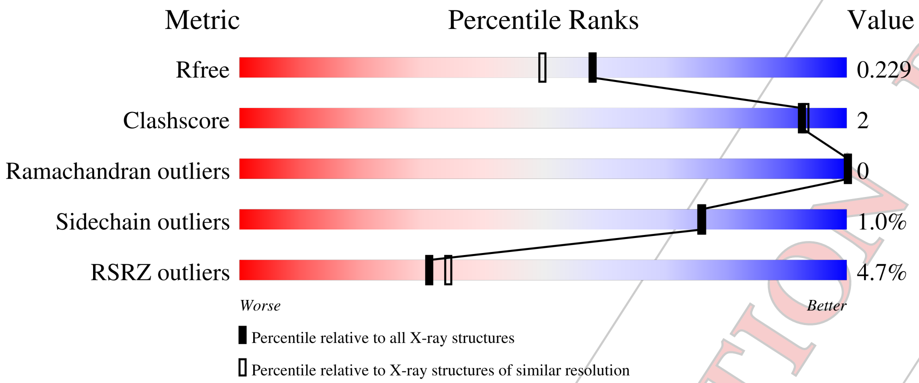

Metric	Whole archive (#Entries)	Similar resolution (#Entries, resolution range(Å))
R_{free}	130704	6207 (1.90-1.90)
Clashscore	141614	6847 (1.90-1.90)
Ramachandran outliers	138981	6760 (1.90-1.90)
Sidechain outliers	138945	6760 (1.90-1.90)
RSRZ outliers	127900	6082 (1.90-1.90)

The table below summarises the geometric issues observed across the polymeric chains and their fit to the electron density. The red, orange, yellow and green segments of the lower bar indicate the fraction of residues that contain outliers for >=3, 2, 1 and 0 types of geometric quality criteria respectively. A grey segment represents the fraction of residues that are not modelled. The numeric value for each fraction is indicated below the corresponding segment, with a dot representing fractions <=5% The upper red bar (where present) indicates the fraction of residues that have poor fit to the electron density. The numeric value is given above the bar.

Mol	Chain	Length	Quality of chain
1	A	173	6% / 97% • •
1	B	173	3% / 94% 6% •
2	C	2	50% 50%
2	D	2	50% 50%

PRELIMINARY VALIDATION REPORT

2 Entry composition (i)

There are 5 unique types of molecules in this entry. The entry contains 5487 atoms, of which 2586 are hydrogens and 0 are deuteriums.

In the tables below, the ZeroOcc column contains the number of atoms modelled with zero occupancy, the AltConf column contains the number of residues with at least one atom in alternate conformation and the Trace column contains the number of residues modelled with at most 2 atoms.

- Molecule 1 is a protein called O6 variable lymphocyte receptor.

Mol	Chain	Residues	Atoms	ZeroOcc	AltConf	Trace
1	A	172	Total C H N O S<br>2556 814 1258 222 254 8	0	0	0
1	B	172	Total C H N O S<br>2542 811 1250 219 254 8	0	0	0

- Molecule 2 is an oligosaccharide called 3-O-sulfo-beta-D-galactopyranose-(1-4)-2-acetamido-2-deoxy-beta-D-glucopyranose.

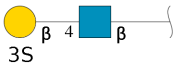

Mol	Chain	Residues	Atoms	ZeroOcc	AltConf	Trace
2	C	2	Total C H N O S<br>54 14 24 1 14 1	0	0	0
2	D	2	Total C H N O S<br>54 14 24 1 14 1	0	0	0

- Molecule 3 is BIOTIN (three-letter code: SGA) (formula: $C_6H_{12}O_9S$).

PRELIMINARY VALIDATION REPORT

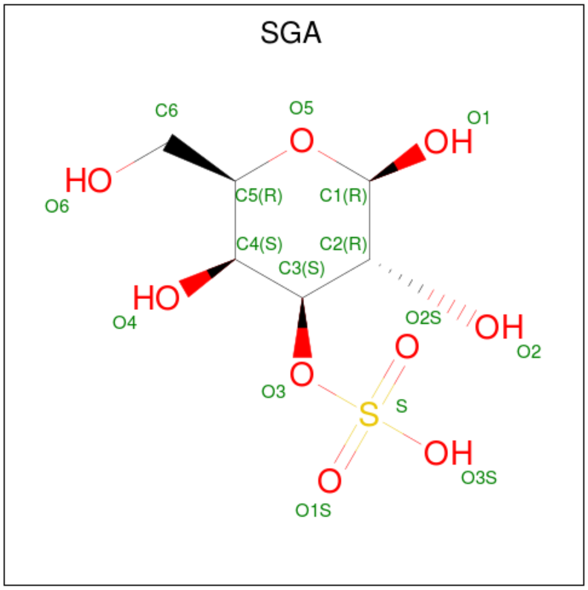

Mol	Chain	Residues	Atoms	ZeroOcc	AltConf
3	D	1	Total C H N O S<br>31 10 15 2 3 1	0	0
3	D	1	Total C H N O S<br>31 10 15 2 3 1	0	0

- Molecule 4 is SULFATE ION (three-letter code: SO4) (formula: O_4S).

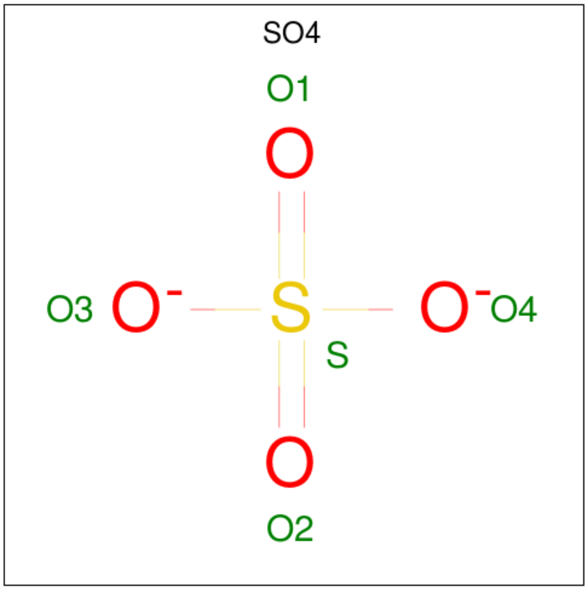

Mol	Chain	Residues	Atoms	ZeroOcc	AltConf
4	F	1	Total O S<br>5 4 1	0	0
4	F	1	Total O S<br>5 4 1	0	0

Continued on next page...

PRELIMINARY VALIDATION REPORT

Continued from previous page...

Mol	Chain	Residues	Atoms	ZeroOcc	AltConf
4	F	1	Total O S<br>5 4 1	0	0
4	F	1	Total O S<br>5 4 1	0	0

- Molecule 5 is water.

Mol	Chain	Residues	Atoms	ZeroOcc	AltConf
5	S	199	Total O<br>199 199	0	0

PRELIMINARY VALIDATION REPORT

3 Residue-property plots (i)

These plots are drawn for all protein, RNA, DNA and oligosaccharide chains in the entry. The first graphic for a chain summarises the proportions of the various outlier classes displayed in the second graphic. The second graphic shows the sequence view annotated by issues in geometry and electron density. Residues are color-coded according to the number of geometric quality criteria for which they contain at least one outlier: green = 0, yellow = 1, orange = 2 and red = 3 or more. A red dot above a residue indicates a poor fit to the electron density (RSRZ > 2). Stretches of 2 or more consecutive residues without any outlier are shown as a green connector. Residues present in the sample, but not in the model, are shown in grey.

- Molecule 1: O6 variable lymphocyte receptor

Chain A:
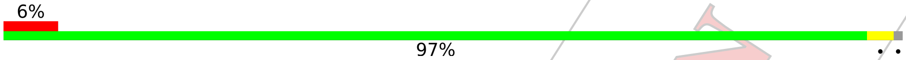

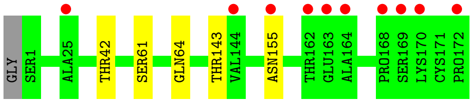

- Molecule 1: O6 variable lymphocyte receptor

Chain B:
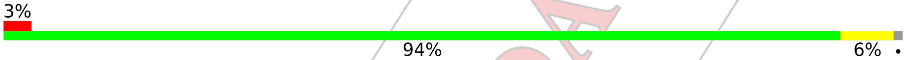

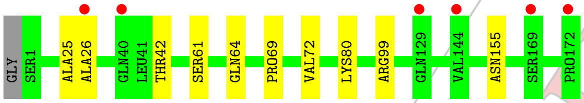

- Molecule 2: 3-O-sulfo-beta-D-galactopyranose-(1-4)-2-acetamido-2-deoxy-beta-D-glucopyranos e

Chain C:
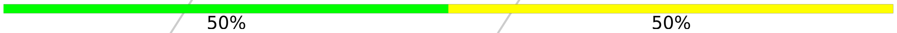

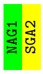

- Molecule 2: 3-O-sulfo-beta-D-galactopyranose-(1-4)-2-acetamido-2-deoxy-beta-D-glucopyranos e

Chain D:
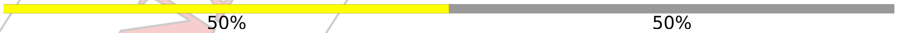

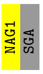

PRELIMINARY VALIDATION REPORT

4 Data and refinement statistics (i)

Property	Value	Source
Space group	I 4	Depositor
Cell constants<br>a, b, c, α, β, γ	107.25Å 107.25Å 63.20Å<br>90.00° 90.00° 90.00°	Depositor
Resolution (Å)	37.92 – 1.90<br>37.92 – 1.90	Depositor<br>EDS
% Data completeness<br>(in resolution range)	99.8 (37.92-1.90)<br>99.8 (37.92-1.90)	Depositor<br>EDS
R_{merge}	(Not available)	Depositor
R_{sym}	0.11	Depositor
$< I/\sigma(I) >$ [1]	1.23 (at 1.89Å)	Xtriage
Refinement program	phenix.refine 1.11_2567, PHENIX 1.11_2567	Depositor
R, R_{free}	0.184 , 0.229<br>0.184 , 0.229	Depositor<br>DCC
R_{free} test set	1421 reflections (4.99%)	wwPDB-VP
Wilson B-factor (Å^2)	29.3	Xtriage
Anisotropy	0.401	Xtriage
Bulk solvent k_{sol}(e/Å^3), B_{sol}(Å^2)	0.45 , 52.4	EDS
L-test for twinning[2]	$< \|L\| >$ = 0.49, $< L^2 >$ = 0.32	Xtriage
Estimated twinning fraction	0.025 for -k,-h,-l	Xtriage
F_o,F_c correlation	0.96	EDS
Total number of atoms	5487	wwPDB-VP
Average B, all atoms (Å^2)	34.0	wwPDB-VP

Xtriage's analysis on translational NCS is as follows: *The largest off-origin peak in the Patterson function is 5.56% of the height of the origin peak. No significant pseudotranslation is detected.*

[1] Intensities estimated from amplitudes.

[2] Theoretical values of $< |L| >$, $< L^2 >$ for acentric reflections are 0.5, 0.333 respectively for untwinned datasets, and 0.375, 0.2 for perfectly twinned datasets.

PRELIMINARY VALIDATION REPORT

5 Model quality (i)

5.1 Standard geometry (i)

Bond lengths and bond angles in the following residue types are not validated in this section: SO4, NAG, SGA, BTN

The Z score for a bond length (or angle) is the number of standard deviations the observed value is removed from the expected value. A bond length (or angle) with $|Z| > 5$ is considered an outlier worth inspection. RMSZ is the root-mean-square of all Z scores of the bond lengths (or angles).

Mol	Chain	Bond lengths		Bond angles	
		RMSZ	$\#\|Z\| > 5$	RMSZ	$\#\|Z\| > 5$
1	A	0.66	0/1330	0.74	0/1826
1	B	0.70	0/1324	0.77	1/1819 (0.1%)
All	All	0.68	0/2654	0.75	1/3645 (0.0%)

There are no bond length outliers.

All (1) bond angle outliers are listed below:

Mol	Chain	Res	Type	Atoms	Z	Observed(^{o})	Ideal(^{o})
1	B	99	ARG	NE-CZ-NH1	-5.80	117.40	120.30

There are no chirality outliers.

There are no planarity outliers.

5.2 Too-close contacts (i)

In the following table, the Non-H and H(model) columns list the number of non-hydrogen atoms and hydrogen atoms in the chain respectively. The H(added) column lists the number of hydrogen atoms added and optimized by MolProbity. The Clashes column lists the number of clashes within the asymmetric unit, whereas Symm-Clashes lists symmetry-related clashes.

Mol	Chain	Non-H	H(model)	H(added)	Clashes	Symm-Clashes
1	A	1298	1258	1258	2	2
1	B	1292	1250	1250	6	0
2	C	30	24	24	0	0
2	D	30	24	14	0	0
3	D	32	30	40	0	0
4	F	20	0	0	1	0
5	S	199	0	0	3	2
All	All	2901	2586	2586	8	2

PRELIMINARY VALIDATION REPORT

The all-atom clashscore is defined as the number of clashes found per 1000 atoms (including hydrogen atoms). The all-atom clashscore for this structure is 2.

All (8) close contacts within the same asymmetric unit are listed below, sorted by their clash magnitude.

Atom-1	Atom-2	Interatomic distance (Å)	Clash overlap (Å)
4:F:4:SO4:O2	5:S:147:HOH:O	1.85	0.93
1:B:155:ASN:ND2	5:S:81:HOH:O	2.19	0.74
1:A:42:THR:HG21	1:B:42:THR:HG21	1.75	0.68
1:B:80:LYS:NZ	5:S:108:HOH:O	2.27	0.67
1:B:42:THR:HG22	1:B:64:GLN:HB2	1.93	0.50
1:B:25:ALA:O	1:B:26:ALA:HB3	2.17	0.44
1:A:42:THR:HG22	1:A:64:GLN:HB2	2.02	0.42
1:B:69:PRO:HG2	1:B:72:VAL:HB	2.03	0.40

All (2) symmetry-related close contacts are listed below. The label for Atom-2 includes the symmetry operator and encoded unit-cell translations to be applied.

Atom-1	Atom-2	Interatomic distance (Å)	Clash overlap (Å)
1:A:155:ASN:ND2	5:S:39:HOH:O[3_555]	2.01	0.19
1:A:155:ASN:HD21	5:S:39:HOH:O[3_555]	1.46	0.14

5.3 Torsion angles (i)

5.3.1 Protein backbone (i)

In the following table, the Percentiles column shows the percent Ramachandran outliers of the chain as a percentile score with respect to all X-ray entries followed by that with respect to entries of similar resolution.

The Analysed column shows the number of residues for which the backbone conformation was analysed, and the total number of residues.

Mol	Chain	Analysed	Favoured	Allowed	Outliers	Percentiles	
1	A	170/173 (98%)	162 (95%)	8 (5%)	0	100	100
1	B	170/173 (98%)	161 (95%)	9 (5%)	0	100	100
All	All	340/346 (98%)	323 (95%)	17 (5%)	0	100	100

There are no Ramachandran outliers to report.

PRELIMINARY VALIDATION REPORT

5.3.2 Protein sidechains (i)

In the following table, the Percentiles column shows the percent sidechain outliers of the chain as a percentile score with respect to all X-ray entries followed by that with respect to entries of similar resolution.

The Analysed column shows the number of residues for which the sidechain conformation was analysed, and the total number of residues.

Mol	Chain	Analysed	Rotameric	Outliers	Percentiles	
1	A	148/150 (99%)	146 (99%)	2 (1%)	67	65
1	B	147/150 (98%)	146 (99%)	1 (1%)	84	84
All	All	295/300 (98%)	292 (99%)	3 (1%)	76	76

All (3) residues with a non-rotameric sidechain are listed below:

Mol	Chain	Res	Type
1	A	61	SER
1	A	143	THR
1	B	61	SER

Sometimes sidechains can be flipped to improve hydrogen bonding and reduce clashes. There are no such sidechains identified.

5.3.3 RNA (i)

There are no RNA molecules in this entry.

5.4 Non-standard residues in protein, DNA, RNA chains (i)

There are no non-standard protein/DNA/RNA residues in this entry.

5.5 Carbohydrates (i)

5 monosaccharides are modelled in this entry.

In the following table, the Counts columns list the number of bonds (or angles) for which Mogul statistics could be retrieved, the number of bonds (or angles) that are observed in the model and the number of bonds (or angles) that are defined in the Chemical Component Dictionary. The Link column lists molecule types, if any, to which the group is linked. The Z score for a bond length (or angle) is the number of standard deviations the observed value is removed from the expected value. A bond length (or angle) with $|Z| > 2$ is considered an outlier worth inspection. RMSZ is the root-mean-square of all Z scores of the bond lengths (or angles).

PRELIMINARY VALIDATION REPORT

Mol	Type	Chain	Res	Link	Bond lengths			Bond angles		
					Counts	RMSZ	#|Z| > 2	Counts	RMSZ	#|Z| > 2
2	NAG	C	1	2	15,15,15	0.34	0	21,21,21	0.85	0
2	SGA	C	2	2	15,15,16	1.50	2 (13%)	17,22,24	1.62	3 (17%)
2	NAG	D	1	3	15,15,15	1.03	2 (13%)	21,21,21	1.14	1 (4%)
3	BTN	D	2	-	14,17,17	3.23	2 (14%)	19,23,23	1.11	2 (10%)
3	SGA	D	2	2	15,15,16	1.22	2 (13%)	17,22,24	1.42	3 (17%)

In the following table, the Chirals column lists the number of chiral outliers, the number of chiral centers analysed, the number of these observed in the model and the number defined in the Chemical Component Dictionary. Similar counts are reported in the Torsion and Rings columns. '-' means no outliers of that kind were identified.

Mol	Type	Chain	Res	Link	Chirals	Torsions	Rings
2	NAG	C	1	2	-	0/6/26/26	0/1/1/1
2	SGA	C	2	2	-	2/7/24/27	0/1/1/1
2	NAG	D	1	3	-	0/6/26/26	0/1/1/1
3	BTN	D	2	-	-	1/5/28/28	0/2/2/2
3	SGA	D	2	2	-	1/7/24/27	0/1/1/1

All (8) bond length outliers are listed below:

Mol	Chain	Res	Type	Atoms	Z	Observed(Å)	Ideal(Å)
3	D	2	BTN	O3-C3	8.54	1.41	1.23
3	D	2	BTN	C2-S1	-8.06	1.69	1.82
2	C	2	SGA	O5-C1	3.25	1.48	1.43
2	C	2	SGA	O3-S	2.70	1.65	1.57
2	D	1	NAG	O5-C1	-2.70	1.36	1.42
3	D	2	SGA	O3-S	2.69	1.65	1.57
2	D	1	NAG	C1-C2	-2.59	1.49	1.52
3	D	2	SGA	O5-C1	2.15	1.47	1.43

All (9) bond angle outliers are listed below:

Mol	Chain	Res	Type	Atoms	Z	Observed(^{o})	Ideal(^{o})
2	C	2	SGA	C2-C3-C4	-3.50	107.61	110.66
2	D	1	NAG	O1-C1-C2	-3.46	102.03	109.22
3	D	2	SGA	C1-C2-C3	3.33	113.19	109.17
3	D	2	SGA	O2S-S-O1S	-2.54	102.01	112.22
2	C	2	SGA	O2S-S-O1S	-2.53	102.05	112.22
2	C	2	SGA	O3S-S-O1S	2.46	117.03	108.49
3	D	2	BTN	C4-C2-S1	2.38	107.47	105.20
3	D	2	SGA	O3S-S-O2S	2.28	116.42	108.49

Continued on next page...

PRELIMINARY VALIDATION REPORT

Continued from previous page...

Mol	Chain	Res	Type	Atoms	Z	Observed(°)	Ideal(°)
3	D	2	BTN	C2-C4-N2	-2.24	111.12	113.13

There are no chirality outliers.

All (4) torsion outliers are listed below:

Mol	Chain	Res	Type	Atoms
2	C	2	SGA	C4-C5-C6-O6
2	C	2	SGA	O5-C5-C6-O6
3	D	2	BTN	C7-C8-C9-C10
3	D	2	SGA	O5-C5-C6-O6

There are no ring outliers.

No monomer is involved in short contacts.

The following is a two-dimensional graphical depiction of Mogul quality analysis of bond lengths, bond angles, torsion angles, and ring geometry for oligosaccharide.

Oligosaccharide Chain C

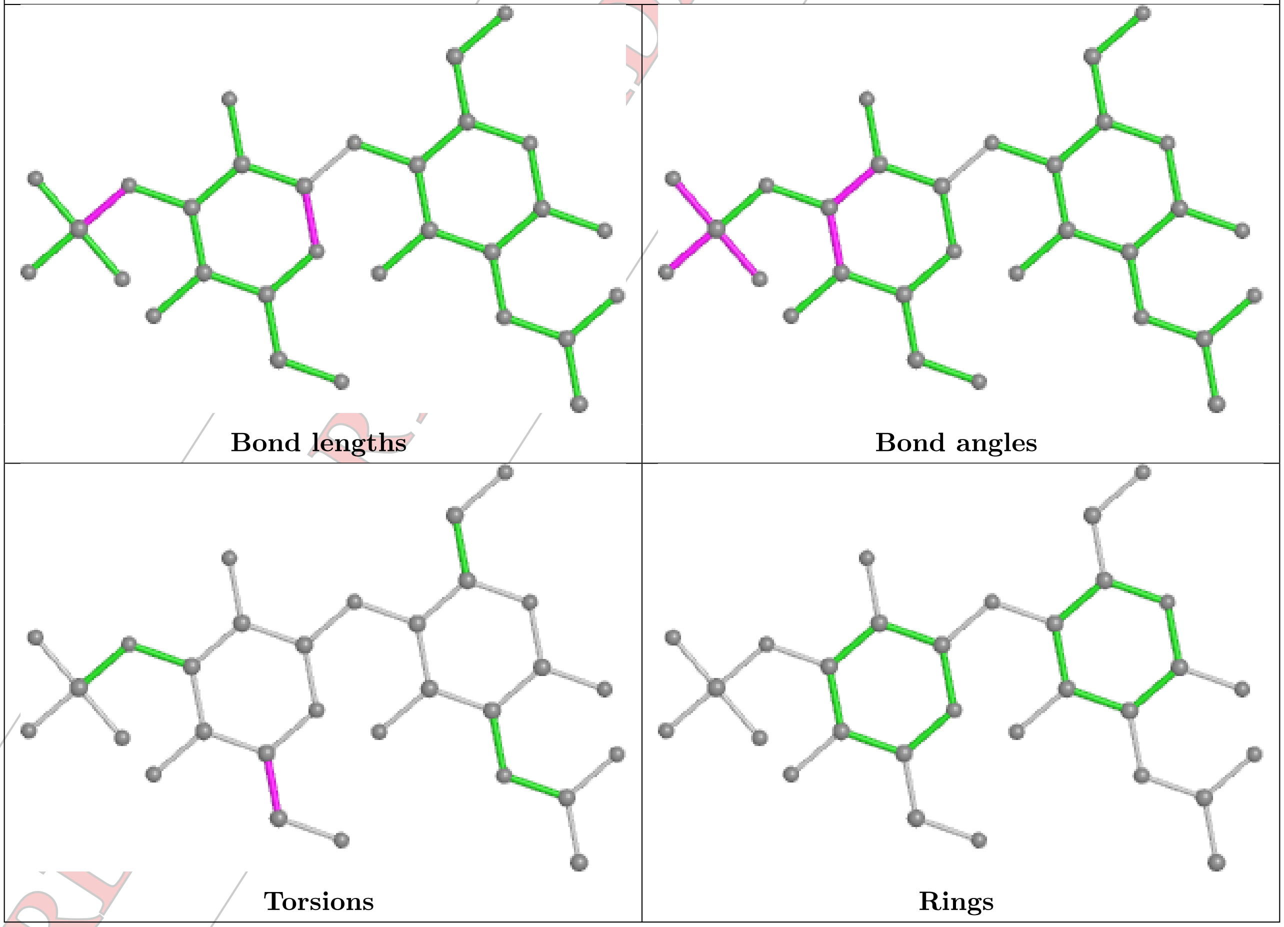

PRELIMINARY VALIDATION REPORT

Oligosaccharide Chain D

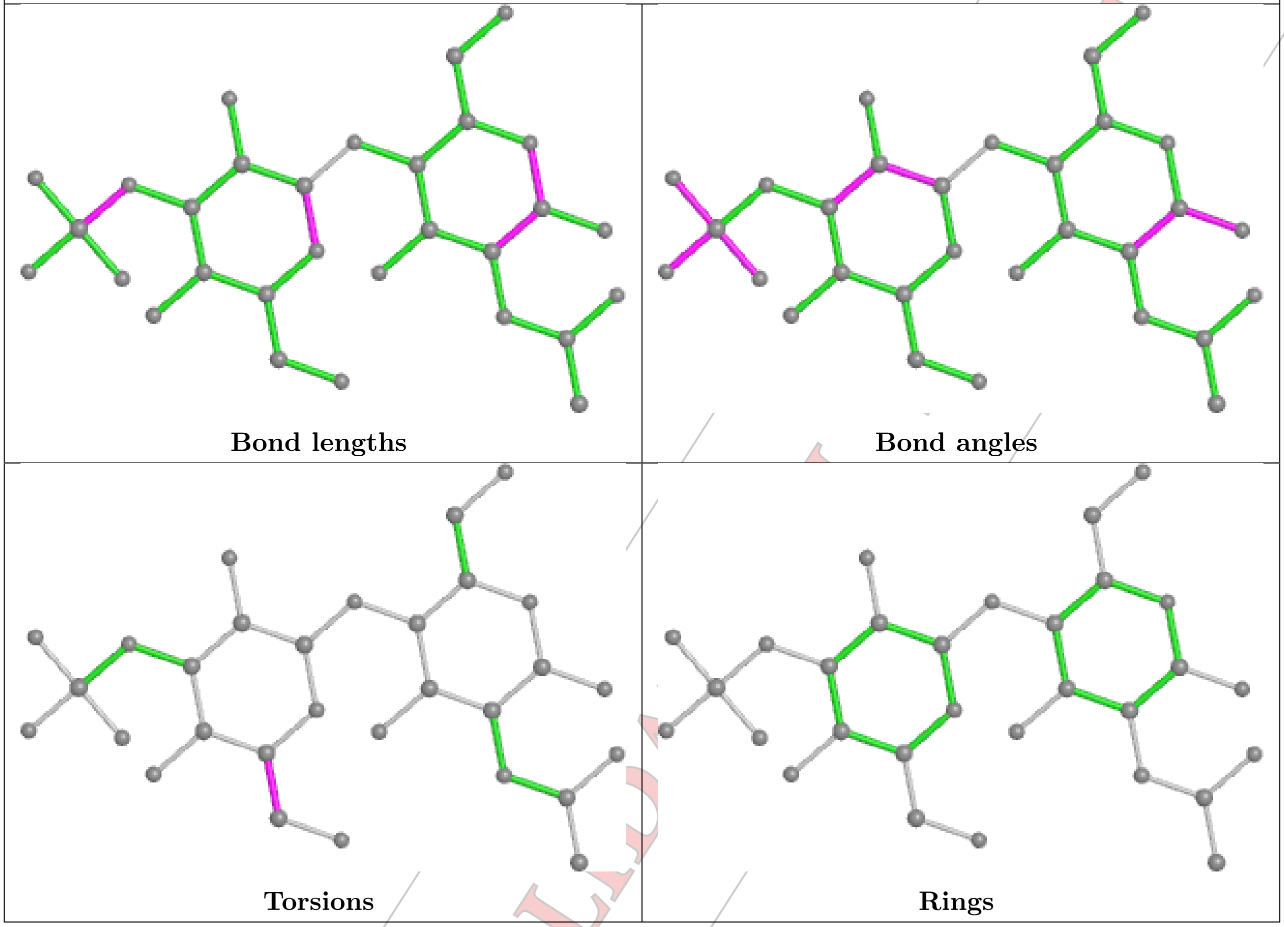

5.6 Ligand geometry ⓘ

7 ligands are modelled in this entry.

In the following table, the Counts columns list the number of bonds (or angles) for which Mogul statistics could be retrieved, the number of bonds (or angles) that are observed in the model and the number of bonds (or angles) that are defined in the Chemical Component Dictionary. The Link column lists molecule types, if any, to which the group is linked. The Z score for a bond length (or angle) is the number of standard deviations the observed value is removed from the expected value. A bond length (or angle) with $|Z| > 2$ is considered an outlier worth inspection. RMSZ is the root-mean-square of all Z scores of the bond lengths (or angles).

Mol	Type	Chain	Res	Link	Bond lengths			Bond angles		
					Counts	RMSZ	$\#\|Z\| > 2$	Counts	RMSZ	$\#\|Z\| > 2$
3	BTN	D	2	-	14,17,17	3.23	2 (14%)	19,23,23	1.11	2 (10%)
4	SO4	F	4	-	4,4,4	0.15	0	6,6,6	0.26	0
3	BTN	D	3	-	14,17,17	3.21	2 (14%)	19,23,23	1.12	1 (5%)
4	SO4	F	1	-	4,4,4	0.16	0	6,6,6	0.21	0
3	SGA	D	2	2	15,15,16	1.22	2 (13%)	17,22,24	1.42	3 (17%)
4	SO4	F	3	-	4,4,4	0.13	0	6,6,6	0.16	0

Mol	Type	Chain	Res	Link	Bond lengths			Bond angles		
					Counts	RMSZ	#|Z| > 2	Counts	RMSZ	#|Z| > 2
4	SO4	F	2	-	4,4,4	0.15	0	6,6,6	0.19	0

In the following table, the Chirals column lists the number of chiral outliers, the number of chiral centers analysed, the number of these observed in the model and the number defined in the Chemical Component Dictionary. Similar counts are reported in the Torsion and Rings columns. '-' means no outliers of that kind were identified.

Mol	Type	Chain	Res	Link	Chirals	Torsions	Rings
3	SGA	D	2	2	-	1/7/24/27	0/1/1/1
3	BTN	D	3	-	-	1/5/28/28	0/2/2/2
3	BTN	D	2	-	-	1/5/28/28	0/2/2/2

All (6) bond length outliers are listed below:

Mol	Chain	Res	Type	Atoms	Z	Observed(Å)	Ideal(Å)
3	D	2	BTN	O3-C3	8.54	1.41	1.23
3	D	3	BTN	O3-C3	8.50	1.41	1.23
3	D	2	BTN	C2-S1	-8.06	1.69	1.82
3	D	3	BTN	C2-S1	-7.94	1.69	1.82
3	D	2	SGA	O3-S	2.69	1.65	1.57
3	D	2	SGA	O5-C1	2.15	1.47	1.43

All (6) bond angle outliers are listed below:

Mol	Chain	Res	Type	Atoms	Z	Observed(^{o})	Ideal(^{o})
3	D	2	SGA	C1-C2-C3	3.33	113.19	109.17
3	D	2	SGA	O2S-S-O1S	-2.54	102.01	112.22
3	D	2	BTN	C4-C2-S1	2.38	107.47	105.20
3	D	3	BTN	C2-C4-N2	-2.37	111.01	113.13
3	D	2	SGA	O3S-S-O2S	2.28	116.42	108.49
3	D	2	BTN	C2-C4-N2	-2.24	111.12	113.13

There are no chirality outliers.

All (3) torsion outliers are listed below:

Mol	Chain	Res	Type	Atoms
3	D	3	BTN	C2-C7-C8-C9
3	D	2	BTN	C7-C8-C9-C10
3	D	2	SGA	O5-C5-C6-O6

There are no ring outliers.

PRELIMINARY VALIDATION REPORT

1 monomer is involved in 1 short contact:

Mol	Chain	Res	Type	Clashes	Symm-Clashes
4	F	4	SO4	1	0

The following is a two-dimensional graphical depiction of Mogul quality analysis of bond lengths, bond angles, torsion angles, and ring geometry for all instances of the Ligand of Interest. In addition, ligands with molecular weight > 250 and outliers as shown on the validation Tables will also be included. For torsion angles, if less then 5% of the Mogul distribution of torsion angles is within 10 degrees of the torsion angle in question, then that torsion angle is considered an outlier. Any bond that is central to one or more torsion angles identified as an outlier by Mogul will be highlighted in the graph. For rings, the root-mean-square deviation (RMSD) between the ring in question and similar rings identified by Mogul is calculated over all ring torsion angles. If the average RMSD is greater than 60 degrees and the minimal RMSD between the ring in question and any Mogul-identified rings is also greater than 60 degrees, then that ring is considered an outlier. The outliers are highlighted in purple. The color gray indicates Mogul did not find sufficient equivalents in the CSD to analyse the geometry.

Ligand SGA D 2

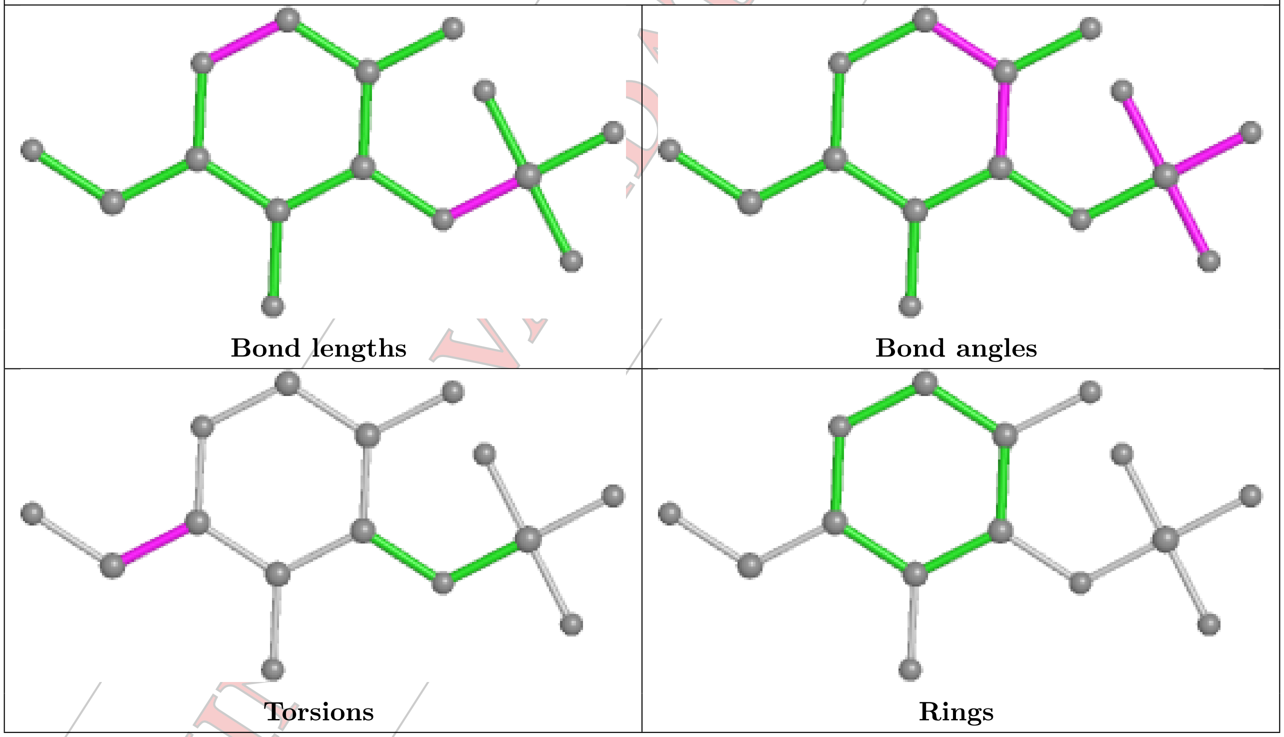

5.7 Other polymers (i)

There are no such residues in this entry.

PRELIMINARY VALIDATION REPORT

5.8 Polymer linkage issues (i)

There are no chain breaks in this entry.

PRELIMINARY VALIDATION REPORT

6 Fit of model and data (i)

6.1 Protein, DNA and RNA chains (i)

In the following table, the column labelled '#RSRZ> 2' contains the number (and percentage) of RSRZ outliers, followed by percent RSRZ outliers for the chain as percentile scores relative to all X-ray entries and entries of similar resolution. The OWAB column contains the minimum, median, 95th percentile and maximum values of the occupancy-weighted average B-factor per residue. The column labelled 'Q< 0.9' lists the number of (and percentage) of residues with an average occupancy less than 0.9.

Mol	Chain	Analysed	<RSRZ>	#RSRZ>2			OWAB(Å^2)	Q<0.9
1	A	172/173 (99%)	0.62	10 (5%)	23	25	22, 31, 49, 66	0
1	B	172/173 (99%)	0.57	6 (3%)	44	47	22, 31, 45, 75	0
All	All	344/346 (99%)	0.60	16 (4%)	31	34	22, 31, 48, 75	0

All (16) RSRZ outliers are listed below:

Mol	Chain	Res	Type	RSRZ
1	B	172	PRO	4.8
1	A	168	PRO	3.7
1	A	162	THR	3.0
1	A	164	ALA	2.9
1	B	169	SER	2.7
1	B	40	GLN	2.6
1	A	25	ALA	2.6
1	B	129	GLN	2.5
1	A	163	GLU	2.4
1	A	144	VAL	2.4
1	A	170	LYS	2.3
1	B	26	ALA	2.3
1	B	144	VAL	2.2
1	A	169	SER	2.1
1	A	155	ASN	2.1
1	A	172	PRO	2.1

6.2 Non-standard residues in protein, DNA, RNA chains (i)

There are no non-standard protein/DNA/RNA residues in this entry.

PRELIMINARY VALIDATION REPORT

6.3 Carbohydrates (i)

In the following table, the Atoms column lists the number of modelled atoms in the group and the number defined in the chemical component dictionary. The B-factors column lists the minimum, median, 95th percentile and maximum values of B factors of atoms in the group. The column labelled 'Q< 0.9' lists the number of atoms with occupancy less than 0.9.

Mol	Type	Chain	Res	Atoms	RSCC	RSR	B-factors(Å^2)	Q<0.9
3	BTN	D	2	16/?	0.84	0.19	48,54,71,73	0
2	NAG	D	1	15/?	0.89	0.15	22,22,22,22	0
2	NAG	C	1	15/?	0.92	0.17	22,22,22,22	0
2	SGA	C	2	15/?	0.93	0.15	22,22,22,22	0
3	SGA	D	2	15/?	0.97	0.11	22,22,22,22	0

The following is a graphical depiction of the model fit to experimental electron density for oligosaccharide. Each fit is shown from different orientation to approximate a three-dimensional view.

Electron density around Chain C:

2mF$_o$-DF$_c$ (at 0.7 rmsd) in gray
mF$_o$-DF$_c$ (at 3 rmsd) in purple (negative)
and green (positive)

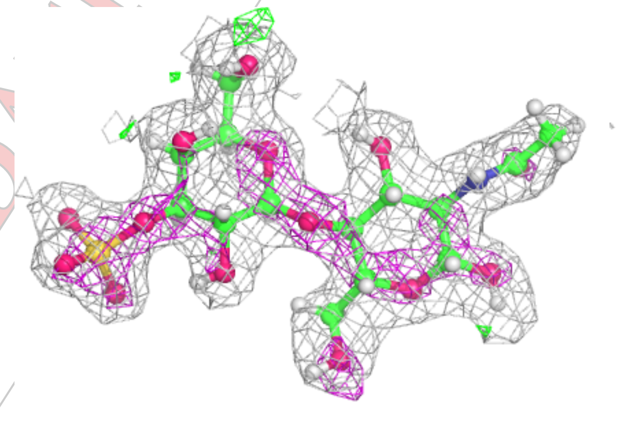

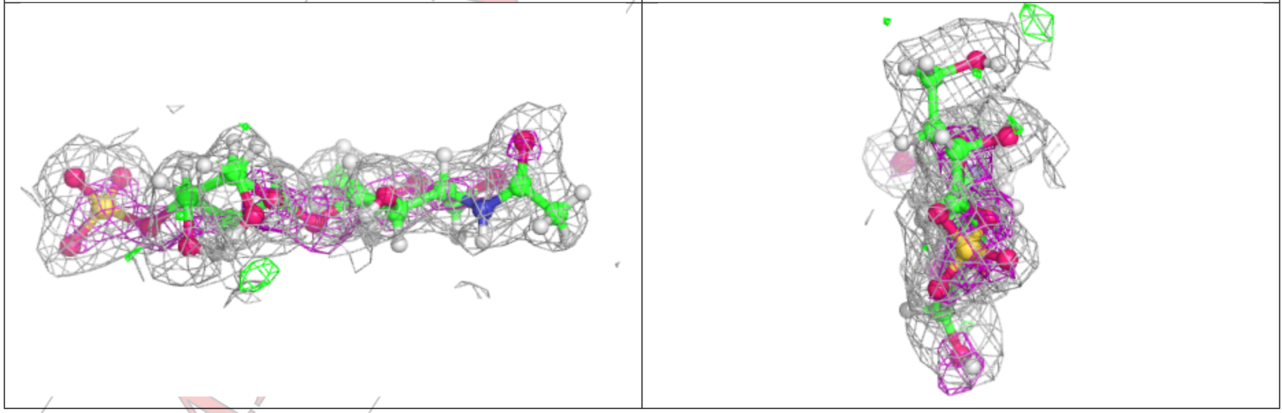

PRELIMINARY VALIDATION REPORT

Electron density around Chain D:

2mF$_o$-DF$_c$ (at 0.7 rmsd) in gray
mF$_o$-DF$_c$ (at 3 rmsd) in purple (negative)
and green (positive)

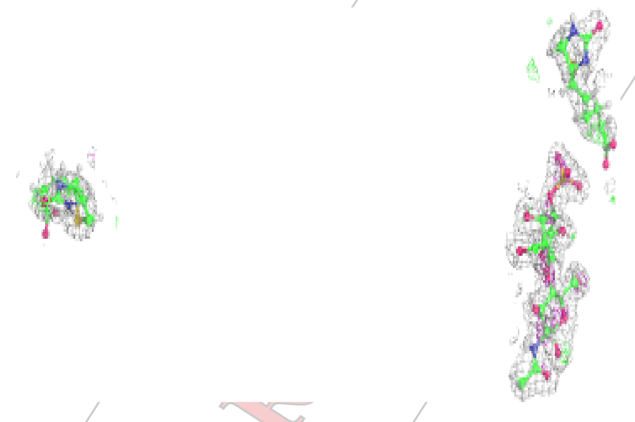

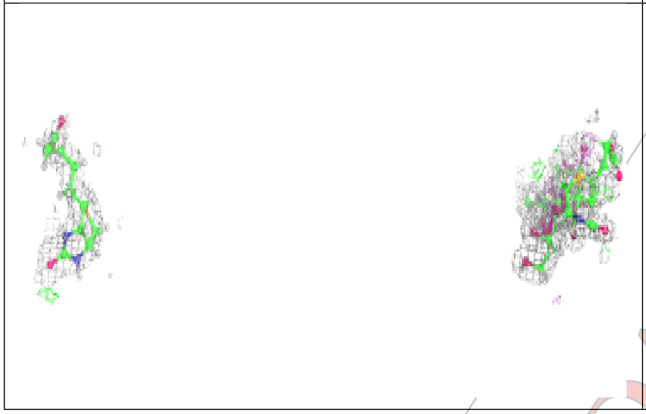

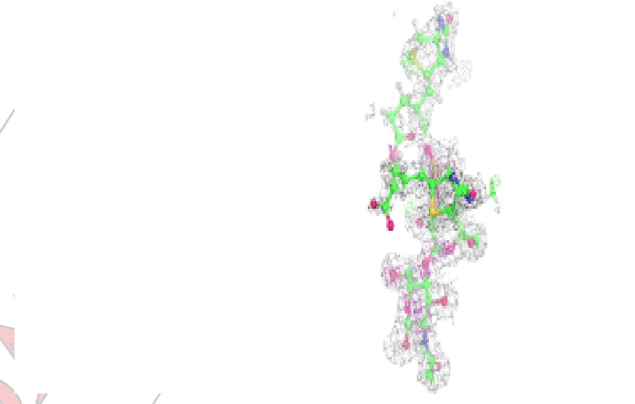

6.4 Ligands ⓘ

In the following table, the Atoms column lists the number of modelled atoms in the group and the number defined in the chemical component dictionary. The B-factors column lists the minimum, median, 95th percentile and maximum values of B factors of atoms in the group. The column labelled 'Q< 0.9' lists the number of atoms with occupancy less than 0.9.

Mol	Type	Chain	Res	Atoms	RSCC	RSR	B-factors(Å^2)	Q<0.9
4	SO4	F	3	5/?	0.76	0.23	104,104,105,105	0
3	BTN	D	3	16/?	0.80	0.24	43,50,74,78	0
3	BTN	D	2	16/?	0.84	0.19	48,54,71,73	0
4	SO4	F	4	5/?	0.90	0.31	67,71,72,75	0
4	SO4	F	1	5/?	0.93	0.17	73,74,76,77	0
4	SO4	F	2	5/?	0.93	0.18	73,73,74,74	0
3	SGA	D	2	15/?	0.97	0.11	22,22,22,22	0

The following is a graphical depiction of the model fit to experimental electron density of all instances of the Ligand of Interest. In addition, ligands with molecular weight > 250 and outliers as shown on the geometry validation Tables will also be included. Each fit is shown from different orientation to approximate a three-dimensional view.

PRELIMINARY VALID

Electron density around SGA D 2:

2mF$_o$-DF$_c$ (at 0.7 rmsd) in gray
mF$_o$-DF$_c$ (at 3 rmsd) in purple (negative)
and green (positive)

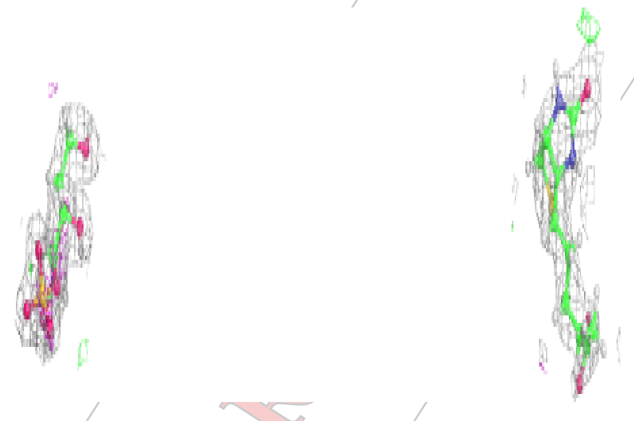

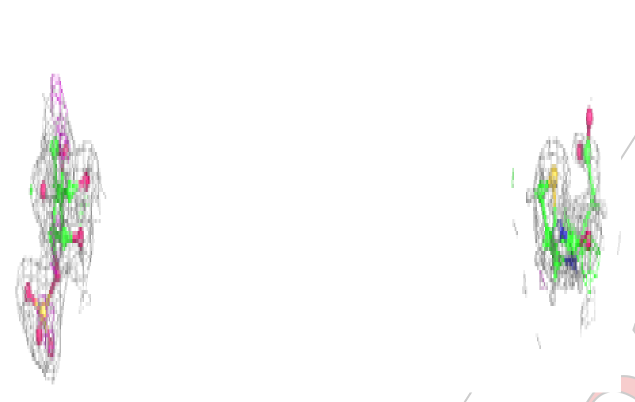

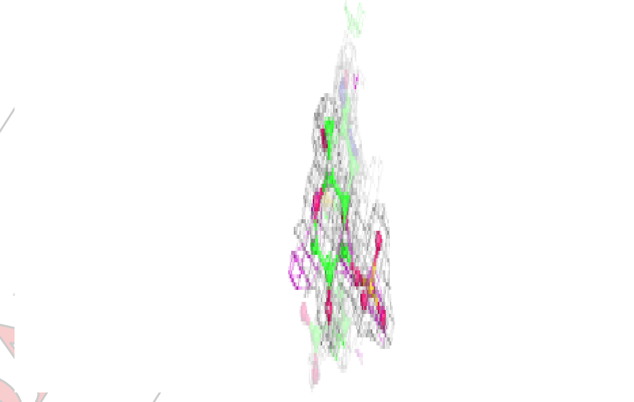

6.5 Other polymers (i)

There are no such residues in this entry.

PRELIMINARY VALIDATION
